# Consecutive and Selective Double Methylene Insertion of Lithium Carbenoids to Isothiocyanates: A Direct Assembly of Four‐Membered Sulfur‐Containing Cycles

**DOI:** 10.1002/anie.202110641

**Published:** 2021-10-13

**Authors:** Raffaele Senatore, Monika Malik, Thierry Langer, Wolfgang Holzer, Vittorio Pace

**Affiliations:** ^1^ University of Vienna Department of Pharmaceutical Sciences Althanstrasse, 14 A-1090 Vienna Austria; ^2^ University of Turin Department of Chemistry Via P. Giuria 7 10125 Turin Italy

**Keywords:** carbenoids, chemoselectivity, homologation, isothiocyanates, thietanes

## Abstract

A formal CH_2_−CH_2_ homologation conducted with C1 carbenoids on a carbon electrophile for the obtainment of a four‐membered cycle is reported. The logic proposes the consecutive delivery of two single nucleophilic CH_2_ units to an isothiocyanate—as competent electrophilic partner—resulting in the assembling of a rare imino‐thietane cluster. The single synthetic operation procedure documents genuine chemocontrol, as indicated by the tolerance to various reactive elements decorating the starting materials. Significantly, the double homologation protocol is accomplished directly on a carbon electrophile, thus not requiring the installation of heteroatom‐centered manifolds (e.g. boron).

## Graphical abstract

A formal CH_2_−CH_2_ homologation conducted with C1 carbenoids on a carbon electrophile for the obtainment of a four‐membered cycle is reported. Two single nucleophilic CH_2_ units are delivered to an isothiocyanate as competent electrophilic partner, resulting in the assembly of an imino‐thietane cluster. The double homologation protocol is accomplished directly on a carbon electrophile, not requiring the installation of heteroatom‐centered manifolds.

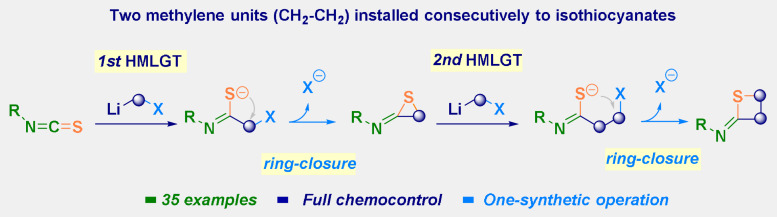

The progressive formal insertion of a constant methylene (CH_2_) unit into an organic array is known as homologation and, ultimately enables the modular assembly of complex skeletons,^[1]^ thus mimicking fundamental biosynthetic pathways.^[2]^ Regardless the essence of the adopted regime (nucleophilic, electrophilic or radical), the carbon skeleton elongation is limited to one methylene unit *per* homologation cycle, thus making multiple‐homologations highly elusive processes in synthesis (Scheme 1—*path a*).^[3]^ Although the intrinsic limited instability of reagents used for the purpose—for example, metal carbenoids^[4]^—may account for this reluctant behaviour, the installation of valuable linchpins on the chain results an effective solution to the problem. In this sense, the introduction by Matteson of boronic esters (**I**) as privileged electrophilic manifolds to conduct homologation with lithium carbenoids,^[5]^ underpinned the elaboration of the elegant concept of iterative homologations illustrated more recently by Aggarwal^[6]^ and Blakemore (Scheme 1—*path b*).^[7]^ Notably, this logic features remarkable flexibility and robust stereocontrol thus, enabling the use of (different) various C1‐units as competent donors, as documented in the elaboration of acyclic complex natural products.^[8]^ Notwithstanding, analogous elongations directly conducted on *carbon*‐centered manifolds remain underdeveloped and of limited preparative significance, regardless the hybridization of the competent carbon (*sp* ^*3*^ and *sp* ^*2*^).^[9]^ In recent years, our group developed modular homologations of *C*‐electrophiles with reactive lithium carbenoids (LiCH_2_X) enabling the assembly of sophisticated structures, as a consequence of molecular rearrangements triggered by the fine tuning of reaction conditions.[^3^, ^10^] In this context, we documented the halogen‐driven selective homologations of trifluoroacetimidoyl chlorides (TFAIC, **I**) to *gem*‐chloro‐trifluoromethylaziridines (**II**) or *gem*‐halomethyl‐trifluoromethylaziridines (**III**)^[11]^ and bis‐trifluoromethyl‐β‐diketiminates (**IV**)^[12]^ respectively, highlighting the critical role played by the reaction stoichiometry in discriminating between C1 and C1‐C1 insertions (Scheme 1—*path c*). Conceptually, these precedents could be ascribed to the well‐established carbonyl‐oxirane (and related imine‐aziridine) homologative chemistry:^[13]^ this is, the cyclic cluster is constructed via the incorporation of a *single* C1 unit delivered by the carbenoid donor.^[14]^ We wondered if homologation tactics with C1 carbenoids could promote the assembly of four‐membered cycles^[15]^ through the consecutive release of *two* methylene fragments on a proper electrophile. Crucial for the successful reaction design was ensuring that the homologation sequence could reach completion within the short lifetime of the carbenoid used.^[16]^ To this end, we conceived to generate a *first* homologation reactive intermediate susceptible of rapid *second* CH_2_‐insertion, furnishing the targeted four‐membered cycle.^[17]^ Ideally, an electrophilic platform fulfilling this criterion could be represented by an isothiocyanate (**V**): in fact, the formation of a nucleophilic thioamidate anion (**VI**)—after the first homologative event—would induce a fast cyclization to a highly reactive thiiranimine (**VII**),^[18]^ which would then undergo the second homologation. Finally, the additional ring closure of the elongated thioamidate (**VIII**) presenting the inserted CH_2_−CH_2_ motif would result in the thietane scaffold (**IX**) presenting the *exo* imine‐appendix (Scheme 1—*path d*).

**Scheme 1:**
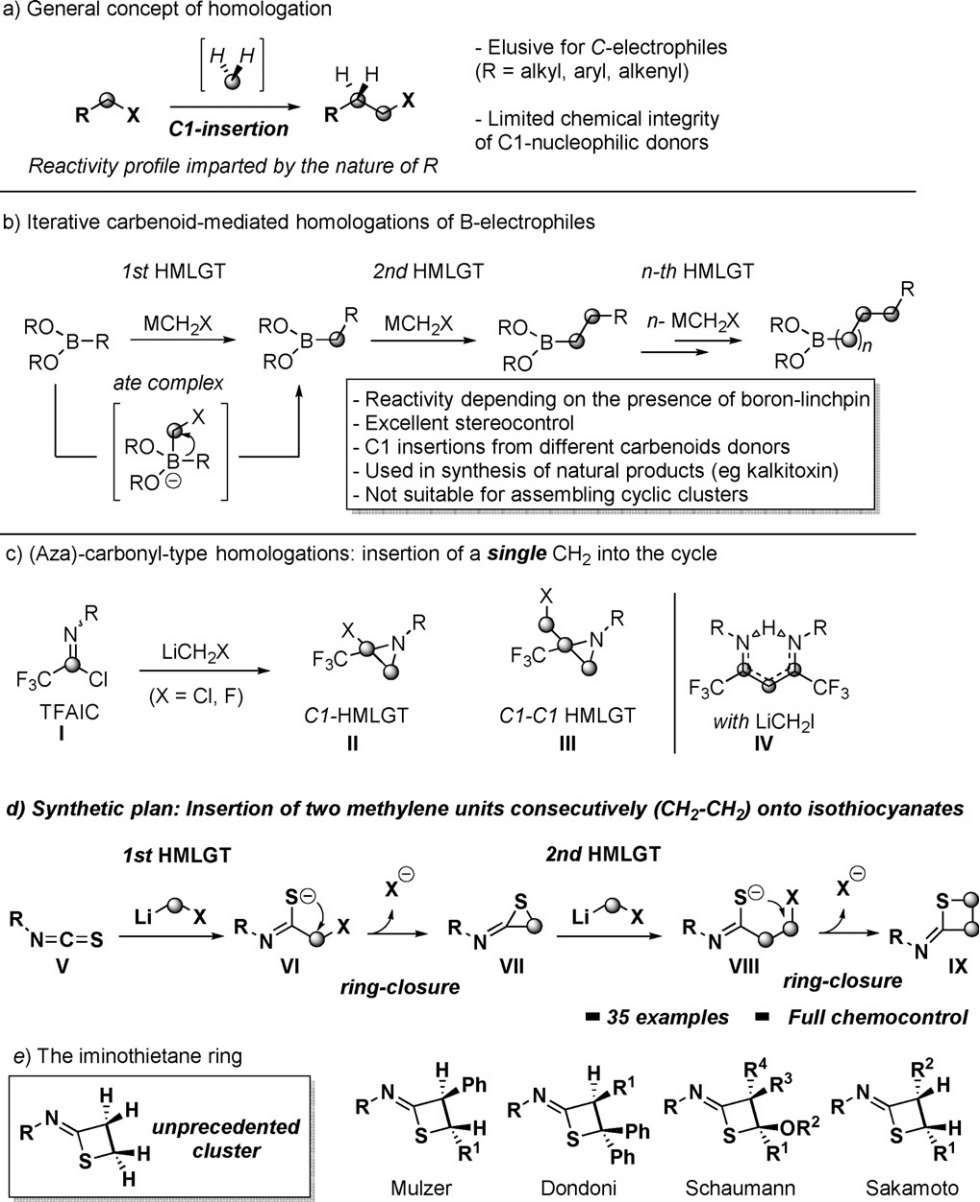
General context of the presented work (HMLGT=homologation).

Collectively, our synthetic strategy would construct the rare imino‐thietane skeleton through a conceptually intuitive logic starting from commercially available (or easily accessible) isothiocyanates. We anticipate the proposed direct approach conducts to the imino‐thietane core presenting an integral CH_2_−CH_2_ linkage. To the best of our knowledge, such a structural characteristic is unprecedented among the scattered imino‐thietanes previously described (Scheme 1—*path e*). Indeed, uniformly—the recyclization of β‐lactones (Mulzer),^[19]^ the cycloaddition of ketenimines to thioketones (Dondoni),^[20]^ the cycloaddition of vinyl ethers with sulfonyl isothiocyanates (Schaumann),^[21]^ and, the photochemical isomerization of *N*‐monosubstituted α,β‐unsaturated thioamides (Sakamoto)^[22]^—afforded imino‐thietanes variously substituted on the C−C linkage.^[23]^

4‐Ethoxyisothiocyanate **1** was selected as the model substrate for the reaction optimization (Table 1). Upon treatment with the carbenoid chloromethyllithium (LiCH_2_Cl), only a complex mixture was obtained, even at very short reaction times (entries 1,2).^[24]^ Because of the absence in the mixture of the product resulting from the attack of MeLi‐LiBr to the electrophilic isothiocyanate (i.e. thioamide **3**), it could be assumed that the carbenoid was effectively generated. The switching to the highly unstable LiCH_2_F^[25]^ enabled to assembly the targeted four‐membered cycle **2**, thus validating the initial working hypothesis (entry 3). It was essential to quench the reaction after only 5 min (from the end of the addition of MeLi‐LiBr) to ensure a clean crude, thus confirming the high reactivity of intermediates conceived during the process design (entries 4,5). Notably, the overall transformation is in agreement with the observation by J. Hu on the suitability of CH_2_‐F fragments as manifolds for nucleophilic substitutions.^[26]^ Additional improvement was achieved employing LiCH_2_Br, which served as a competent C1 delivery agent for constructing the thietane backbone **2** (entries 6) in an excellent 82 % isolated yield. Confirming the pattern observed with LiCH_2_F (entries 4,5), also the reaction with the bromo carbenoid benefited from brief reaction time (entry 6 vs. 7), presumably because of avoiding the triggering of deleterious carbenoids decomposition events. Although presenting a better halogen leaving‐group, the use of LiCH_2_I resulted in slightly dwindled yield (entry 8) presumably due to its tendency to act also as metalating agent.^[12]^ Some additional points merit mention: a) in line with our previous investigations, the optimal LiCH_2_Br precursor proved to be ICH_2_Br which undergoes a rapid and efficient I/Li exchange,^[27]^ thus avoiding the detection of thioamide **2 a**,^[28]^ formed at significant extent in the presence of CH_2_Br_2_ (entry 9); b) conducting the process at higher temperature resulted in untractable mixtures because of compromised chemical integrity of carbenoids (entry 10); c) running the consecutive homologation in more apolar solvents (diethyl ether, toluene—entries 11,12), dramatically decreased reaction yields; d) considerable excess of nucleophile did not boost the transformation (entry 13), while using quasi‐stoichiometric loading dropped the yield (entry 14); e) no reactivity was displayed by less nucleophilic magnesium carbenoids, even at higher temperatures (entries 15,16).^[29]^

**Table 1:** Reaction optimization. 
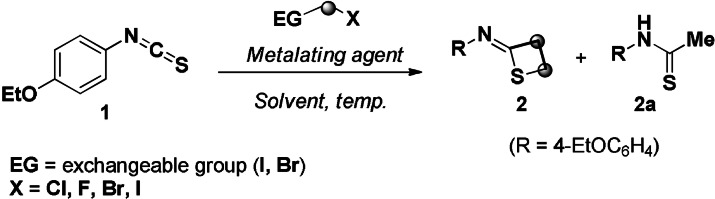

Entry	Carbenoid precursor (equiv)^[a]^	Metalating agent	Reaction time [min]	Isolated yield of **2** [%]
1	ClCH_2_I (3.0)	MeLi‐LiBr (2.8)	5	Compl. mix.
2	ClCH_2_I (3.0)	MeLi‐LiBr (2.8)	60	Compl. mix.
3^[b]^	FCH_2_I (3.0)	MeLi‐LiBr (2.8)	5	69
4^[b]^	FCH_2_I (3.0)	MeLi‐LiBr (2.8)	15	45
5^[b]^	FCH_2_I (3.0)	MeLi‐LiBr (2.8)	60	Compl. mix.
6	BrCH_2_I (3.0)	MeLi‐LiBr (2.8)	5	82
7	BrCH_2_I (3.0)	MeLi‐LiBr (2.8)	15	54
8	I_2_CH_2_ (3.0)	MeLi‐LiBr (2.8)	5	71
9^[c]^	Br_2_CH_2_ (3.0)	MeLi‐LiBr (2.8)	5	65
10^[d]^	BrCH_2_I (3.0)	MeLi‐LiBr (2.8)	5	Compl. mix.
11^[e]^	BrCH_2_I (3.0)	MeLi‐LiBr (2.8)	5	9
12^[f]^	BrCH_2_I (3.0)	MeLi‐LiBr (2.8)	5	Traces
13	BrCH_2_I (6.0)	MeLi‐LiBr (5.8)	5	80
14^[g]^	BrCH_2_I (1.3)	MeLi‐LiBr (1.1)	5	27
15	BrCH_2_I (2.8)	*i*‐PrMgCl‐LiCl	5	No reaction
16^[d]^	BrCH_2_I (2.8)	*i*‐PrMgCl‐LiCl	5	No reaction

[a] LiCH_2_X reagents were generated under Barbier conditions. [b] LiCH_2_F was generated in a 1:1 *v*/*v* mixture of THF and Et_2_O (ref. 25). [c] Thioamide **3** was also formed in 21 % isolated yield. [d] Reaction run at −40 °C. [e] Reaction run in Et_2_O. [g] Reaction run in toluene. [g] No mono‐homologated product (type **VII**—Scheme 1) was detected.

The unambiguous assignment of the imino‐thietane backbone was ascertained via X‐ray crystallographic analysis of compound **2** (Figure 1). The very small value of torsion angles C7‐S1‐C9‐C8 (0.76°), C9‐C8‐C7‐S1 (0.91°) and C1‐N1‐C7‐S1 (1.86°) are diagnostic for the almost complete planarity of the ring, thus differentiating it from a classical non‐planar thietane characterized by a puckering angle of 26°.^[30]^ The internal angles C9‐C8 C8‐C7 (84.65°), S1‐C9 C9‐C8 (87.65°), C7‐S1 S1‐C9 (77.01°) alternating from the *sp* ^*3*^ geometry and, S1‐C7 C7‐C8 (95.27°) deviating from a canonical C‐*sp* ^*2*^ confirm the formation of the thietane ring. The analysis of bond lengths indicates that the S1‐C9 bond (1.835 Å) is significantly longer than an average C(*sp* ^3^)‐S linkage (1.817 Å),^[31]^ but slightly shorter than the C‐S bond (1.847 Å) of the unsubstituted thietane.^[30]^ The constructed C8‐C9 bond (1.541 Å) is in the average of the analogous CH_2_−CH_2_ linkage found in the simple thietane (1.549 Å).^[30]^

**Figure 1:**
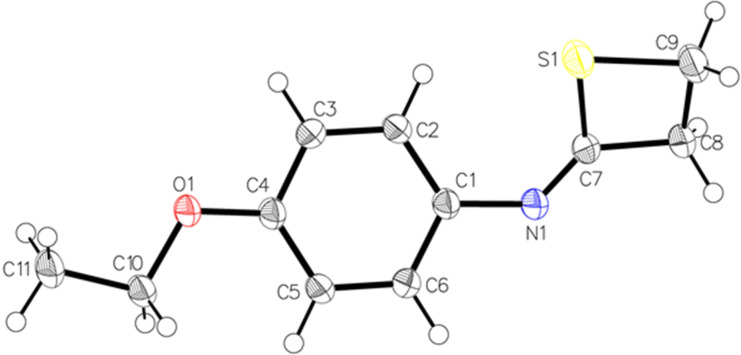
X‐ray structural analysis of compound **2**.^[35]^

Having established the optimal conditions for the transformation, we next studied the scope of the consecutive double CH_2_ insertion (Scheme 2). The presence of electron‐donating groups—that is, ethers—on the aromatic nucleus of the isothiocyanates is uniformly tolerated, furnishing the corresponding imino‐thietanes (**2**–**5**) in very good yields, including when the ethereal oxygen presented phenyl‐ (**6**) or trifluoro‐ (**7**) functionalities. Running the protocol on bigger scale (20 mmol) resulted in comparable efficiency (**2**). An acetal‐substituted isothiocyanate, as exemplified by the benzo[*d*][1,3]dioxole structure (**8**), is also amenable for the protocol. Chalcogens such as sulfur—embodied as a thioether (**9**) or, selenium—in the case of the selenoether (**10**), also act as productive starting materials. Notably, the adopted lithiation conditions for the carbenoid genesis do not activate a concomitant Se/Li exchange thus, leaving untouched this functionality. Aside of an unsubstituted phenyl ring (**11**), the incorporation of halogen such as fluorine (**12**, **17**, **18**), bromine (**13**), chlorine (**14**–**17**) at different positions of the ring are compatible with the methodology and, their presence could be advantageously exploited for further elaboration (vide infra). *sp* ^*3*^‐Hybridized hydrocarbons do not alter the efficiency of the technique (**19**–**21**), including when an *i*‐propyl group (**22**) or the bicyclic indanyl system (**23**) were installed on the heterocumulene starting material. Preparing imino‐thietanes with the proposed strategy proceeded with remarkable chemocontrol, as documented by isothiocyanates decorated with unsaturated residues: no concomitant carbenoid‐mediated cyclopropanation^[32]^ took place on a vinyl‐substituted analogue (**24**), neither deprotonation of a terminal alkyne (**25**) despite the basic conditions. Furthermore, the chemical integrity of electrophilic substituents potentially suffering the attack of nucleophilic carbenoids was preserved, as deducted by the ester (**26**) and the nitrile (**27**). The methodology showed that the presence of nitrogen‐centered substituents—in principle able to invalidate the correct carbenoid formation—was not detrimental: accordingly, *N*,*N*‐dimethylamino‐ (**28**), morpholino‐ (**29**), phenyldiazo‐ (**30**) and azido‐ (**31**) bearing isothiocyanates were consecutively homologated to afford the corresponding four‐membered sulfur‐ring. Intriguingly, 1,4‐diisothiocyanatobenzene was subjected to selective assembly of the cycle on a single NCS appendix (**32**). This same pattern was confirmed also with a considerable excess of carbenoid (6.0 equiv). Finally, the flexibility to heteroaromatic systems was documented with the 3‐pyridyl derivative, which furnished the desired motif in comparable good yield (**33**). The effective release of the two carbons backbone C−C from the carbenoid source was gathered conducting the experiment—*coeteris paribus*—with di‐deuterated iodomethyllithium (LiCD_2_I) conveniently prepared from the commercially available CD_2_I_2_.^[10]^ The evidence for the ethylene fragment in **34** featuring full deuteration—that is, CD_2_−CD_2_—was evidently consistent with the envisaged rationale.

**Scheme 2:**
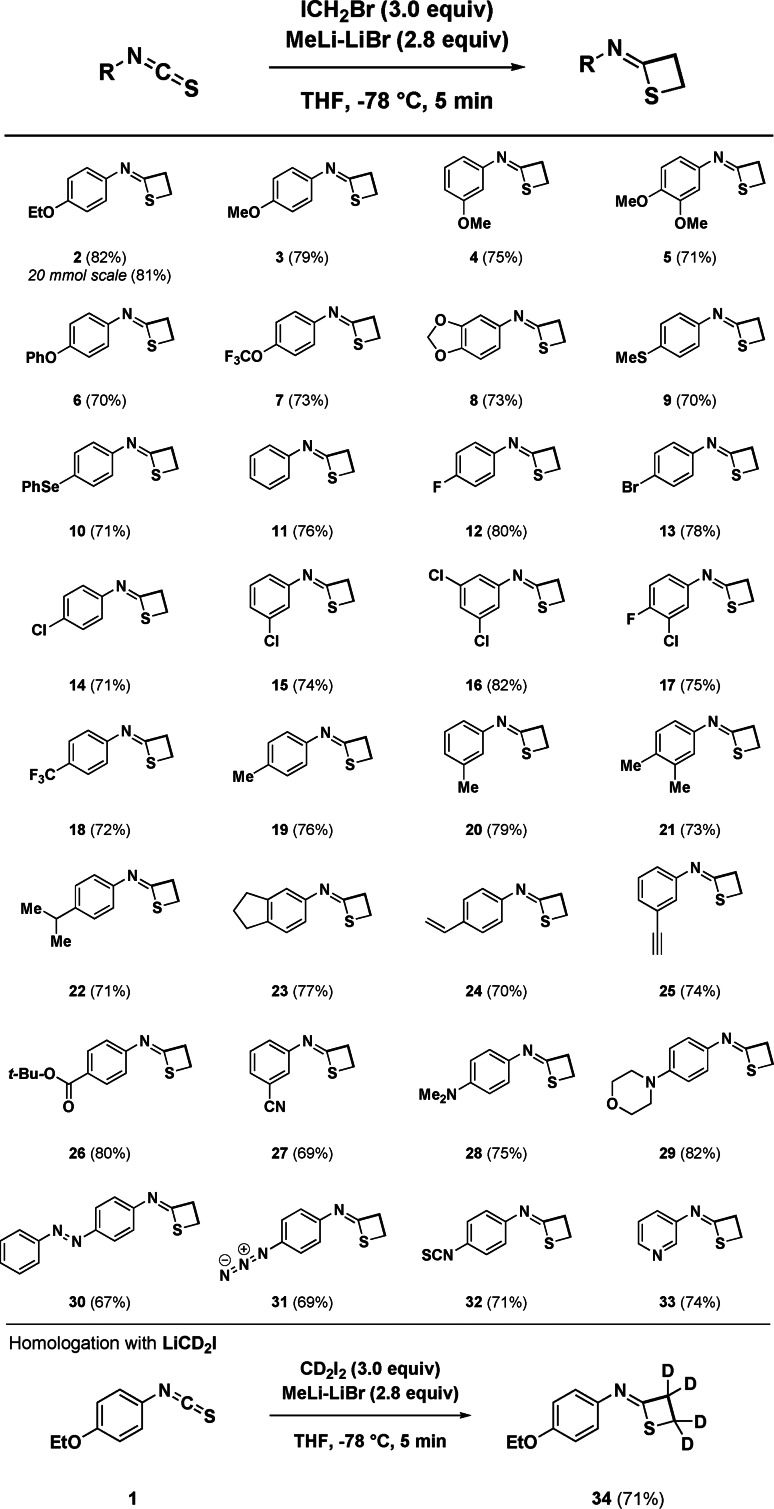
Scope of the consecutive CH_2_−CH_2_ homologation.

Eager to understand the reactivity of the assembled system, we conducted manipulations both on the aromatic ring (proceeding from the parent isothiocyanate) and on the four‐membered cycle (Scheme 3). The Buchwald‐Hartwig amination^[33]^ with piperidine on the bromo‐analogue **13** cleanly furnished the corresponding product **35** in which the thioimidate system was preserved (*path a*). Cognizant of the ring strain of four‐membered rings (e.g. thietane 80.3 kJ mol^−1^),^[34]^ we devised the ring‐opening with a nucleophile levering on the electrophilicity of the *sp* ^*2*^‐carbon (*path b*). Thus, via the addition of a reducing hydride—accomplished with LiAlH_4_—γ‐amino‐mercaptanes **XII** were formed in high yield, as demonstrated by the straightforward synthesis of different analogues (**36**–**39**). The transformation can be rationalized through the formation of a tetrahedral intermediate (**X**), whose collapse entails the breaking of the NC‐S bond giving the γ‐*N*‐phenyliminopropyl mercapto anion (**XI**) amenable for a second hydride‐reduction *en route* to the final, not previously accessible, structures (**XII**). The experiment with labelled LiAlD_4_ (**37**) confirmed the attack of both nucleophiles at the initially thioimidate carbon. Additional evidence for the chemical integrity of the unsubstituted iminothietanes was deducted by attempting the ring opening under different conditions, thus resulting in no modification of the nuclei (see Supporting Information).

**Scheme 3:**
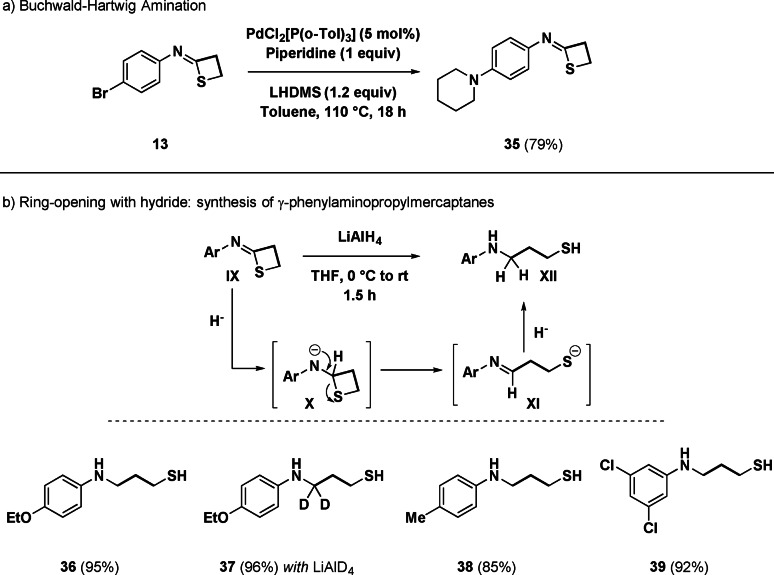
Reactivity profile of unsubstituted imino‐thietanes.

In summary, we have documented the installation of the ethylene unit (−CH_2_−CH_2_−)—a formal C2‐homologation—onto isothiocyanates through the consecutive delivery of two methylene fragments furnished by the carbenoid bromomethyllithium (LiCH_2_Br). The tactic enables the straightforward access to four‐membered cyclic imino‐thietanes through a single synthetic operation. The overall strategy relies on two sequential homologative events commenced on the electrophilic *sp*‐carbon of a competent isothiocyanate, resulting in the formation—upon ring‐closure—of a highly reactive imino‐thirane which then undergoes a second homologation giving a γ‐halothioimidate direct precursor of the final imino‐thietane. A remarkable degree of chemoselectivity was uniformly observed, as evidenced by running the reaction of isothiocyanates featuring additional sensitive functionalities such as halogens, seleno‐, thio‐ethers, amines or electrophilic groups susceptible of the attack of the nucleophilic carbenoid. The assembled four‐membered imino‐thioethane—whose structure was ascertained by X‐ray analysis—may serve as a versatile platform for preparing γ‐aminopropylthiols via hydride‐mediated reduction. To the best of our knowledge this concept constitutes the first example of a controlled consecutive C1‐homologation on carbon electrophiles with a carbenoid without the requirement of hetero‐atom linchpins (e.g. boron).

## Conflict of interest

The authors declare no conflict of interest.

## Supporting information

As a service to our authors and readers, this journal provides supporting information supplied by the authors. Such materials are peer reviewed and may be re‐organized for online delivery, but are not copy‐edited or typeset. Technical support issues arising from supporting information (other than missing files) should be addressed to the authors.

Supporting Information
