# Supplementary material for: Consecutive and Selective Double Methylene Insertion of Lithium Carbenoids to Isothiocyanates: A Direct Assembly of Four‐Membered Sulfur‐Containing Cycles
Source: Angew Chem Int Ed Engl. 2021 Oct 13;60(47):24854–8. doi: 10.1002/anie.202110641 (PMC9293044; doi:10.1002/anie.202110641)
Supplement: Supplementary file 1 — Supporting Information [file ANIE-60-24854-s001.pdf]

Angewandte Chemie
Eine Zeitschrift der Gesellschaft Deutscher Chemiker

Supporting Information

Consecutive and Selective Double Methylene Insertion of Lithium Carbenoids to Isothiocyanates: A Direct Assembly of Four-Membered Sulfur-Containing Cycles

*Raffaele Senatore, Monika Malik, Thierry Langer, Wolfgang Holzer, and Vittorio Pace**

anie_202110641_sm_miscellaneous_information.pdf

SUPPORTING INFORMATION

TABLE OF CONTENTS

Instrumentation and General Analytical Methods	S2
General Procedure	S2
Characterization and Spectral Data of the Compounds	S3
Copies of ^{1}H- and ^{13}C-NMR Spectra	S44
Copies of ^{19}F- and ^{77}Se-NMR Spectra	S84
X-Ray Analysis for compound 2	S88
References	S90

Instrumentation and General Analytical Methods

Melting points were determined on a Reichert–Kofler hot-stage microscope and are uncorrected. Mass spectra were obtained on a Shimadzu QP 1000 instrument (EI, 70 eV) and on a Bruker maXis 4G instrument (ESI-TOF, HRMS). ^{1}H, ^{13}C, ^{15}N, ^{19}F and ^{77}Se NMR spectra were recorded with a Bruker Avance III 400 spectrometer (400 MHz for ^{1}H, 100 MHz for ^{13}C, 40 MHz for ^{15}N, 376 MHz for ^{19}F and 76 MHz for ^{77}Se) at 297 K using a directly detecting broadband observe (BBFO) probe. The center of the (residual) solvent signal was used as an internal standard which was related to TMS with δ 7.26 ppm (^{1}H in $CDCl_3$), 7.16 ppm (^{1}H in C_6D_6) and δ 77.0 ppm (^{13}C in $CDCl_3$), 128.06 (^{13}C in C_6D_6). ^{15}N NMR (gs-HMBC) spectra were referenced against neat, external nitromethane. ^{19}F NMR spectra were referenced via the Ξ ratio (absolute referencing). ^{77}Se spectra were referenced against diphenyldiselane (δ Ph_2Se_2 463 ppm). Spin-spin coupling constants (*J*) are given in Hz.
In nearly all cases, full and unambiguous assignment of all resonances was performed by combined application of standard NMR techniques, such as APT, HSQC, HMBC, HSQCTOCSY, COSY and NOESY experiments.

All reactions were performed under an inert atmosphere of argon using standard schlenk techniques. THF was distilled over Na/benzophenone. Starting isothiocyanates were commercially available or prepared as reported. Fluoroiodomethane was supplied from ABCR Germany. Other chemicals were purchased from SigmaAldrich, Acros, Alfa Aesar, Fluorochem and TCI Europe. Solutions were evaporated under reduced pressure with a rotary evaporator. For column chromathography, silica Gel 60 (0.04-0.063 mm) was used. TLC was carried out on aluminium sheets precoated with silica gel 60F254 (Merchery-Nagel, Merk); the spots were visualised under UV light (λ = 254 nm) and/or $KMnO_4$ (aq.) was used as revealing system.

General Procedure for the synthesis of four-membered imino-thietanes.

To a solution of the starting isothiocyanate (1.0 equiv) in dry THF (3 mL) bromoiodomethane (3.0 equiv) was added. Then, a solution of MeLi-LiBr (2.2 M in Et_2O, 2.8 equiv) was added dropwise at -78 °C. After stirring for 5 min at -78 °C, the reaction was quenched with sat. aqueous NH_4Cl. The reaction mixture was allowed to reach room temperature and exhaustively extracted with Et_2O (3 x 10 ml). The combined organic extracts were washed with brine, dried over Na_2SO_4 and the solvent was removed under reduced pressure. The final products were obtained after purification by column chromatography on silica gel.

Characterization and Spectral Data of the Compounds

4-ethoxy-*N*-[(2*Z*)-2-thietanylidene]aniline (2)

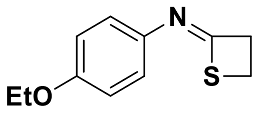

By following the general procedure, starting from 4-ethoxyphenyl isothiocyanate (0.179 g, 1.0 mmol, 1.0 equiv), bromoiodomethane (0.662 g, 0.22 mL, 3.0 mmol, 3.0 equiv) and MeLi-LiBr (2.2 M in Et_2O, 1.27 mL, 2.8 mmol, 2.8 equiv) in dry THF (3 mL), compound **2** was obtained in 82% yield (0.170 g) as a brown solid (mp: 62-65 °C) after purification by column chromatography on silica gel (*n*-hexane:EtOAc 8:2).

Scaling-up of the reaction (20 mmol) - By following the general procedure, starting from 4-ethoxyphenyl isothiocyanate (3.580 g, 20.0 mmol, 1.0 equiv), bromoiodomethane (13.250 g, 4.52 mL, 60.0 mmol, 3.0 equiv) and MeLi-LiBr (2.2 M in Et_2O, 25.45 mL, 56.0 mmol, 2.8 equiv) in dry THF (60 mL), compound **2** was obtained in 81% yield (3.358 g) as a brown solid after purification by column chromatography on silica gel (*n*-hexane:EtOAc 8:2). *Spectroscopic and spectrometric data match with those reported for the 1.0 mmol scale*

^{1}H NMR (400 MHz, C_6D_6) δ: 7.24 (m, 2H, Ph H-2,6), 6.81 (m, 2H, Ph H-3,5), 3.54 (q, ^3J = 7.0 Hz, 2H, OCH_2), 3.33 (m, 2H, CH_2), 2.39 (m, 2H, SCH_2), 1.08 (t, ^3J = 7.0 Hz, 3H, CH_3).

^{13}C NMR (100 MHz, C_6D_6) δ: 158.0 (C=N), 157.1 (Ph C-4), 141.4 (Ph C-1), 122.7 (Ph C-2,6), 115.4 (Ph C-3,5), 63.4 (OCH_2), 47.1 (CH_2), 20.4 (SCH_2), 14.9 (CH_3).

^{15}N NMR (40 MHz, C_6D_6) δ: -82.5 (C=N).

HRMS (ESI), *m/z*: calcd. for $C_{11}H_{14}NOS^+$: 208.0791 $[M + H]^+$; found: 208.0792.

***N*-(4-ethoxyphenyl)ethanethioamide (2a)**

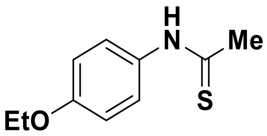

s-*cis* rotamer

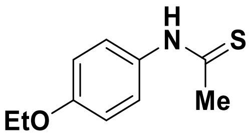

s-*trans* rotamer

Obtained in 21% yield (0.041 g) as a yellowish solid (mp: 114 °C), as reported in Table 1 of the manuscript (Entry 9).

s-*trans* : s-*cis* ~ 1 : 1

s-*cis* rotamer:

^{1}H NMR (400 MHz, $CDCl_3$) δ: 8.59 (br s, 1H, NH), 7.50 (m, 2H, Ph H-2,6), 6.90 (m, 2H, Ph H-3,5), 4.03 (q, ^3J = 7.0 Hz, 2H, $OC\underline{H}_2CH_3$), 2.72 (s, 3H, CH_3), 1.41 (t, ^3J = 7.0 Hz, 3H, $OCH_2C\underline{H}_3$).

^{13}C NMR (100 MHz, $CDCl_3$) δ: 200.3 (C=S), 157.7 (Ph C-4), 130.8 (Ph C-1), 125.7 (Ph C-2,6), 114.6 (Ph C-3,5), 63.7 ($O\underline{C}H_2CH_3$), 35.8 (CH_3), 14.8 ($OCH_2\underline{C}H_3$).

^{15}N NMR (40 MHz, $CDCl_3$) δ: -219.5 (NH).

s-*trans* rotamer:

^{1}H NMR (400 MHz, $CDCl_3$) δ: 9.31 (br s, 1H, NH), 7.08 (m, 2H, Ph H-2,6), 6.90 (m, 2H, Ph H-3,5), 4.04 (q, ^3J = 7.0 Hz, 2H, $OC\underline{H}_2CH_3$), 2.45 (s, 3H, CH_3), 1.43 (t, ^3J = 7.0 Hz, 3H, $OCH_2C\underline{H}_3$).

^{13}C NMR (100 MHz, $CDCl_3$) δ: 205.0 (C=S), 158.6 (Ph C-4), 131.5 (Ph C-1), 126.8 (Ph C-2,6), 115.2 (Ph C-3,5), 63.8 ($O\underline{C}H_2CH_3$), 29.8 (CH_3), 14.7 ($OCH_2\underline{C}H_3$).

^{15}N NMR (40 MHz, $CDCl_3$) δ: -217.4 (NH).

HRMS (ESI), *m/z*: calcd. for $C_{10}H_{14}NOS^+$: 196.0791 $[M + H]^+$; found: 196.0792.

4-methoxy-*N*-[(2*Z*)-2-thietanylidene]aniline (3)

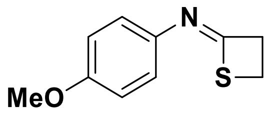

By following the general procedure, starting from 4-methoxyphenyl isothiocyanate (0.165 g, 1.0 mmol, 1.0 equiv), bromoiodomethane (0.662 g, 0.22 mL, 3.0 mmol, 3.0 equiv) and MeLi-LiBr (2.2 M in Et_2O, 1.27 mL, 2.8 mmol, 2.8 equiv) in dry THF (3 mL), compound **3** was obtained in 79% yield (0.152 g) as a brown oil after purification by column chromatography on silica gel (*n*-hexane:EtOAc 8:2).

^{1}H NMR (400 MHz, C_6D_6) δ: 7.22 (m, 2H, Ph H-2,6), 6.77 (m, 2H, Ph H-3,5), 3.33 (m, 2H, CH_2), 3.26 (s, 3H, OCH_3) 2.39 (m, 2H, SCH_2).

^{13}C NMR (100 MHz, C_6D_6) δ: 158.2 (C=N), 157.7 (Ph C-4), 141.6 (Ph C-1), 122.7 (Ph C-2,6), 114.9 (Ph C-3,5), 54.9 (OCH_3), 47.0 (CH_2), 20.4 (SCH_2).

^{15}N NMR (40 MHz, C_6D_6) δ: -82.0 (C=N).

HRMS (ESI), *m/z*: calcd. for $C_{10}H_{12}NOS^+$: 194.0634 $[M + H]^+$; found: 194.0640.

3-methoxy-*N*-[(2*Z*)-2-thietanylidene]aniline (4)

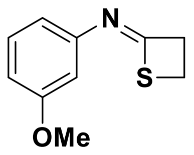

By following the general procedure, starting from 3-methoxyphenyl isothiocyanate (0.165 g, 1.0 mmol, 1.0 equiv), bromoiodomethane (0.662 g, 0.22 mL, 3.0 mmol, 3.0 equiv) and MeLi-LiBr (2.2 M in Et_2O, 1.27 mL, 2.8 mmol, 2.8 equiv) in dry THF (3 mL), compound **4** was obtained in 75% yield (0.144 g) as a yellow oil after purification by column chromatography on silica gel (*n*-hexane:EtOAc 8:2).

^{1}H NMR (400 MHz, C_6D_6) δ: 7.11 (m, 1H, Ph H-5), 6.91 (m, 1H, Ph H-2), 6.90 (m, 1H, Ph H-6), 6.62 (m, 1H, Ph H-4), 3.30 (s, 3H, OCH_3), 3.27 (m, 2H, CH_2), 2.33 (m, 2H, SCH_2).

^{13}C NMR (100 MHz, C_6D_6) δ: 161.2 (Ph C-3), 160.7 (C=N), 150.0 (Ph C-1), 130.4 (Ph C-5), 113.4 (Ph C-6), 111.3 (Ph C-4), 107.1 (Ph C-2), 54.8 (OCH_3), 46.7 (CH_2), 20.1 (SCH_2).

^{15}N NMR (40 MHz, C_6D_6) δ: -79.4 (C=N).

HRMS (ESI), *m/z*: calcd. for $C_{10}H_{12}NOS^+$: 194.0634 $[M + H]^+$; found: 194.0639.

3,4-dimethyl-*N*-[(2*Z*)-2-thietanylidene]aniline (5)

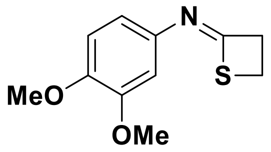

By following the general procedure, starting from 3,4-dimethoxyphenyl isothiocyanate (0.195 g, 1.0 mmol, 1.0 equiv), bromoiodomethane (0.662 g, 0.22 mL, 3.0 mmol, 3.0 equiv) and MeLi-LiBr (2.2 M in Et_2O, 1.27 mL, 2.8 mmol, 2.8 equiv) in dry THF (3 mL), compound **5** was obtained in 71% yield (0.158 g) as a brown oil after purification by column chromatography on silica gel (*n*-hexane:EtOAc 6:4).

^{1}H NMR (400 MHz, C_6D_6) δ: 6.88 (dd, ^3J = 8.3 Hz, ^4J = 2.3 Hz, 1H, Ph H-6), 6.85 (d, ^4J = 2.3 Hz, 1H, Ph H-2), 6.59 (d, ^3J = 8.3 Hz, 1H, Ph H-5), 3.40 (s, 3H, 3-OCH_3), 3.38 (s, 3H, 4-OCH_3), 3.37 (m, 2H, CH_2), 2.43 (m, 2H, SCH_2).

^{13}C NMR (100 MHz, C_6D_6) δ: 158.3 (C=N), 150.8 (Ph C-3), 147.8 (Ph C-4), 142.0 (Ph C-1), 112.9 (Ph C-5), 112.8 (Ph C-6), 106.8 (Ph C-2), 55.8 (4-OCH_3), 55.4 (3-OCH_3), 47.0 (CH_2), 20.5 (SCH_2).

HRMS (ESI), *m/z*: calcd. for $C_{11}H_{14}NO_2S^+$: 224.0740 $[M + H]^+$; found: 224.0741.

4-phenoxy-*N*-[(2*Z*)-2-thietanylidene]aniline (6)

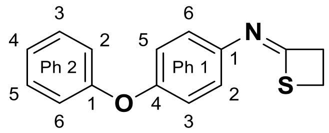

By following the general procedure, starting from 4-phenoxyphenyl isothiocyanate (0.227 g, 1.0 mmol, 1.0 equiv), bromoiodomethane (0.662 g, 0.22 mL, 3.0 mmol, 3.0 equiv) and MeLi-LiBr (2.2 M in Et_2O, 1.27 mL, 2.8 mmol, 2.8 equiv) in dry THF (3 mL), compound **6** was obtained in 70% yield (0.178 g) as a brown oil after purification by column chromatography on silica gel (*n*-hexane:EtOAc 8:2).

^{1}H NMR (400 MHz, C_6D_6) δ: 7.15 (m, 2H, Ph 1 H-2,6), 7.03 (m, 2H, Ph 2 H-3,5), 6.93 (m, 4H, Ph 1 H-3,5 and Ph 2 H-2,6), 6.84 (m, 1H, Ph 2 H-4) 3.28 (m, 2H, CH_2), 2.35 (m, 2H, SCH_2).

^{13}C NMR (100 MHz, C_6D_6) δ: 159.7 (C=N), 158.1 (Ph 2 C-1), 155.0 (Ph 2 C-4), 143.9 (Ph 1 C-1), 130.0 (Ph 2 C-3,5), 123.3 (Ph 2 C-4), 122.8 (Ph 1 C-2,6), 120.1 (Ph 1 C-3,5), 119.1 (Ph 2 C-2,6), 46.9 (CH_2), 20.3 (SCH_2).

HRMS (ESI), m/z: calcd. for $C_{15}H_{14}NOS^+$: 256.0791 $[M + H]^+$; found: 256.0786.

_N_-[(2_Z_)-2-thietanylidene]-4-(trifluoromethoxy)aniline (7)

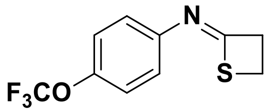

By following the general procedure, starting from 4-(trifluoromethoxy)phenyl isothiocyanate (0.219 g, 1.0 mmol, 1.0 equiv), bromoiodomethane (0.662 g, 0.22 mL, 3.0 mmol, 3.0 equiv) and MeLi-LiBr (2.2 M in Et_2O, 1.27 mL, 2.8 mmol, 2.8 equiv) in dry THF (3 mL), compound **7** was obtained in 73% yield (0.181 g) as a brown oil after purification by column chromatography on silica gel (*n*-hexane:EtOAc 7:3).

^{1}H NMR (400 MHz, C_6D_6) δ: 6.96 (m, 2H, Ph H-2,6), 6.90 (m, 2H, Ph H-3,5), 3.20 (m, 2H, CH_2), 2.29 (m, 2H, SCH_2).

^{13}C NMR (100 MHz, C_6D_6) δ: 162.0 (C=N), 147.0 (Ph C-1), 146.4 (q, $^3J_{C,F}$ = 1.9 Hz, Ph C-4), 122.5 (Ph C-2,6), 122.3 (q, $^4J_{C,F}$ = 0.9 Hz, Ph C-3,5), 121.3 (q, $^1J_{C,F}$ = 256.5 Hz, OCF_3), 46.7 (CH_2), 20.1 (SCH_2).

^{15}N NMR (40 MHz, C_6D_6) δ: -83.5 (C=N).

^{19}F NMR (376 MHz, C_6D_6) δ: -57.8 (s, OCF_3).

HRMS (ESI), m/z: calcd. for $C_{10}H_9F_3NOS^+$: 248.0351 [M + H]+; found: 148.0350.

***N*-[(2*Z*)-2-thietanylidene]-1,3-benzodioxol-5-amine (8)**

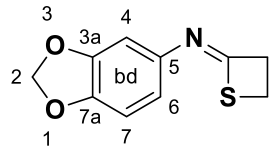

By following the general procedure, starting from 3,4-methylenedioxyphenyl isothiocyanate (0.179 g, 1.0 mmol, 1.0 equiv), bromoiodomethane (0.662 g, 0.22 mL, 3.0 mmol, 3.0 equiv) and MeLi-LiBr (2.2 M in Et_2O, 1.27 mL, 2.8 mmol, 2.8 equiv) in dry THF (3 mL), compound **8** was obtained in 73% yield (0.151 g) as a yellow oil after purification by column chromatography on silica gel (*n*-hexane:EtOAc 8:2).

^{1}H NMR (400 MHz, C_6D_6) δ: 6.90 (d, ^4J = 2.0 Hz, 1H, bd H-4), 6.70 (dd, ^3J = 8.2 Hz, ^4J = 2.0 Hz, 1H, bd H-6), 6.63 (d, ^3J = 8.2 Hz, 1H, bd H-7), 5.25 (s, 2H, bd H-2), 3.24 (m, 2H, CH_2), 2.33 (m, 2H, SCH_2).

^{13}C NMR (100 MHz, C_6D_6) δ: 159.2 (C=N), 148.9 (bd C-3a), 145.5 (bd C-7a), 143.0 (bd C-5), 114.4 (bd C-6), 108.8 (bd C-7), 103.0 (bd C-4), 101.2 (bd C-2), 46.8 (CH_2), 20.4 (SCH_2).

^{15}N NMR (40 MHz, C_6D_6) δ: -81.4 (C=N).

HRMS (ESI), *m/z*: calcd. for $C_{10}H_{10}NO_2S^+$: 208.0427 [M + H]$^+$; found: 208.0431.

4-(methylsulfanyl)-*N*-[(2*Z*)-2-thietanylidene]aniline (9)

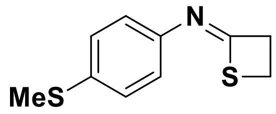

By following the general procedure, starting from 4-(methylthio)phenyl isothiocyanate (0.181 g, 1.0 mmol, 1.0 equiv), bromoiodomethane (0.662 g, 0.22 mL, 3.0 mmol, 3.0 equiv) and MeLi-LiBr (2.2 M in Et_2O, 1.27 mL, 2.8 mmol, 2.8 equiv) in dry THF (3 mL), compound **9** was obtained in 70% yield (0.147 g) as a brown oil after purification by column chromatography on silica gel (*n*-hexane:EtOAc 8:2).

^{1}H NMR (400 MHz, C_6D_6) δ: 7.12 (m, 2H, Ph H-3,5), 7.11 (m, 2H, Ph H-2,6), 3.27 (m, 2H, CH_2), 2.34 (m, 2H, SCH_2), 1.96 (s, 3H, SCH_3).

^{13}C NMR (100 MHz, C_6D_6) δ: 160.1 (C=N), 145.8 (Ph C-1), 135.4 (Ph C-4), 128.4 (Ph C-3,5), 122.0 (Ph C-2,6), 46.9 (CH_2), 20.3 (SCH_2), 16.0 (SCH_3).

HRMS (ESI), *m/z*: calcd. for $C_{10}H_{12}NS_2^+$: 210.0406 $[M + H]^+$; found: 210.0405.

4-(phenylselanyl)-*N*-[(2*Z*)-2-thietanylidene]aniline (10)

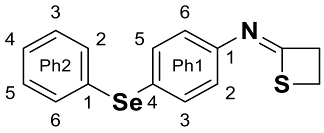

Preparation of (4-isothiocyanatophenyl)(phenyl)selane: 4-(Phenylselanyl)aniline (0.500 g, 2.0 mmol, 1.0 equiv) was dissolved in DCM (20 mL). Thiophosgene (0.463 g, 0.31 mL, 4.0 mmol, 2.0 equiv) was added dropwise at 0 °C. After the addition was complete the reaction was allowed to reach room temperature and stirred for 3 hours. The reaction mixture was diluted with DCM, washed with sat. aqueous $NaHCO_3$, brine and dried over Na_2SO_4. Evaporation of the solvent gave (4-isothiocyanatophenyl)(phenyl)selane as an orange oil in 96% yield (0.557 g) which was used without any further purification. ^{1}H-NMR (200 MHz, $CDCl_3$) δ: 7.51 (m, 2H), 7.39-7.31 (m, 5H), 7.09 (m, 2H). ^{13}C-NMR (50 MHz, $CDCl_3$) δ: 134.0, 133.2, 131.5, 130.2, 130.0, 129.7, 128.8, 126.6.

By following the general procedure, starting from (4-isothiocyanatophenyl)(phenyl)selane (0.290 g, 1.0 mmol, 1.0 equiv), bromoiodomethane (0.662 g, 0.22 mL, 3.0 mmol, 3.0 equiv) and MeLi-LiBr (2.2 M in Et_2O, 1.27 mL, 2.8 mmol, 2.8 equiv) in dry THF (3 mL), compound **10** was obtained in 71% yield (0.226 g) as a yellow oil after purification by column chromatography on silica gel (*n*-hexane:EtOAc 8:2).

^{1}H NMR (400 MHz, C_6D_6) δ: 7.43 (m, 2H, Ph 1 H-3,5), 7.39 (m, 2H, Ph 2 H-2,6), 7.04 (m, 2H, Ph 1 H-2,6) 6.91 (m, 3H, Ph 2 H-3,4,5), 3.21 (m, 2H, CH_2), 2.29 (m, 2H, SCH_2).

^{13}C NMR (100 MHz, C_6D_6) δ: 161.2 (C=N), 148.0 (Ph 1 C-1), 135.0 (Ph 1 C-3,5), 132.8 (Ph 2 C-2,6), 132.4 (Ph 2 C-1), 129.6 (Ph 2 C-3,5), 127.2 (Ph 1 C-4, Ph 2 C-4), 122.4 (Ph 1 C-2,6), 46.8 (CH_2), 20.1 (SCH_2).

^{15}N NMR (40 MHz, C_6D_6) δ: -81.7 (C=N).

^{77}Se NMR (76 MHz) δ: 410.5 (s).

HRMS (ESI), *m/z*: calcd. for $C_{15}H_{14}NSSe^+$: 320.0007 [M + H]$^+$; found: 320.0007.

N-[(2Z)-2-thietanylidene]aniline (11)

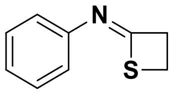

By following the general procedure, starting from phenyl isothiocyanate (0.135 g, 1.0 mmol, 1.0 equiv), bromoiodomethane (0.662 g, 0.22 mL, 3.0 mmol, 3.0 equiv) and MeLi-LiBr (2.2 M in Et_2O, 1.27 mL, 2.8 mmol, 2.8 equiv) in dry THF (3 mL), compound **11** was obtained in 76% yield (0.124 g) as a brown oil after purification by column chromatography on silica gel (*n*-hexane:EtOAc 8:2).

^{1}H NMR (400 MHz, C_6D_6) δ: 7.23 (m, 2H, Ph H-2,6), 7.17 (m, 2H, Ph H-3,5), 6.94 (m, 1H, Ph H-4), 3.26 (m, 2H, CH_2), 2.32 (m, 2H, SCH_2).

^{13}C NMR (100 MHz, C_6D_6) δ: 160.4 (C=N), 148.6 (Ph C-1), 129.6 (Ph C-3,5), 125.3 (Ph C-4) 121.3 (Ph C-2,6), 46.8 (CH_2), 20.0 (SCH_2).

HRMS (ESI), *m/z*: calcd. for $C_9H_{10}NS^+$: 164.0528 $[M + H]^+$; found: 164.0530.

4-fluoro-*N*-[(2*Z*)-2-thietanylidene]aniline (12)

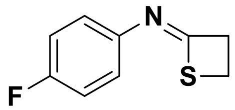

By following the general procedure, starting from 4-fluorophenyl isothiocyanate (0.153 g, 1.0 mmol, 1.0 equiv), bromoiodomethane (0.662 g, 0.22 mL, 3.0 mmol, 3.0 equiv) and MeLi-LiBr (2.2 M in Et_2O, 1.27 mL, 2.8 mmol, 2.8 equiv) in dry THF (3 mL), compound **12** was obtained in 80% yield (0.145 g) as a brown oil after purification by column chromatography on silica gel (*n*-hexane:EtOAc 8:2).

^{1}H NMR (400 MHz, C_6D_6) δ: 7.00 (m, 2H, Ph H-2,6), 6.78 (m, 2H, Ph H-3,5), 3.23 (m, 2H, CH_2), 2.32 (m, 2H, SCH_2).

^{13}C NMR (100 MHz, C_6D_6) δ: 160.6 (d, $^6J_{C,F}$ = 1.8 Hz, C=N), 160.6 (d, $^1J_{C,F}$ = 243.4 Hz, Ph C-4), 122.8 (d, $^3J_{C,F}$ = 8.1 Hz, Ph C-2,6), 116.2 (d, $^2J_{C,F}$ = 22.5 Hz, Ph C-3,5), 46.8 (CH_2), 20.1 (SCH_2).

^{15}N NMR (40 MHz, C_6D_6) δ: -82.5 (C=N).

^{19}F NMR (376 MHz, C_6D_6) δ: -117.9 (m).

HRMS (ESI), *m/z*: calcd. for $C_9H_9FNS^+$: 182.0434 $[M + H]^+$; found: 182.0434.

4-bromo-*N*-[(2*Z*)-2-thietanylidene]aniline (13)

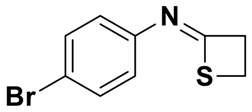

By following the general procedure, starting from 4-bromophenyl isothiocyanate (0.214 g, 1.0 mmol, 1.0 equiv), bromoiodomethane (0.662 g, 0.22 mL, 3.0 mmol, 3.0 equiv) and MeLi-LiBr (2.2 M in Et_2O, 1.27 mL, 2.8 mmol, 2.8 equiv) in dry THF (3 mL), compound **13** was obtained in 78% yield (0.189 g) as a brown oil after purification by column chromatography on silica gel (*n*-hexane:EtOAc 8:2).

^{1}H NMR (400 MHz, C_6D_6) δ: 7.22 (m, 2H, Ph H-3,5), 6.86 (m, 2H, Ph H-2,6), 3.19 (m, 2H, CH_2), 2.29 (m, 2H, SCH_2).

^{13}C NMR (100 MHz, C_6D_6) δ: 161.6 (C=N), 147.4 (Ph C-1), 132.7 (Ph C-3,5), 123.0 (Ph C-2,6) 118.4 (Ph C-4), 46.7 (CH_2), 20.1 (SCH_2).

HRMS (ESI), m/z: calcd. for $C_9H_9BrNS^+$: 241.9634 [M + H]+; found: 241.9632.

4-chloro-*N*-[(2*Z*)-2-thietanylidene]aniline (14)

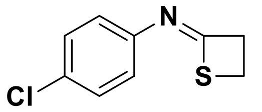

By following the general procedure, starting from 4-chlorophenyl isothiocyanate (0.169 g, 1.0 mmol, 1.0 equiv), bromoiodomethane (0.662 g, 0.22 mL, 3.0 mmol, 3.0 equiv) and MeLi-LiBr (2.2 M in Et_2O, 1.27 mL, 2.8 mmol, 2.8 equiv) in dry THF (3 mL), compound **14** was obtained in 71% yield (0.140 g) as a brown oil after purification by column chromatography on silica gel (*n*-hexane:EtOAc 8:2).

^{1}H NMR (400 MHz, C_6D_6) δ: 7.07 (m, 2H, Ph H-3,5), 6.93 (m, 2H, Ph H-2,6), 3.21 (m, 2H, CH_2), 2.29 (m, 2H, SCH_2).

^{13}C NMR (100 MHz, C_6D_6) δ: 161.6 (C=N), 146.9 (Ph C-1), 130.6 (Ph C-4), 129.7 (Ph C-3,5), 122.6 (Ph C-2,6), 46.7 (CH_2), 20.1 (SCH_2).

^{15}N NMR (40 MHz, C_6D_6) δ: -82.3 (C=N).

HRMS (ESI), m/z: calcd. for $C_9H_9ClNS^+$: 198.0139 [M + H]+; found: 198.0139.

3-chloro-*N*-[(2*Z*)-2-thietanylidene]aniline (15)

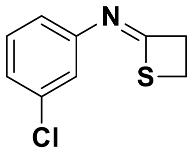

By following the general procedure, starting from 3-chlorophenyl isothiocyanate (0.169 g, 1.0 mmol, 1.0 equiv), bromoiodomethane (0.662 g, 0.22 mL, 3.0 mmol, 3.0 equiv) and MeLi-LiBr (2.2 M in Et_2O, 1.27 mL, 2.8 mmol, 2.8 equiv) in dry THF (3 mL), compound **15** was obtained in 74% yield (0.146 g) as a brown oil after purification by column chromatography on silica gel (*n*-hexane:EtOAc 8:2).

^{1}H NMR (400 MHz, C_6D_6) δ: 7.27 (t, *J* = 2.0 Hz, 1H, Ph H-2), 6.93 (m, 1H, Ph H-6), 6.90 (m, 1H, Ph H-4), 6.83 (m, 1H, Ph H-5), 3.16 (m, 2H, CH_2), 2.25 (m, 2H, SCH_2).

^{13}C NMR (100 MHz, C_6D_6) δ: 162.7 (C=N), 149.9 (Ph C-1), 135.3 (Ph C-3), 130.7 (Ph C-5), 125.2 (Ph C-4), 121.5 (Ph C-2), 119.4 (Ph C-6), 46.5 (CH_2), 20.0 (SCH_2).

^{15}N NMR (40 MHz, C_6D_6) δ: -81.7 (C=N).

HRMS (ESI), m/z: calcd. for $C_9H_9ClNS^+$: 198.0139 $[M + H]^+$; found: 198.0137.

3,5-dichloro-*N*-[(2*Z*)-2-thietanylidene]aniline (16)

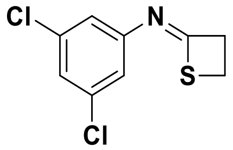

By following the general procedure, starting from 3,5-dichlorophenyl isothiocyanate (0.204 g, 1.0 mmol, 1.0 equiv), bromoiodomethane (0.662 g, 0.22 mL, 3.0 mmol, 3.0 equiv) and MeLi-LiBr (2.2 M in Et_2O, 1.27 mL, 2.8 mmol, 2.8 equiv) in dry THF (3 mL), compound **16** was obtained in 82% yield (0.190 g) as a yellow solid (mp: 55-58 °C) after purification by column chromatography on silica gel (*n*-hexane:DCM 3:7).

^{1}H NMR (400 MHz, C_6D_6) δ: 7.00 (d, ^4J = 1.9 Hz, 2H, Ph H-2,6), 6.90 (t, ^4J = 1.9 Hz, 1H, Ph H-4), 3.08 (m, 2H, CH_2), 2.19 (m, 2H, SCH_2).

^{13}C NMR (100 MHz, C_6D_6) δ: 164.6 (C=N), 150.5 (Ph C-1), 135.9 (Ph C-3,5), 125.1 (Ph C-4), 119.8 (Ph C-2,6), 46.3 (CH_2), 20.0 (SCH_2).

^{15}N NMR (40 MHz, C_6D_6) δ: -84.8 (C=N).

HRMS (ESI), *m/z*: calcd. for $C_9H_8Cl_2NS^+$: 231.9749 $[M + H]^+$; found: 231.9739.

3-chloro-4-fluoro-*N*-[(2*Z*)-2-thietanylidene]aniline (17)

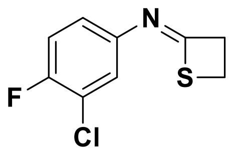

By following the general procedure, starting from 3-chloro-4-fluorophenyl isothiocyanate (0.188 g, 1.0 mmol, 1.0 equiv), bromoiodomethane (0.662 g, 0.22 mL, 3.0 mmol, 3.0 equiv) and MeLi-LiBr (2.2 M in Et_2O, 1.27 mL, 2.8 mmol, 2.8 equiv) in dry THF (3 mL), compound **17** was obtained in 75% yield (0.162 g) as a brown oil after purification by column chromatography on silica gel (*n*-hexane:EtOAc 8:2).

^{1}H NMR (400 MHz, C_6D_6) δ: 7.17 (m, 1H, Ph H-2), 6.73 (ddd, ^3J = 8.7 Hz, ^4J = 4.3 Hz, ^4J = 2.6 Hz, 1H, Ph H-6), 6.61 (t, ^3J = 8.7 Hz, 1H, Ph H-5) 3.16 (m, 2H, CH_2), 2.27 (m, 2H, SCH_2).

^{13}C NMR (100 MHz, C_6D_6) δ: 162.6 (C=N), 155.9 (d, $^1J_{C,F}$ = 246.3 Hz, Ph C-4), 145.1 (d, $^4J_{C,F}$ = 3.4 Hz, Ph C-1), 123.2 (Ph C-2), 121.8 (d, $^2J_{C,F}$ = 18.7 Hz, Ph C-3), 120.9 (d, $^3J_{C,F}$ = 6.9 Hz, Ph C-6), 117.3 (d, $^2J_{C,F}$ = 22.0 Hz, Ph C-5), 46.6 (CH_2), 20.1 (SCH_2).

^{19}F NMR (376 MHz, C_6D_6) δ: -120.3 (m).

HRMS (ESI), m/z: calcd. for $C_9H_8ClFNS^+$: 216.0045 $[M + H]^+$; found: 216.0046.

***N*-[(2*Z*)-2-thietanylidene]-4-(trifluoromethyl)aniline (18)**

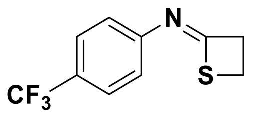

By following the general procedure, starting from 4-trifluoromethylphenyl isothiocyanate (0.203 g, 1.0 mmol, 1.0 equiv), bromoiodomethane (0.662 g, 0.22 mL, 3.0 mmol, 3.0 equiv) and MeLi-LiBr (2.2 M in Et_2O, 1.27 mL, 2.8 mmol, 2.8 equiv) in dry THF (3 mL), compound **18** was obtained in 72% yield (0.166 g) as a brown oil after purification by column chromatography on silica gel (*n*-hexane:EtOAc 8:2).

^{1}H NMR (400 MHz, C_6D_6) δ: 7.31 (m, 2H, Ph H-3,5), 6.98 (m, 2H, Ph H-2,6), 3.18 (m, 2H, CH_2), 2.27 (m, 2H, SCH_2).

^{13}C NMR (100 MHz, C_6D_6) δ: 163.2 (C=N), 151.5 (Ph C-1), 128.3 (Ph C-4), 126.9 (q, $^3J_{C,F}$ = 3.8 Hz, Ph C-3,5), 125.1 (q, $^1J_{C,F}$ = 271.6 Hz, CF_3), 121.3 (Ph C-2,6), 46.6 (CH_2), 19.9 (SCH_2).

^{15}N NMR (40 MHz, C_6D_6) δ: -81.8 (C=N).

^{19}F NMR (376 MHz, C_6D_6) δ: -61.7 (s, CF_3).

HRMS (ESI), m/z: calcd. for $C_{10}H_9F_3NS^+$: 232.0402 $[M + H]^+$; found: 232.0407.

4-methyl-*N*-[(2*Z*)-2-thietanylidene]aniline (19)

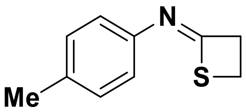

By following the general procedure, starting from *p*-tolyl isothiocyanate (0.149 g, 1.0 mmol, 1.0 equiv), bromoiodomethane (0.662 g, 0.22 mL, 3.0 mmol, 3.0 equiv) and MeLi-LiBr (2.2 M in Et_2O, 1.27 mL, 2.8 mmol, 2.8 equiv) in dry THF (3 mL), compound **19** was obtained in 76% yield (0.135 g) as an brown oil after purification by column chromatography on silica gel (*n*-hexane:EtOAc 8:2).

^{1}H NMR (400 MHz, C_6D_6) δ: 7.20 (m, 2H, Ph H-2,6), 6.99 (m, 2H, Ph H-3,5), 3.30 (m, 2H, CH_2), 2.35 (m, 2H, SCH_2), 2.07 (s, 3H, CH_3).

^{13}C NMR (100 MHz, C_6D_6) δ: 159.4 (C=N), 146.1 (Ph C-1), 134.6 (Ph C-4), 130.2 (Ph C-3,5), 121.3 (Ph C-2,6), 46.9 (CH_2), 20.9 (CH_3), 20.2 (SCH_2).

HRMS (ESI), *m/z*: calcd. for $C_{10}H_{12}NS^+$: 178.0685 $[M + H]^+$; found: 178.0688.

3-methyl-*N*-[(2*Z*)-2-thietanylidene]aniline (20)

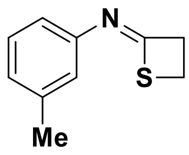

By following the general procedure, starting from 3-tolyl isothiocyanate (0.149 g, 1.0 mmol, 1.0 equiv), bromoiodomethane (0.662 g, 0.22 mL, 3.0 mmol, 3.0 equiv) and MeLi-LiBr (2.2 M in Et_2O, 1.27 mL, 2.8 mmol, 2.8 equiv) in dry THF (3 mL), compound **20** was obtained in 79% yield (0.140 g) as a brown oil after purification by column chromatography on silica gel (*n*-hexane:EtOAc 9:1).

^{1}H NMR (400 MHz, C_6D_6) δ: 7.13 (m, 1H, Ph H-5), 7.11 (m, 1H, Ph H-6), 7.08 (m, 1H, Ph H-2), 6.80 (m, 1H, Ph H-4), 3.30 (m, 2H, CH_2), 2.35 (m, 2H, SCH_2), 2.10 (s, 3H, CH_3).

^{13}C NMR (100 MHz, C_6D_6) δ: 160.0 (C=N), 148.7 (Ph C-1), 139.3 (Ph C-3), 129.5 (Ph C-5), 126.1 (Ph C-4), 122.1 (Ph C-2), 118.2 (Ph C-6), 46.8 (CH_2), 21.4 (CH_3), 20.0 (SCH_2).

HRMS (ESI), *m/z*: calcd. for $C_{10}H_{12}NS^+$: 178.0685 [M + H]$^+$; found: 178.0692.

3,4-dimethyl-*N*-[(2*Z*)-2-thietanylidene]aniline (21)

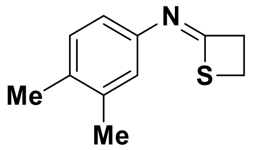

By following the general procedure, starting from 3,4-dimethylphenyl isothiocyanate (0.163 g, 1.0 mmol, 1.0 equiv), bromoiodomethane (0.662 g, 0.22 mL, 3.0 mmol, 3.0 equiv) and MeLi-LiBr (2.2 M in Et_2O, 1.27 mL, 2.8 mmol, 2.8 equiv) in dry THF (3 mL), compound **21** was obtained in 73% yield (0.140 g) as a brown oil after purification by column chromatography on silica gel (*n*-hexane:EtOAc 9:1).

^{1}H NMR (400 MHz, C_6D_6) δ: 7.11 (m, 1H, Ph H-2), 7.09 (m, 1H, Ph H-6), 7.00 (m, 1H, Ph H-5), 3.33 (m, 2H, CH_2) 2.38 (m, 2H, SCH_2) 2.00 (s, 3H, 3-CH_3), 1.97 (s, 3H, 4-CH_3).

^{13}C NMR (100 MHz, C_6D_6) δ: 159.1 (C=N), 146.5 (Ph C-1), 137.6 (Ph C-3), 133.3 (Ph C-4), 130.7 (Ph C-5), 122.8 (Ph C-2), 118.6 (Ph C-6), 46.5 (CH_2), 20.2 (SCH_2), 19.9 (3-CH_3), 19.2 (4-CH_3).

HRMS (ESI), *m/z*: calcd. for $C_{11}H_{14}NS^+$: 192.0841 $[M + H]^+$; found: 192.0845.

4-isopropyl-*N*-[(2*Z*)-2-thietanylidene]aniline (22)

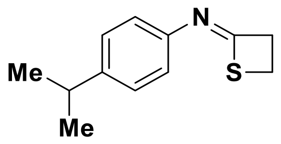

By following the general procedure, starting from 4-isopropylphenyl isothiocyanate (0.177 g, 1.0 mmol, 1.0 equiv), bromoiodomethane (0.662 g, 0.22 mL, 3.0 mmol, 3.0 equiv) and MeLi-LiBr (2.2 M in Et_2O, 1.27 mL, 2.8 mmol, 2.8 equiv) in dry THF (3 mL), compound **22** was obtained in 71% yield (0.146 g) as a brown oil after purification by column chromatography on silica gel (*n*-hexane:EtOAc 9:1).

^{1}H NMR (400 MHz, C_6D_6) δ: 7.26 (m, 2H, Ph H-2,6), 7.09 (m, 2H, Ph H-3,5), 3.30 (m, 2H, CH_2), 2.67 (sept, ^3J = 6.9 Hz, 1H, CH), 2.36 (m, 2H, SCH_2), 1.10 (d, ^3J = 6.9 Hz, 6H, CH_3).

^{13}C NMR (100 MHz, C_6D_6) δ: 159.3 (C=N), 146.4 (Ph C-1), 145.7 (Ph C-4), 127.6 (Ph C-3,5), 121.4 (Ph C-2,6), 46.9 (CH_2), 34.0 (CH), 24.2 (CH_3), 20.2 (SCH_2).

^{15}N NMR (40 MHz, C_6D_6) δ: -80.1 (C=N).

HRMS (ESI), *m/z*: calcd. for $C_{12}H_{16}NS^+$: 206.0998 $[M + H]^+$; found: 206.1005.

N-[(2Z)-2-thietanylidene]-5-indanamine (23)

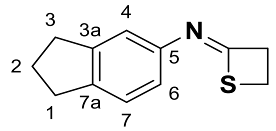

By following the general procedure, starting from 5-indanyl isothiocyanate (0.175 g, 1.0 mmol, 1.0 equiv), bromoiodomethane (0.662 g, 0.22 mL, 3.0 mmol, 3.0 equiv) and MeLi-LiBr (2.2 M in Et_2O, 1.27 mL, 2.8 mmol, 2.8 equiv) in dry THF (3 mL), compound **23** was obtained in 77% yield (0.157 g) as a brown oil after purification by column chromatography on silica gel (*n*-hexane:EtOAc 9:1).

^{1}H NMR (400 MHz, C_6D_6) δ: 7.19 (s, 1H, indanyl H-4), 7.14 (m, 1H, indanyl H-6), 7.10 (m, 1H, indanyl H-7), 3.33 (m, 2H, CH_2), 2.66 (m, 2H, indanyl H-3), 2.63 (m, 2H, indanyl H-1), 2.38 (m, 2H, SCH_2), 1.76 (m, 2H, indanyl H-2).

^{13}C NMR (100 MHz, C_6D_6) δ: 158.9 (C=N), 147.1 (indanyl C-5), 145.6 (indanyl C-3a), 141.0 (indanyl C-7a), 125.2 (indanyl C-7), 119.4 (indanyl C-6), 117.3 (indanyl C-4), 46.9 (CH_2), 33.2 (indanyl C-3), 32.7 (indanyl C-1), 25.9 (indanyl C-2), 20.2 (SCH_2).

^{15}N NMR (40 MHz, C_6D_6) δ: -79.1 (C=N).

HRMS (ESI), *m/z*: calcd. for $C_{12}H_{14}NS^+$: 204.0841 $[M + H]^+$; found: 204.0845.

***N*-[(2*Z*)-2-thietanylidene]-4-vinylaniline (24)**

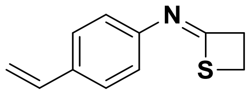

By following the general procedure, starting from 4-vinylphenyl isothiocyanate[1] (0.161 g, 1.0 mmol, 1.0 equiv), bromoiodomethane (0.662 g, 0.22 mL, 3.0 mmol, 3.0 equiv) and MeLi-LiBr (2.2 M in Et_2O, 1.27 mL, 2.8 mmol, 2.8 equiv) in dry THF (3 mL), compound **24** was obtained in 70% yield (0.132 g) as a brown oil after purification by column chromatography on silica gel (*n*-hexane:EtOAc 8:2).

^{1}H NMR (400 MHz, C_6D_6) δ: 7.23 (m, 2H, Ph H-3,5), 7.17 (m, 2H, Ph H-2,6), 6.54 (dd, ^3J = 17.6, 10.9 Hz, 1H, C<u>H</u>=CH_2), 5.54 (dd, ^3J = 17.6 Hz, ^2J = 1.0 Hz, 1H, CH=C<u>H</u>$_2$ trans), 5.04 (dd, ^3J = 10.9 Hz, ^2J = 1.0 Hz, 1H, CH=C<u>H</u>$_2$ cis), 3.28 (m, 2H, CH_2), 2.34 (m, 2H, SCH_2).

^{13}C NMR (100 MHz, C_6D_6) δ: 160.5 (C=N), 148.0 (Ph C-1), 136.9 (<u>C</u>H=CH_2), 134.9 (Ph C-4), 127.7 (Ph C-3,5), 121.5 (Ph C-2,6), 113.0 (CH=<u>C</u>H_2), 46.8 (CH_2), 20.2 (SCH_2).

^{15}N NMR (40 MHz, C_6D_6) δ: -80.6 (C=N).

HRMS (ESI), *m/z*: calcd. for $C_{11}H_{12}NS^+$: 190.0685 $[M + H]^+$; found: 190.0684.

3-ethynyl-*N*-[(2*Z*)-2-thietanylidene]aniline (25)

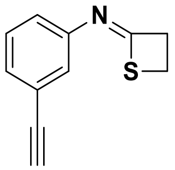

By following the general procedure, starting from 3-ethynylphenyl isothiocyanate[2] (0.159 g, 1.0 mmol, 1.0 equiv), bromoiodomethane (0.662 g, 0.22 mL, 3.0 mmol, 3.0 equiv) and MeLi-LiBr (2.2 M in Et_2O, 1.27 mL, 2.8 mmol, 2.8 equiv) in dry THF (3 mL), compound **25** was obtained in 74% yield (0.138 g) as a yellow oil after purification by column chromatography on silica gel (*n*-hexane:Et_2O 7:3).

^{1}H NMR (400 MHz, C_6D_6) δ: 7.50 (m, 1H, Ph H-2), 7.20 (m, 1H, Ph H-4), 7.07 (m, 1H, Ph H-6), 6.93 (m, 1H, Ph H-5), 3.18 (m, 2H, CH_2), 2.70 (s, 1H, C≡CH), 2.26 (m, 2H, SCH_2).

^{13}C NMR (100 MHz, C_6D_6) δ: 162.0 (C=N), 148.7 (Ph C-1), 129.7 (Ph C-5), 129.0 (Ph C-4), 124.8 (Ph C-2), 123.9 (Ph C-3), 121.9 (Ph C-6), 83.7 ($\underline{C}$≡CH), 78.1 (C≡$\underline{C}$H), 46.6 (CH_2), 20.0 (SCH_2).

^{15}N NMR (40 MHz, C_6D_6) δ: -81.8 (C=N).

HRMS (ESI), *m/z*: calcd. for $C_{11}H_{10}NS^+$: 188.0528 $[M + H]^+$; found: 188.0525.

2-methyl-2-propanyl-4-[(2Z)-2-thietanylideneamino]benzoate (26)

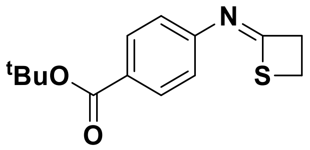

By following the general procedure, starting from *tert*-butyl 4-isothiocyanatobenzoate (0.235 g, 1.0 mmol, 1.0 equiv), bromoiodomethane (0.662 g, 0.22 mL, 3.0 mmol, 3.0 equiv) and MeLi-LiBr (2.2 M in Et_2O, 1.27 mL, 2.8 mmol, 2.8 equiv) in dry THF (3 mL), compound **26** was obtained in 80% yield (0.211 g) as a yellowish solid (mp: 52-55 °C) after purification by column chromatography on silica gel (*n*-hexane:EtOAc 6:4).

^{1}H NMR (400 MHz, C_6D_6) δ: 8.17 (m, 2H, Ph H-2,6), 7.12 (m, 2H, Ph H-3,5), 3.20 (m, 2H, CH_2), 2.27 (m, 2H, SCH_2), 1.47 (s, 9H, CCH_3).

^{13}C NMR (100 MHz, C_6D_6) δ: 165.3 (C=O), 162.6 (C=N), 152.3 (Ph C-4), 131.5 (Ph C-2,6), 129.2 (Ph C-1), 120.8 (Ph C-3,5), 80.2 ($\underline{C}CH_3$), 46.6 (CH_2), 28.2 ($C\underline{C}H_3$), 19.9 (SCH_2).

^{15}N NMR (40 MHz, C_6D_6) δ: -80.4 (C=N).

HRMS (ESI), *m/z*: calcd. for $C_{14}H_{17}NNaO_2S^+$: 286.0872 [M + Na]$^+$; found: 286.0868.

3-[(2*Z*)-2-thietanylideneamino]benzonitrile (27)

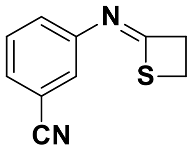

By following the general procedure, starting from 3-cyanophenyl isothiocyanate (0.160 g, 1.0 mmol, 1.0 equiv), bromoiodomethane (0.662 g, 0.22 mL, 3.0 mmol, 3.0 equiv) and MeLi-LiBr (2.2 M in Et_2O, 1.27 mL, 2.8 mmol, 2.8 equiv) in dry THF (3 mL), compound **27** was obtained in 69% yield (0.129 g) as a brown solid (mp: 122-125 °C) after purification by column chromatography on silica gel (*n*-hexane:EtOAc 7:3).

^{1}H NMR (400 MHz, C_6D_6) δ: 7.16 (m, 1H, Ph H-2), 6.97 (m, 1H, Ph H-6), 6.78 (m, 1H, Ph H-4), 6.68 (m, 1H, Ph H-5), 3.13 (m, 2H, CH_2), 2.23 (m, 2H, SCH_2).

^{13}C NMR (100 MHz, C_6D_6) δ: 163.9 (C=N), 149.0 (Ph C-3), 130.2 (Ph C-5), 128.3 (Ph C-6), 125.0 (Ph C-4), 124.3 (Ph C-2), 118.6 (C≡N), 114.0 (Ph C-1), 46.4 (CH_2), 19.9 (SCH_2).

HRMS (ESI), m/z: calcd. for $C_{10}H_9N_2S^+$: 189.0481 [M + H]$^+$; found: 189.0478.

***N,N*-dimethyl-*N´*-[(2*Z*)-2-thietanylidene]-1,4-benzenediamine (28)**

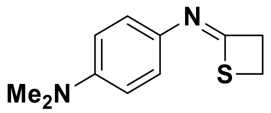

By following the general procedure, starting from 4-(*N,N*-dimethylamino)phenyl isothiocyanate (0.178 g, 1.0 mmol, 1.0 equiv), bromoiodomethane (0.662 g, 0.22 mL, 3.0 mmol, 3.0 equiv) and MeLi-LiBr (2.2 M in Et_2O, 1.27 mL, 2.8 mmol, 2.8 equiv) in dry THF (3 mL), compound **28** was obtained in 75% yield (0.155 g) as a brown solid (mp: 78-80 °C) after purification by column chromatography on silica gel (*n*-hexane:EtOAc 8:2).

^{1}H NMR (400 MHz, C_6D_6) δ: 7.35 (m, 2H, Ph H-3,5), 6.58 (m, 2H, Ph H-2,6), 3.40 (m, 2H, CH_2), 2.47 (s, 6H, CH_3), 2.45 (m, 2H, SCH_2).

^{13}C NMR (100 MHz, C_6D_6) δ: 155.4 (C=N), 148.8 (Ph C-1), 138.1 (Ph C-4), 122.8 (Ph C-3,5), 113.6 (Ph C-2,6), 47.4 (CH_2), 40.4 (CH_3), 20.7 (SCH_2).

HRMS (ESI), m/z: calcd. for $C_{11}H_{15}N_2S^+$: 207.0950 [M + H]+; found: 207.0953.

4-(4-morpholinyl)-*N*-[(2*Z*)-2-thietanylidene]aniline (29)

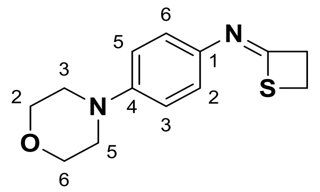

By following the general procedure, starting from 4-morpholinophenyl isothiocyanate (0.220 g, 1.0 mmol, 1.0 equiv), bromoiodomethane (0.662 g, 0.22 mL, 3.0 mmol, 3.0 equiv) and MeLi-LiBr (2.2 M in Et_2O, 1.27 mL, 2.8 mmol, 2.8 equiv) in dry THF (3 mL), compound **29** was obtained in 82% yield (0.195 g) as a yellowish solid (mp: 115-117 °C) after purification by column chromatography on silica gel (*n*-hexane:EtOAc 6:4).

^{1}H NMR (400 MHz, C_6D_6) δ: 7.30 (m, 2H, Ph H-2,6), 6.66 (m, 2H, Ph H-3,5), 3.52 (m, 4H, morph H-2,6), 3.38 (m, 2H, CH_2), 2.69 (m, 4H, morph H-3,5), 2.43 (m, 2H, SCH_2).

^{13}C NMR (100 MHz, C_6D_6) δ: 157.4 (C=N), 149.4 (Ph C-4), 140.8 (Ph C-1), 122.5 (Ph C-2,6), 116.7 (Ph C-3,5), 66.9 (morph C-2,6), 49.6 (morph C-3,5), 47.2 (CH_2), 20.5 (SCH_2).

^{15}N NMR (40 MHz, C_6D_6) δ: -318.9 (morph), -81.9 (C=N).

HRMS (ESI), *m/z*: calcd. for $C_{13}H_{17}N_2OS^+$: 249.1056 $[M + H]^+$; found: 249.1064.

4-[(E)-phenyldiazenyl]-*N*-[(2*Z*)-2-thietanylidene]aniline (30)

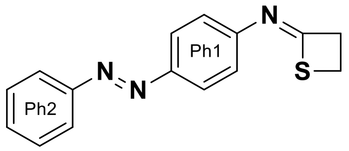

By following the general procedure, starting from 4-(phenylazo)phenyl isothiocyanate (0.239 g, 1.0 mmol, 1.0 equiv), bromoiodomethane (0.662 g, 0.22 mL, 3.0 mmol, 3.0 equiv) and MeLi-LiBr (2.2 M in Et_2O, 1.27 mL, 2.8 mmol, 2.8 equiv) in dry THF (3 mL), compound **30** was obtained in 67% yield (0.179 g) as an orange oil after purification by column chromatography on silica gel (*n*-hexane:EtOAc 8:2).

^{1}H NMR (400 MHz, C_6D_6) δ: 8.07 (m, 2H, Ph 1 H-3,5), 8.03 (m, 2H, Ph 2 H-2,6), 7.25 (m, 2H, Ph 1 H-2,6), 7.19 (m, 2H, Ph 2 H-3,5), 7.10 (m, 1H, Ph 2 H-4), 3.23 (m, 2H, CH_2), 2.30 (m, 2H, SCH_2).

^{13}C NMR (100 MHz, C_6D_6) δ: 162.3 (C=N), 153.4 (Ph2 C-1), 151.0 (Ph 1 C-1), 150.6 (Ph 1 C-4), 130.9 (Ph2 C-4), 129.3 (Ph 2 C-3,5), 124.9 (Ph 1 C-3,5), 123.3 (Ph2 C-2,6), 121.9 (Ph 1 C-2,6), 46.7 (CH_2), 20.1 (SCH_2).

HRMS (ESI), m/z: calcd. for $C_{15}H_{14}N_3S^+$: 268.0903 $[M + H]^+$; found: 268.0902.

4-azido-*N*-[(2*Z*)-2-thietanylidene]aniline (31)

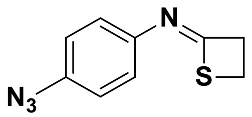

By following the general procedure, starting from 4-azidophenyl isothiocyanate (0.176 g, 1.0 mmol, 1.0 equiv), bromoiodomethane (0.662 g, 0.22 mL, 3.0 mmol, 3.0 equiv) and MeLi-LiBr (2.2 M in Et_2O, 1.27 mL, 2.8 mmol, 2.8 equiv) in dry THF (3 mL), compound **31** was obtained in 69% yield (0.141 g) as a brown oil after purification by column chromatography on silica gel (*n*-hexane:EtOAc 8:2).

^{1}H NMR (400 MHz, C_6D_6) δ: δ: 7.02 (m, 2H, Ph H-2,6), 6.67 (m, 2H, Ph H-3,5), 3.25 (m, 2H, CH_2), 2.34 (m, 2H, SCH_2).

^{13}C NMR (100 MHz, C_6D_6) δ: 160.6 (C=N), 145.3 (Ph C-1), 137.0 (Ph C-4), 122.8 (Ph C-2,6) 120.2 (Ph C-3,5), 46.8 (CH_2), 20.3 (SCH_2).

HRMS (ESI), m/z: calcd. for $C_9H_9N_4S^+$: 205.0542 [M + H]+; found: 205.0541.

4-isothiocyanato-N-[(2Z)-2-thietanylidene]aniline (32)

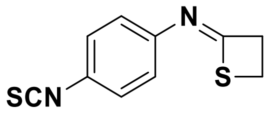

By following the general procedure, starting from 1,4-phenylene diisothiocyanate (0.192 g, 1.0 mmol, 1.0 equiv), bromoiodomethane (0.662 g, 0.22 mL, 3.0 mmol, 3.0 equiv) and MeLi-LiBr (2.2 M in Et_2O, 1.27 mL, 2.8 mmol, 2.8 equiv) in dry THF (3 mL), compound **32** was obtained in 71% yield (0.156 g) as a white solid (mp: 102-104 °C) after purification by column chromatography on silica gel (*n*-hexane:EtOAc 9:1).

^{1}H NMR (400 MHz, C_6D_6) δ: 6.81 (m, 2H, Ph H-2,6), 6.55 (m, 2H, Ph H-3,5), 3.18 (m, 2H, CH_2), 2.29 (m, 2H, SCH_2).

^{13}C NMR (100 MHz, C_6D_6) δ: 162.1 (C=N), 147.1 (Ph C-1), 127.9 (Ph C-4), 126.9 (Ph C-3,5), 122.1 (Ph C-2,6), 46.7 (CH_2), 20.1 (SCH_2).

^{15}N NMR (40 MHz, C_6D_6) δ: -82.9 (C=N).

HRMS (ESI), *m/z*: calcd. for $C_{10}H_9N_2S_2^+$: 221.0202 [M + H]$^+$; found: 221.0198.

***N*-[(2*Z*)-2-thietanylidene]-3-pyridinammine (33)**

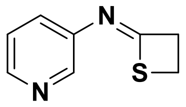

By following the general procedure, starting from pyridine-3-isothiocyanate (0.136 g, 1.0 mmol, 1.0 equiv), bromoiodomethane (0.662 g, 0.22 mL, 3.0 mmol, 3.0 equiv) and MeLi-LiBr (2.2 M in Et_2O, 1.27 mL, 2.8 mmol, 2.8 equiv) in dry THF (3 mL), compound **33** was obtained in 74% yield (0.122 g) as a brown oil after purification by column chromatography on silica gel (*n*-hexane:EtOAc 6:4).

^{1}H NMR (400 MHz, C_6D_6) δ: 8.76 (dd, ^4J = 2.6 Hz, ^5J = 0.8 Hz, 1H, Pyr H-2), 8.34 (dd, ^3J = 4.7 Hz, ^4J = 1.6 Hz, 1H, Pyr H-6), 7.18 (ddd, ^3J = 8.1 Hz, ^4J = 2.6 Hz, ^4J = 1.6 Hz, 1H, Pyr H-4), 6.72 (dd, ^3J = 8.1 Hz, ^3J = 4.7 Hz, ^5J = 0.8 Hz, 1H, Pyr H-5), 3.15 (m, 2H, CH_2), 2.24 (m, 2H, SCH_2).

^{13}C NMR (100 MHz, C_6D_6) δ: 163.5 (C=N), 146.7 (Pyr C-6), 144.3 (Pyr C-3), 143.7 (Pyr C-2), 127.4 (Pyr C-4), 123.8 (Pyr C-5), 46.7 (CH_2), 19.9 (SCH_2).

HRMS (ESI), *m/z*: calcd. for $C_8H_9N_2S^+$: 165.0481 $[M + H]^+$; found: 165.0489.

4-ethoxy-*N*-[(2*Z*)-($^2H_4$)-2-thietanylidene]aniline (34)

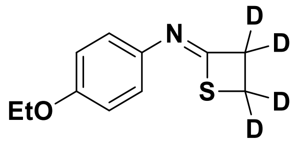

By following the general procedure, starting from 4-ethoxyphenyl isothiocyanate (0.179 g, 1.0 mmol, 1.0 equiv), diiodomethane-d_2 (0.810 g, 0.24 mL, 3.0 mmol, 3.0 equiv) and MeLi-LiBr (2.2 M in Et_2O, 1.27 mL, 2.8 mmol, 2.8 equiv) in dry THF (3 mL), compound **34** was obtained in 71% yield (0.150 g) as a brown solid (mp: 64-66 °C) after purification by column chromatography on silica gel (*n*-hexane:EtOAc 8:2).

^{1}H NMR (400 MHz, C_6D_6) δ: 7.24 (m, 2H, Ph H-2,6), 6.81 (m, 2H, Ph H-3,5), 3.54 (q, ^3J = 7.0 Hz, 2H, OCH_2), 1.09 (t, ^3J = 7.0 Hz, 3H, CH_3).

^{13}C NMR (100 MHz, C_6D_6) δ: 157.9 (C=N), 157.1 (Ph C-4), 141.5 (Ph C-1), 122.7 (Ph C-2,6), 115.4 (Ph C-3,5), 63.4 (OCH_2), 46.3 (quint, $^1J_{C,D}$ = 21.0 Hz, CH_2), 19.6 (quint, $^1J_{C,D}$ = 19.6 Hz, SCH_2), 14.9 (CH_3).

^{15}N NMR (40 MHz, C_6D_6) δ: -81.4 (C=N).

HRMS (ESI), *m/z*: calcd. for $C_{11}H_{10}D_4NOS^+$: 212.1042 $[M + H]^+$; found: 212.1036.

4-(1-piperidinyl)-*N*-[(2*Z*)-2-thietanylidene]aniline (35)

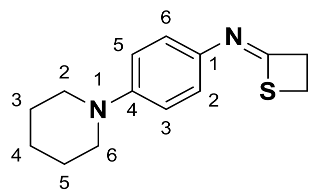

4-bromo-*N*-[(2*Z*)-2-thietanylidene]aniline (0.170 g, 0.7 mmol, 1.0 equiv), dichlorobis(tri-*o*-tolylphosphine)palladium(II) (0.028 g, 5 mol%), and piperidine (0.060 g, 0.07 mL, 0.7 mmol, 1.0 equiv) were dissolved in toluene. A solution of lithium bis(trimethylsilyl)amide (1 M in THF, 0.84 mL, 0.8 mmol, 1.2 equiv) was added and the mixture was heated at 110 °C for 18 hours. The reaction mixture was filtered on celite. The filtrate was washed two times with water, dried over Na_2SO_4 and the solvent was removed under reduced pressure. The residue was purified by column chromatography on silica gel (*n*-hexane:EtOAc 8:2) to give compound **35** in 79% yield (0.136 g) as a brown oil.

^{1}H NMR (400 MHz, C_6D_6) δ: 7.32 (m, 2H, Ph H-2,6), 6.80 (m, 2H, Ph H-3,5), 3.37 (m, 2H, CH_2), 2.86 (m, 4H, piperidine H-2,6), 2.43 (m, 2H, SCH_2), 1.43 (m, 4H, piperidine H-3,5), 1.28 (m, 2H, piperidine H-4).

^{13}C NMR (100 MHz, C_6D_6) δ: 156.6 (C=N), 150.4 (Ph C-4), 140.2 (Ph C-1), 122.5 (Ph C-2,6), 117.6 (Ph C-3,5), 50.9 (piperidine C-2,6), 47.2 (CH_2), 26.1 (piperidine C-3,5), 24.6 (piperidine C-4), 20.5 (SCH_2).

^{15}N NMR (40 MHz, C_6D_6) δ: -314.2 (piperidine), -81.6 (C=N).

HRMS (ESI), *m/z*: calcd. for $C_{14}H_{19}N_2S^+$: 247.1263 [M + H]$^+$; found: 247.1270.

3-[(4-ethoxyphenyl)amino]-1-propanethiol (36)

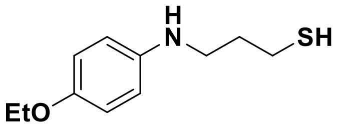

To a solution of 4-ethoxy-*N*-[(2*Z*)-2-thietanylidene]aniline (0.145 g, 0.7 mmol, 1.0 equiv) in dry THF (2 mL) a solution of $LiAlH_4$ (1M in THF, 0.7 mL, 0.7 mmol, 1.0 equiv) was added dropwise at 0 °C. The reaction mixture was allowed to reach room temperature and then stirred for 1.5 hours before being quenched with water. The reaction mixture was extracted exhaustively with Et_2O (3 x 10 mL). The combined organic extracts were washed with brine, dried over Na_2SO_4 and the solvent was removed under reduced pressure. The residue was purified by column chromatography on silica gel (*n*-hexane:EtOAc 8:2) to give compound **36** in 95% yield (0.140 g) as a yellow oil.

^{1}H NMR (400 MHz, $CDCl_3$) δ: 6.78 (m, 2H, Ph H-3,5), 6.59 (m, 2H, Ph H-2,6), 3.96 (q, ^3J = 7.0 Hz, 2H, $OC\underline{H}_2CH_3$), 3.22 (t, ^3J = 6.9 Hz, 2H, $C\underline{H}_2CH_2CH_2SH$), 2.65 (t, ^3J = 7.0 Hz, 2H, $CH_2CH_2C\underline{H}_2SH$), 1.91 (m, 2H, $CH_2C\underline{H}_2CH_2SH$), 1.37 (t, ^3J = 7.0 Hz, 3H, $OCH_2C\underline{H}_3$).

^{13}C NMR (100 MHz, $CDCl_3$) δ: 151.5 (Ph C-4), 142.2 (Ph C-1), 115.8 (Ph C-3,5), 114.2 (Ph C-2,6), 64.1 ($O\underline{C}H_2CH_3$), 43.4 ($\underline{C}H_2CH_2CH_2SH$), 33.5 ($CH_2\underline{C}H_2CH_2SH$), 22.3 ($CH_2CH_2\underline{C}H_2SH$), 15.0 ($OCH_2\underline{C}H_3$).

HRMS (ESI), *m/z*: calcd. for $C_{11}H_{16}NOS^+$: 212.1104 [M + H]$^+$; found: 212.1109.

3-[(4-ethoxyphenyl)amino](3,3-$^2H_2$)-1-propanethiol (37)

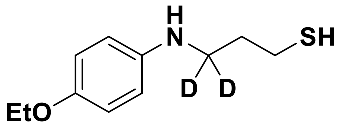

To a solution of 4-ethoxy-*N*-[(2*Z*)-2-thietanylidene]aniline (0.124 g, 0.6 mmol, 1.0 equiv) in dry THF (2 mL) solid $LiAlH_4$-d_4 (0.025 g, 0.6 mmol, 1.0 equiv) was added in one portion at 0 °C. The reaction mixture was allowed to reach room temperature and then stirred for 1.5 hours before being quenched with water. The reaction mixture was extracted exhaustively with Et_2O (3 x 10 mL). The combined organic extracts were washed with brine, dried over Na_2SO_4 and the solvent was removed under reduced pressure. The residue was purified by column chromatography on silica gel (*n*-hexane:EtOAc 9:1) to give compound **37** in 96% yield (0.123 g) as a yellow oil.

^{1}H NMR (400 MHz, $CDCl_3$) δ: 6.78 (m, 2H, Ph H-3,5), 6.58 (m, 2H, Ph H-2,6), 3.96 (q, ^3J = 7.0 Hz, 2H, $OC\underline{H}_2CH_3$), 2.65 (t, ^3J = 7.0 Hz, 2H, $CD_2CH_2C\underline{H}_2SH$), 1.90 (t, ^3J = 7.0 Hz, 2H, $CD_2C\underline{H}_2CH_2SH$), 1.37 (t, ^3J = 7.0 Hz, 3H, $OCH_2C\underline{H}_3$).

^{13}C NMR (100 MHz, $CDCl_3$) δ: 151.4 (Ph C-4), 142.3 (Ph C-1), 115.8 (Ph C-3,5), 114.2 (Ph C-2,6), 64.1 ($O\underline{C}H_2CH_3$), 42.6 (q, $^1J_{C,D}$ = 20.5 Hz, $\underline{C}D_2CH_2CH_2SH$), 33.3 ($CD_2\underline{C}H_2CH_2SH$), 22.2 ($CD_2CH_2\underline{C}H_2SH$), 15.0 ($OCH_2\underline{C}H_3$).

^{15}N NMR (40 MHz, $CDCl_3$) δ: -320.9.

HRMS (ESI), *m/z*: calcd. for $C_{11}H_{14}D_2NOS^+$: 214.1229 $[M + H]^+$; found: 214.1231.

3-[(4-methylphenyl)amino]-1-propanethiol (38)

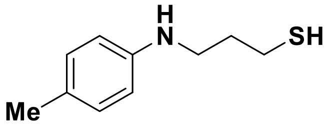

To a solution of 4-methyl-*N*-[(2*Z*)-2-thietanylidene]aniline (0.124 g, 0.7 mmol, 1.0 equiv) in dry THF (2 mL) a solution of $LiAlH_4$ (1M in THF, 0.7 mL, 0.7 mmol, 1.0 equiv) was added dropwise at 0 °C. The reaction mixture was allowed to reach room temperature and then stirred for 1.5 hours before being quenched with water. The reaction mixture was extracted exhaustively with Et_2O (3 x 10 mL). The combined organic extracts were washed with brine, dried over Na_2SO_4 and the solvent was removed under reduced pressure. The residue was purified by column chromatography on silica gel (*n*-hexane:EtOAc 8:2) to give compound **38** in 85% yield (0.108 g) as a yellow oil.

^{1}H NMR (400 MHz, $CDCl_3$) δ: 7.02 (m, 2H, Ph H-3,5), 6.67 (m, 2H, Ph H-2,6), 3.26 (t, ^3J = 6.8 Hz, 2H, $\underline{C}H_2CH_2CH_2SH$), 2.64 (t, ^3J = 6.8 Hz, 2H, $CH_2CH_2\underline{C}H_2SH$), 2.26 (s, 3H, CH_3), 1.95 (q, ^3J = 6.9 Hz, 2H, $CH_2C\underline{H}_2CH_2SH$).

^{13}C NMR (100 MHz, $CDCl_3$) δ: 144.1 (Ph C-1), 129.9 (Ph C-3,5), 128.3 (Ph C-4), 114.2 (Ph C-2,6), 43.7 ($\underline{C}H_2CH_2CH_2SH$), 32.9 ($CH_2\underline{C}H_2CH_2SH$), 22.1 ($CH_2CH_2\underline{C}H_2SH$), 20.4 ($CH_3$).

^{15}N NMR (40 MHz, $CDCl_3$) δ: -318.2.

HRMS (ESI), *m/z*: calcd. for $C_{10}H_{16}NS^+$: 182.0998 $[M + H]^+$; found: 182.0999.

3-[(3,5-dichlorophenyl)amino]-1-propanethiol (39)

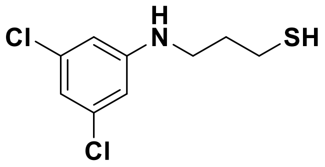

To a solution of 3,5-dichloro-*N*-[(2*Z*)-2-thietanylidene]aniline (0.162 g, 0.7 mmol, 1.0 equiv) in dry THF (2 mL) a solution of $LiAlH_4$ (1M in THF, 0.7 mL, 0.7 mmol, 1.0 equiv) was added dropwise at 0 °C. The reaction mixture was allowed to reach room temperature and then stirred for 1.5 hours before being quenched with water. The reaction mixture was extracted exhaustively with Et_2O (3 x 10 mL). The combined organic extracts were washed with brine, dried over Na_2SO_4 and the solvent was removed under reduced pressure. The residue was purified by column chromatography on silica gel (*n*-hexane:EtOAc 8:2) to give compound **39** in 92% yield (0.152 g) as a yellow oil.

^{1}H NMR (400 MHz, $CDCl_3$) δ: 6.66 (t, ^4J = 1.8 Hz, 1H, Ph H-4), 6.46 (d, ^4J = 1.8 Hz, 2H, Ph H-2,6), 3.89 (brs, 1H, NH), 3.24 (t, ^3J = 6.8 Hz, 2H, $\underline{C}H_2CH_2CH_2SH$), 2.64 (m, 2H, $CH_2CH_2C\underline{H}_2SH$), 1.91 (q, ^3J = 6.8 Hz, 2H, $CH_2\underline{C}H_2CH_2SH$), 1.41 (t, ^3J = 7.9 Hz, 1H, SH).

^{13}C NMR (100 MHz, $CDCl_3$) δ: 149.6 (Ph C-1), 135.5 (Ph C-3,5), 117.1 (Ph C-4), 110.9 (Ph C-2,6), 42.0 ($\underline{C}H_2CH_2CH_2SH$), 32.9 ($CH_2\underline{C}H_2CH_2SH$), 22.0 ($CH_2CH_2\underline{C}H_2SH$).

HRMS (ESI), *m/z*: calcd. for $C_9H_{12}Cl_2NS^+$: 236.0062 $[M + H]^+$; found: 236.0064.

Evidence for the chemical integrity of unsubstituted imino-thietanes

As reported in the text, surprisingly unsubstituted imino-thietanes manifested a solid stability and, despite the variation of reaction conditions, ring-openings with distinct entities (than $LiAlH_4$ or $LiAlD_4$) were unsuccessful. Analogously, attempting the deprotonation at the *vic*-position followed by electrophilic trapping did not result in any transformation.

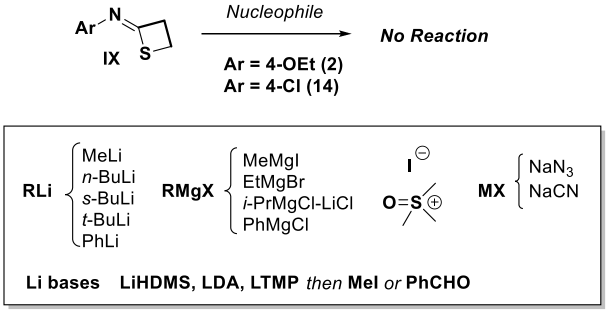

Attempts to induce reactivity via variation of temperature, solvent reaction time, stoichiometry

Additional element diagnostic for the extremely high integrity imparted by the net CH_2-CH_2 fragment was deducted upon thermal treatment *en route* to convert the skeletons into acrylic thioamides.

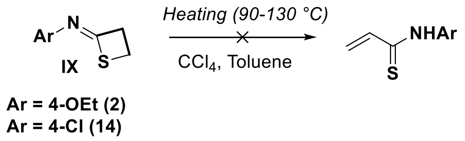

Unfortunately, no reactivity was observed: 1) under Lewis acid catalytic conditions (BF_3 OEt_2) for promoting the ring opening (with TMSCN); 2) under hydrolytic acid conditions and, 3) in the presence of the oxidant *m*-CPBA.

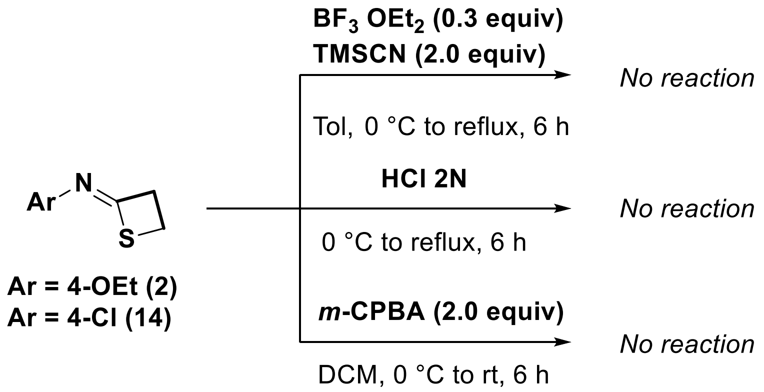

Copies of ^{1}H- and ^{13}C-NMR Spectra

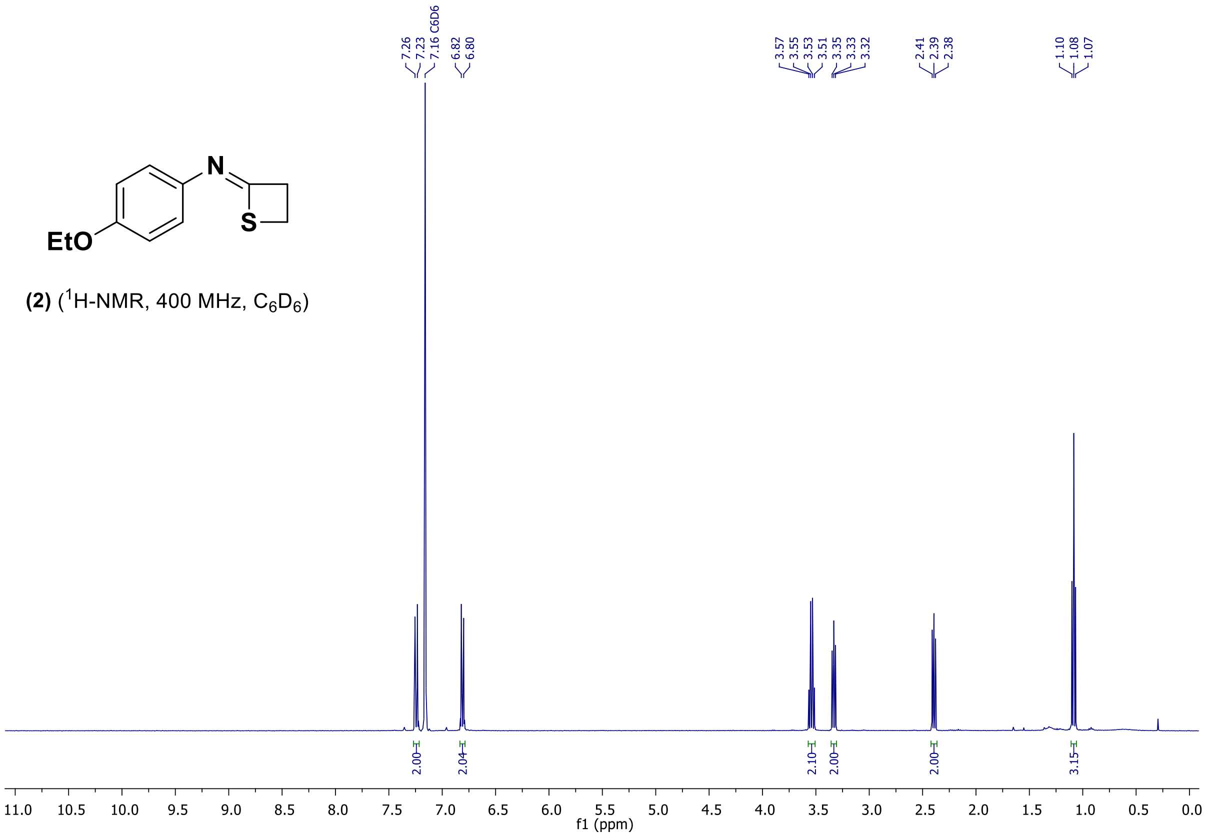

(2) (^{1}H-NMR, 400 MHz, C_6D_6)

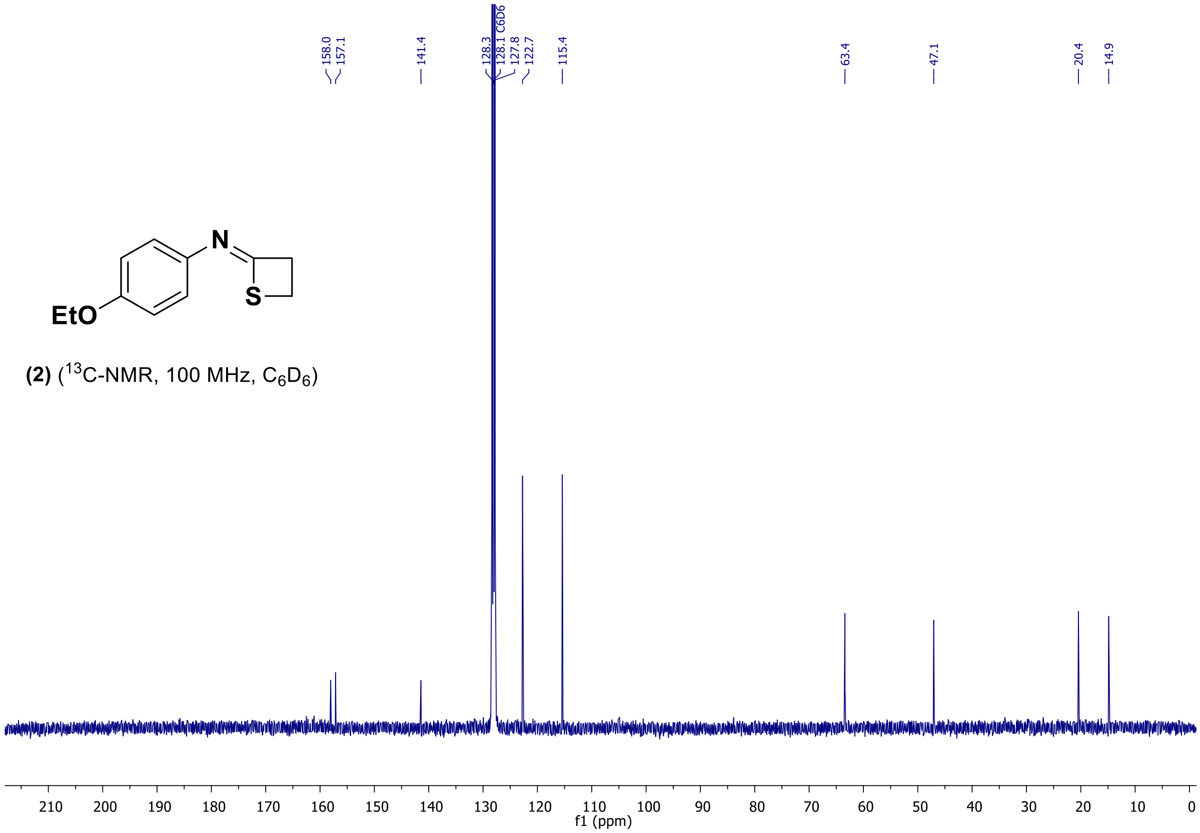

(2) (^{13}C-NMR, 100 MHz, C_6D_6)

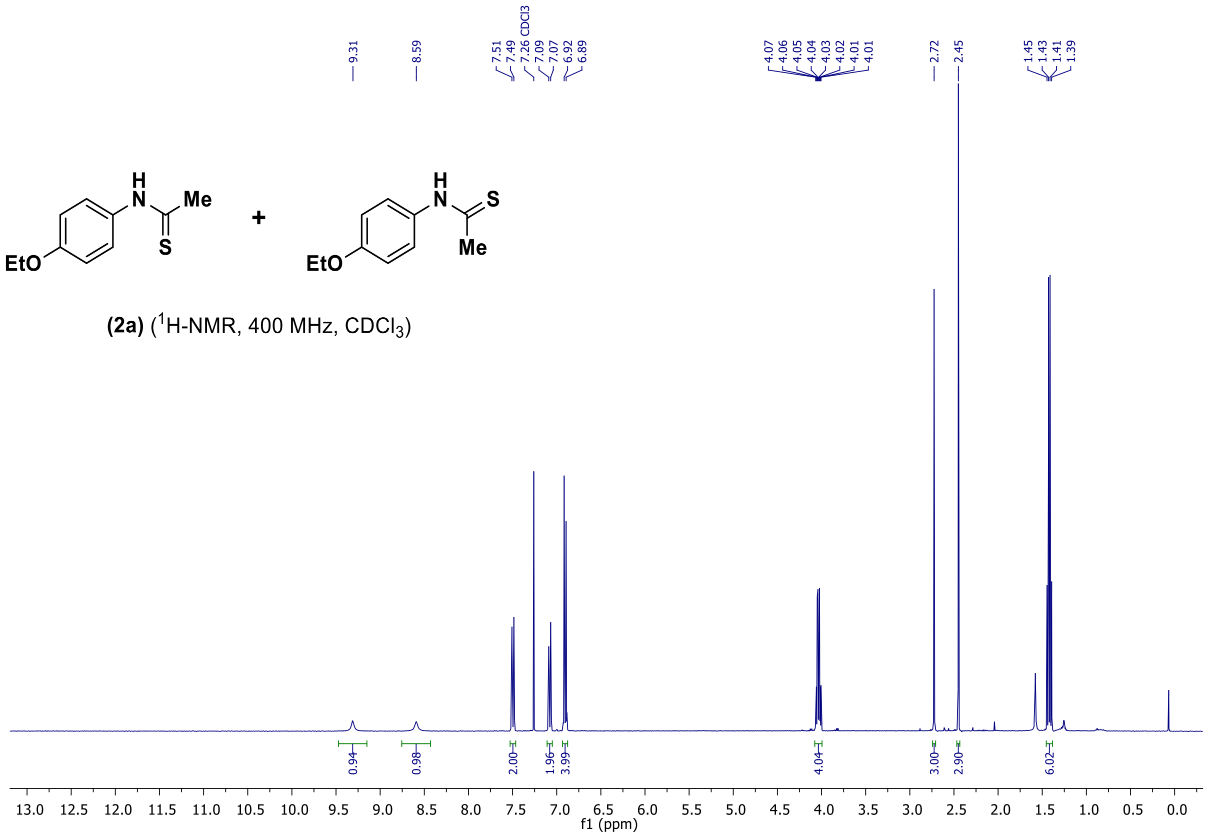

(2a) (^{1}H-NMR, 400 MHz, $CDCl_3$)

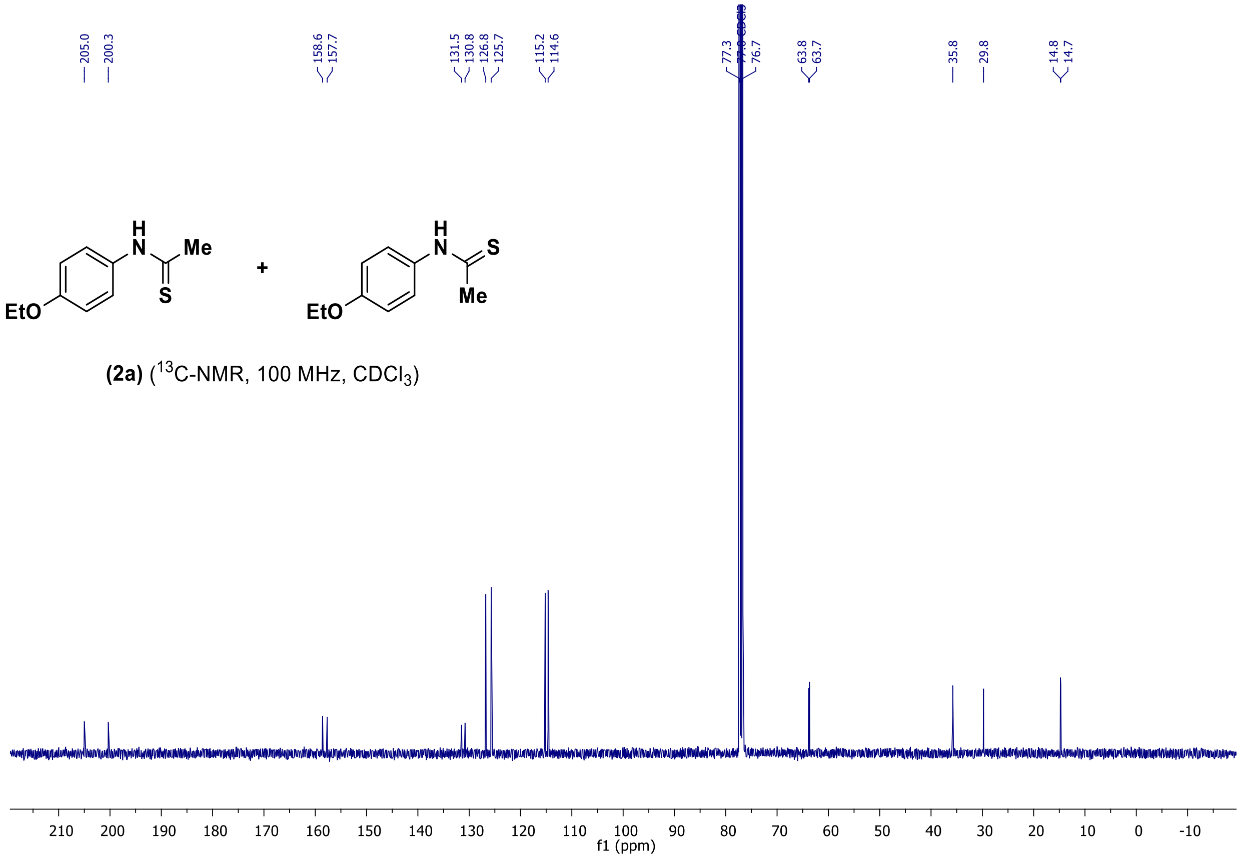

(2a) (^{13}C-NMR, 100 MHz, $CDCl_3$)

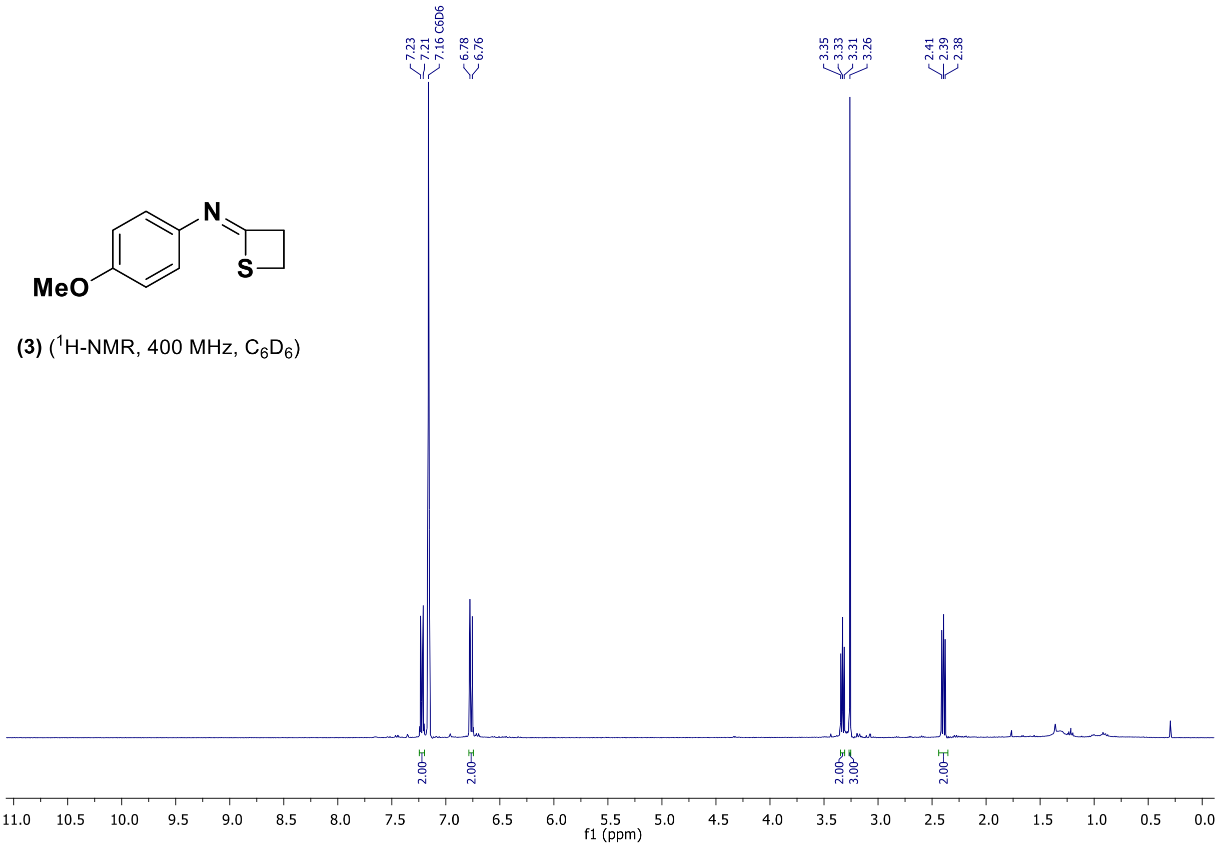

(3) (^{1}H-NMR, 400 MHz, C_6D_6)

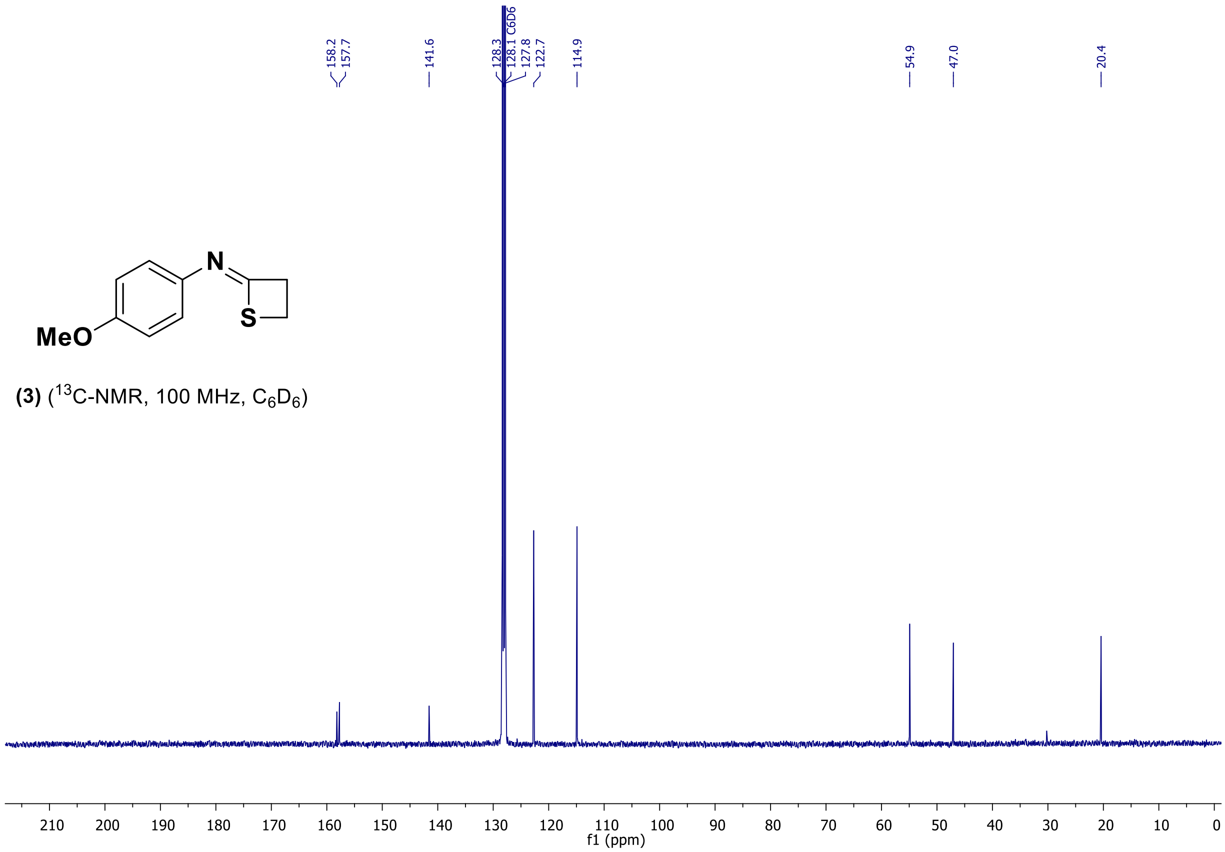

(3) (^{13}C-NMR, 100 MHz, C_6D_6)

(4) (^{1}H-NMR, 400 MHz, C_6D_6)

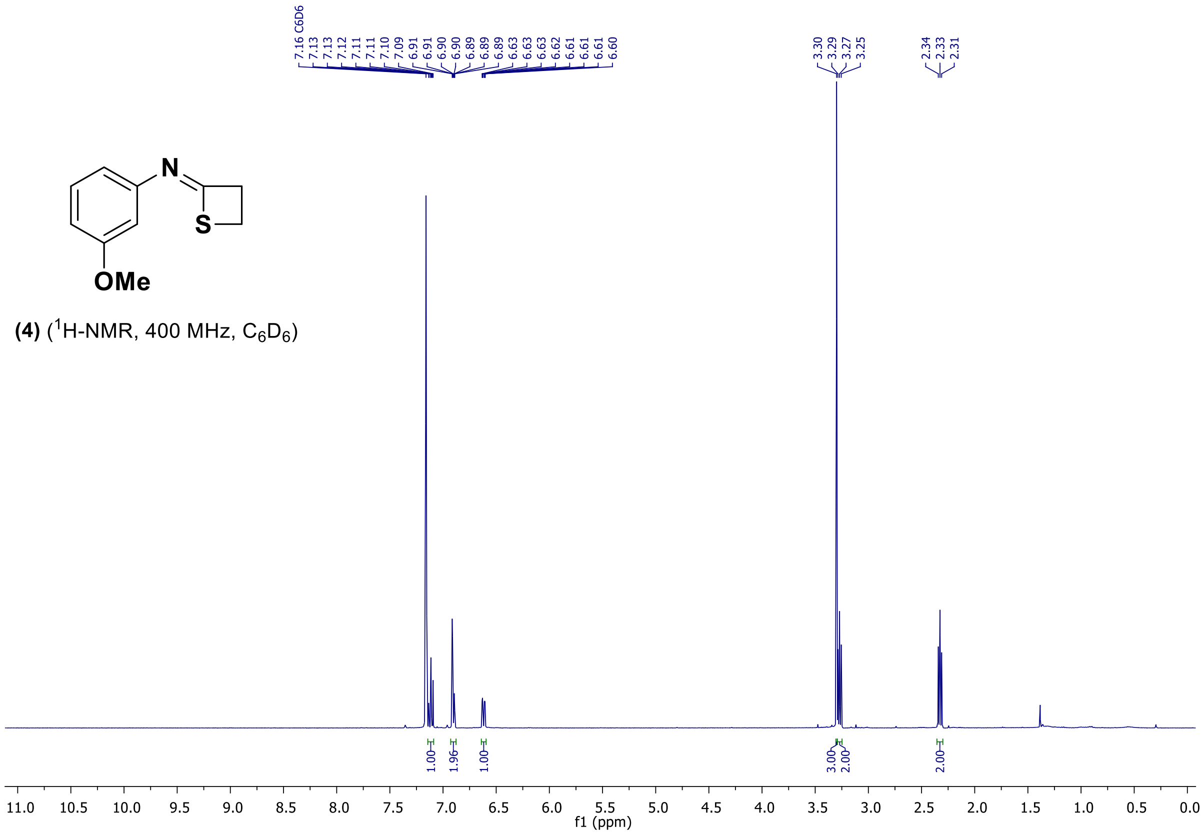

(4) (^{13}C-NMR, 100 MHz, C_6D_6)

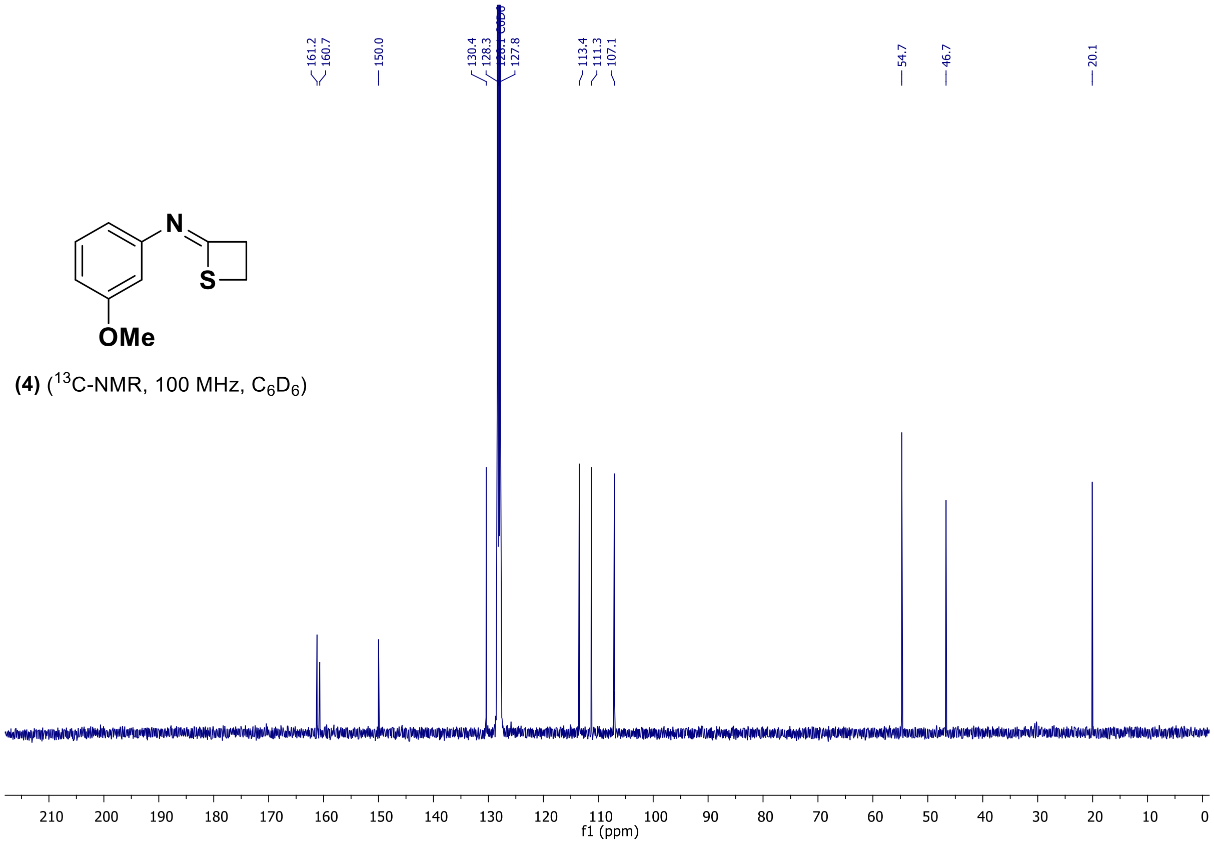

(5) (^{1}H-NMR, 400 MHz, C_6D_6)

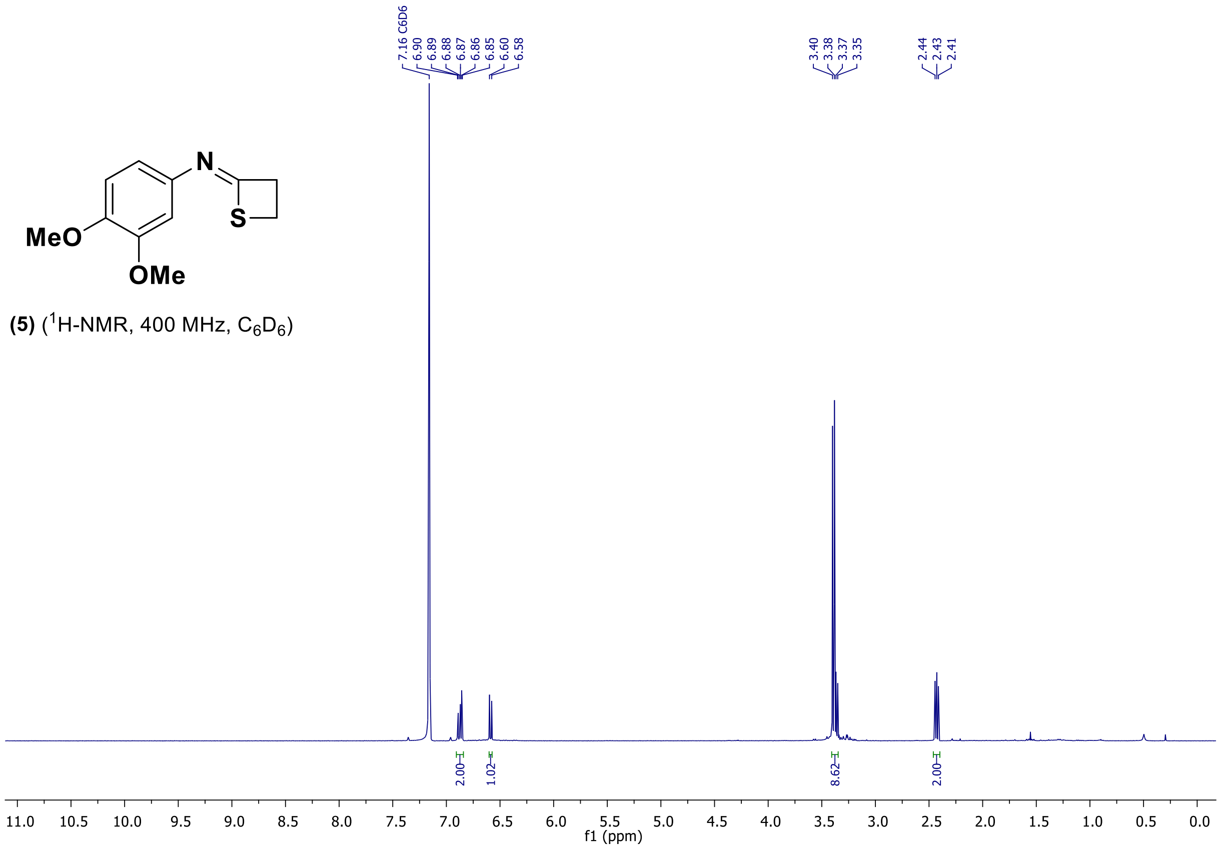

(5) (^{13}C-NMR, 100 MHz, C_6D_6)

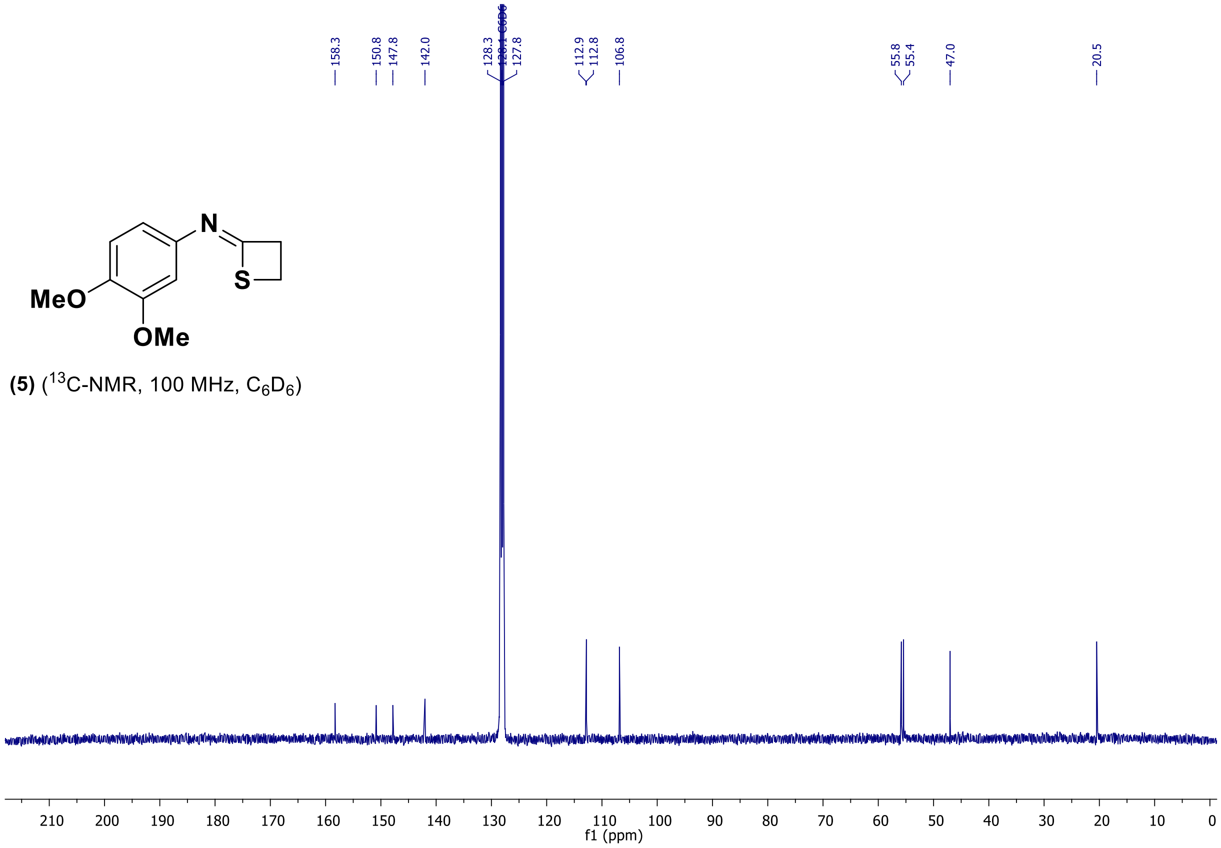

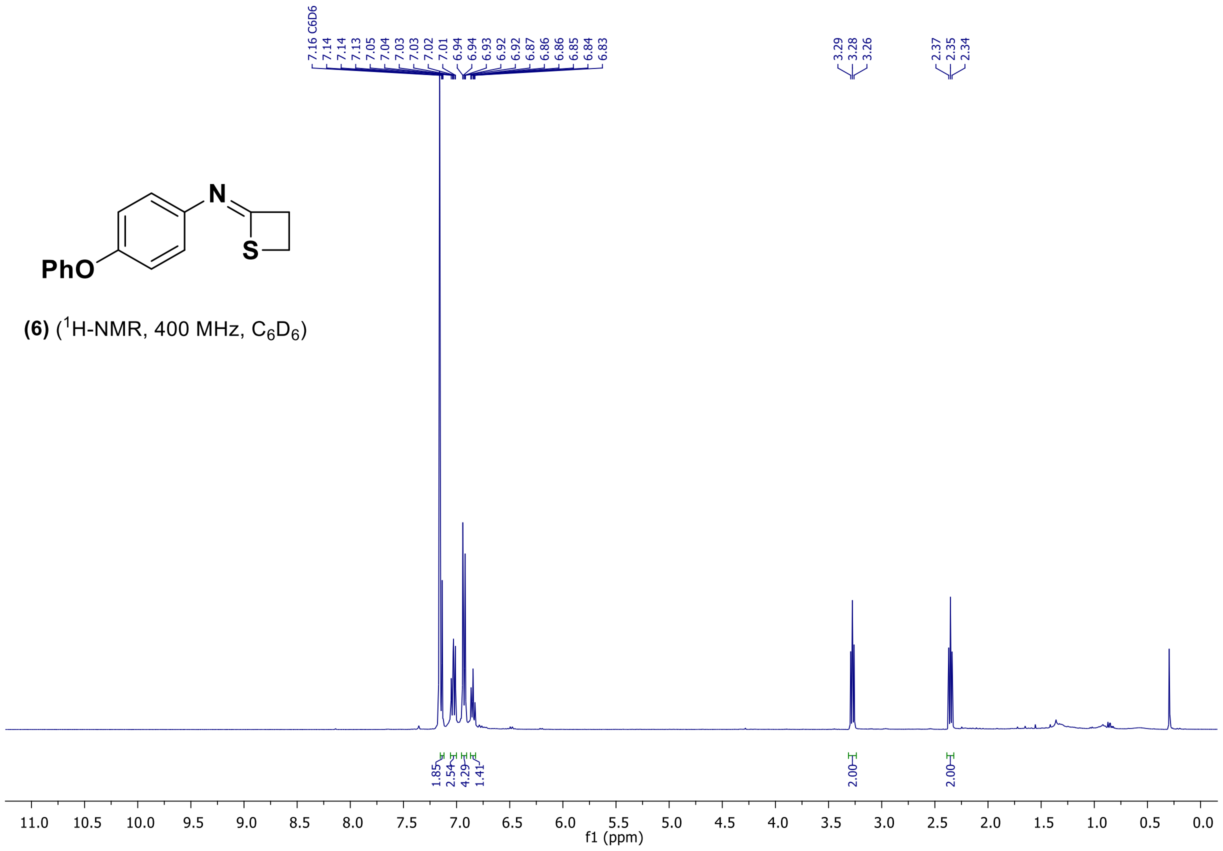

(6) (^{1}H-NMR, 400 MHz, C_6D_6)

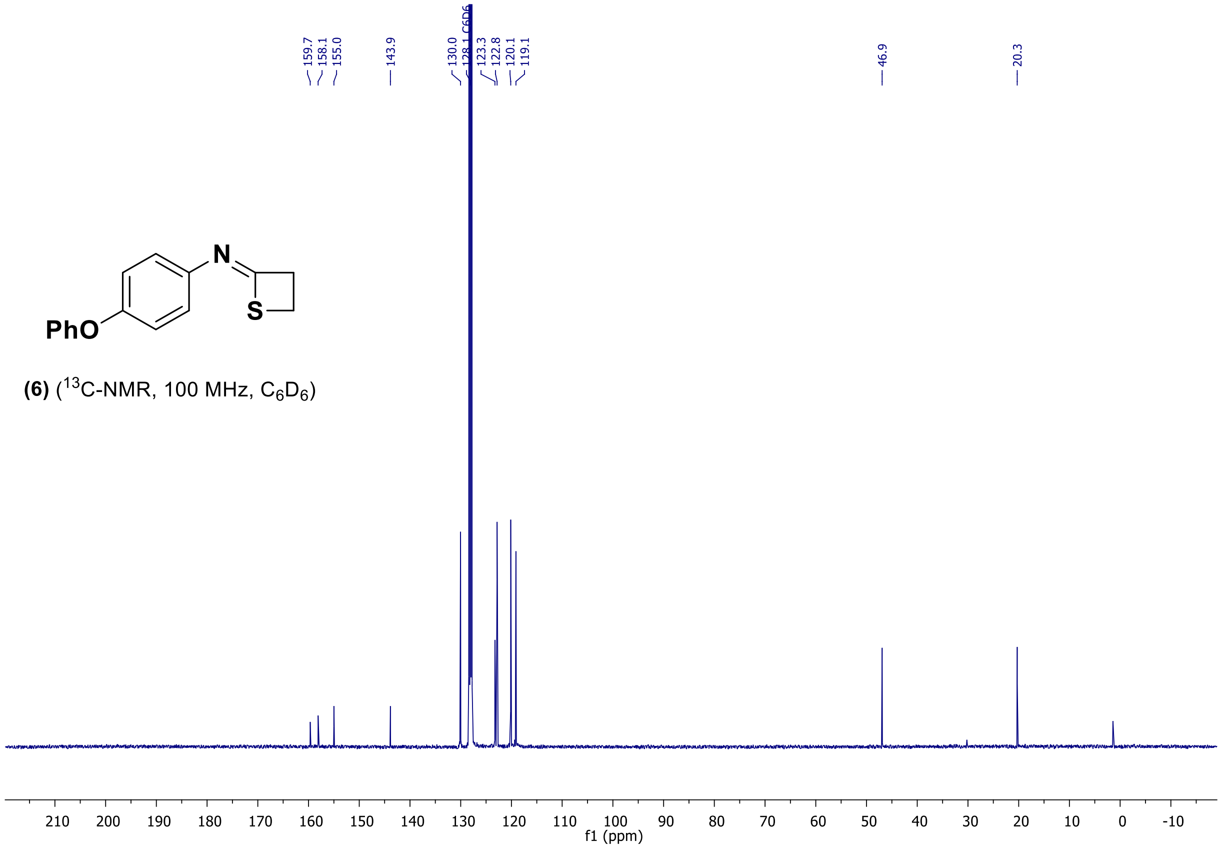

(6) (^{13}C-NMR, 100 MHz, C_6D_6)

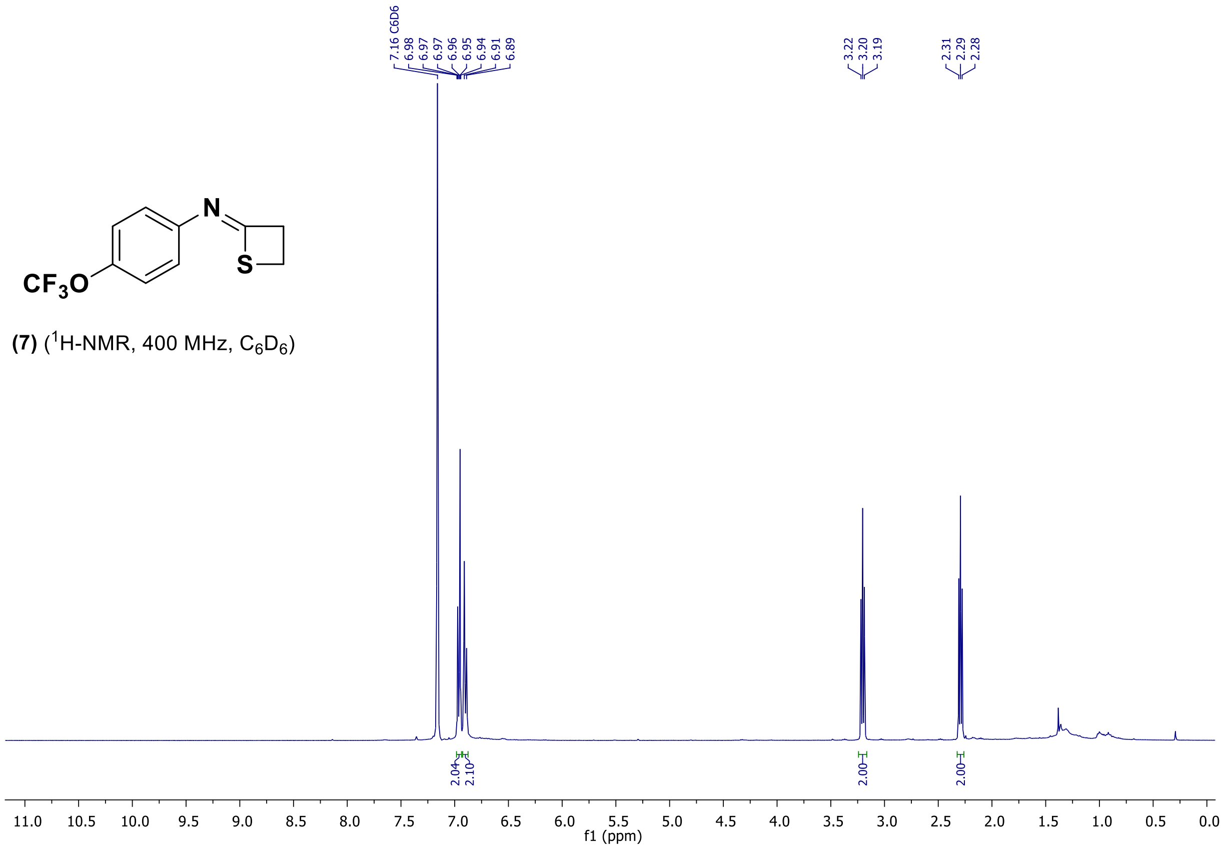

(7) (^{1}H-NMR, 400 MHz, C_6D_6)

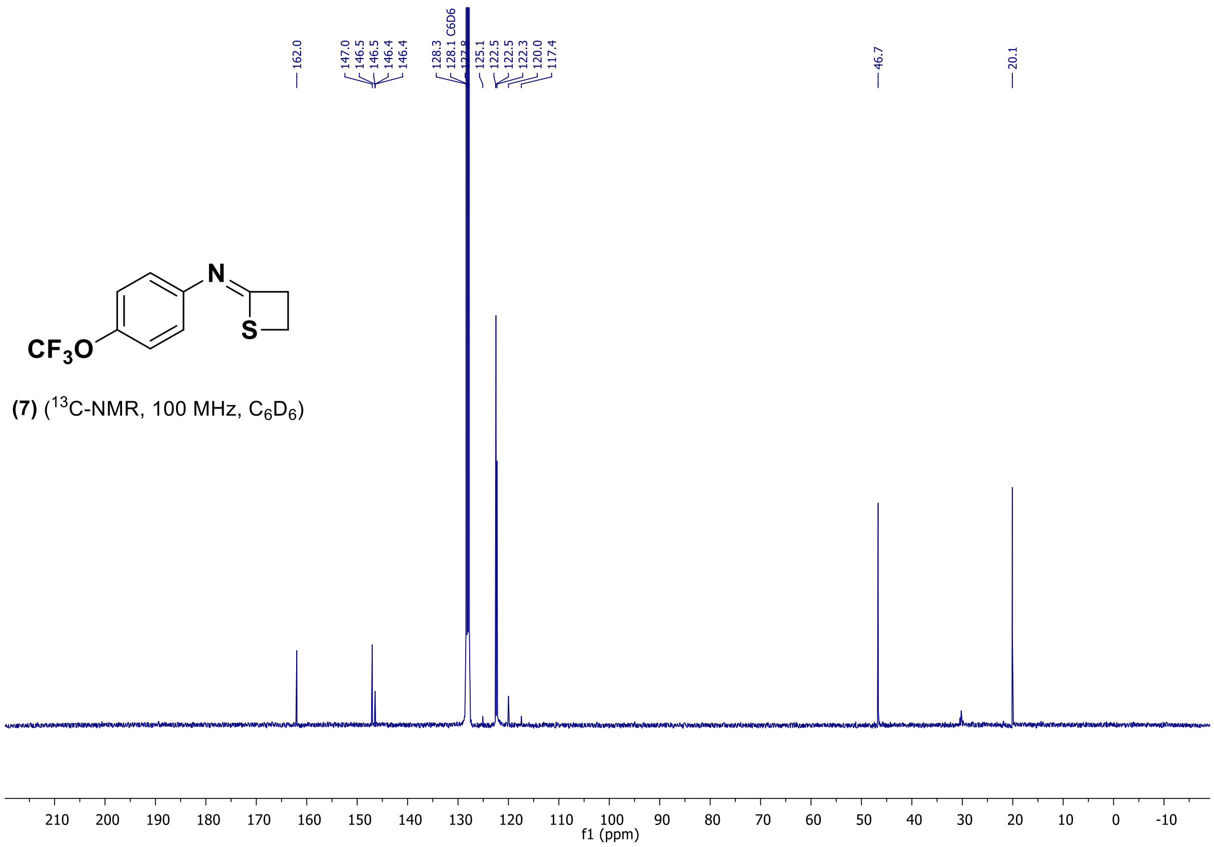

(7) (^{13}C-NMR, 100 MHz, C_6D_6)

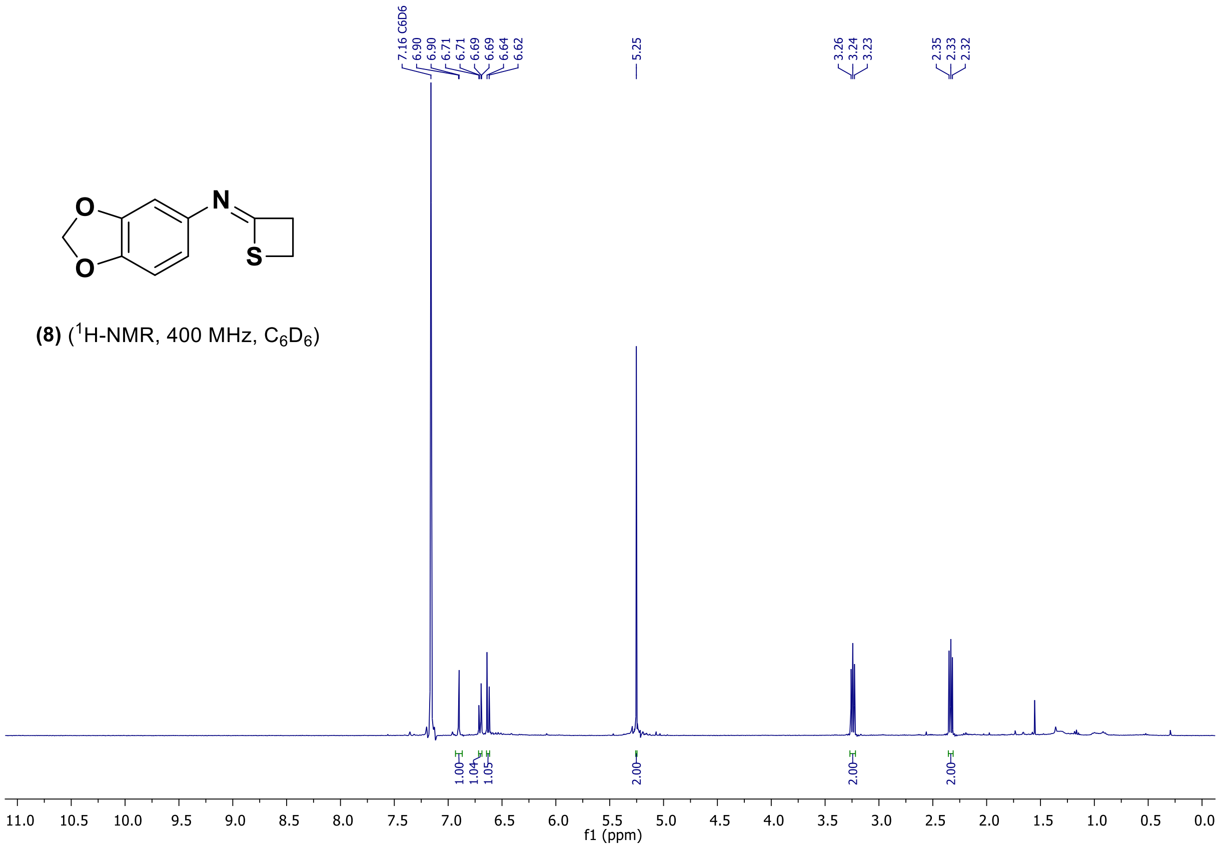

(8) (^{1}H-NMR, 400 MHz, C_6D_6)

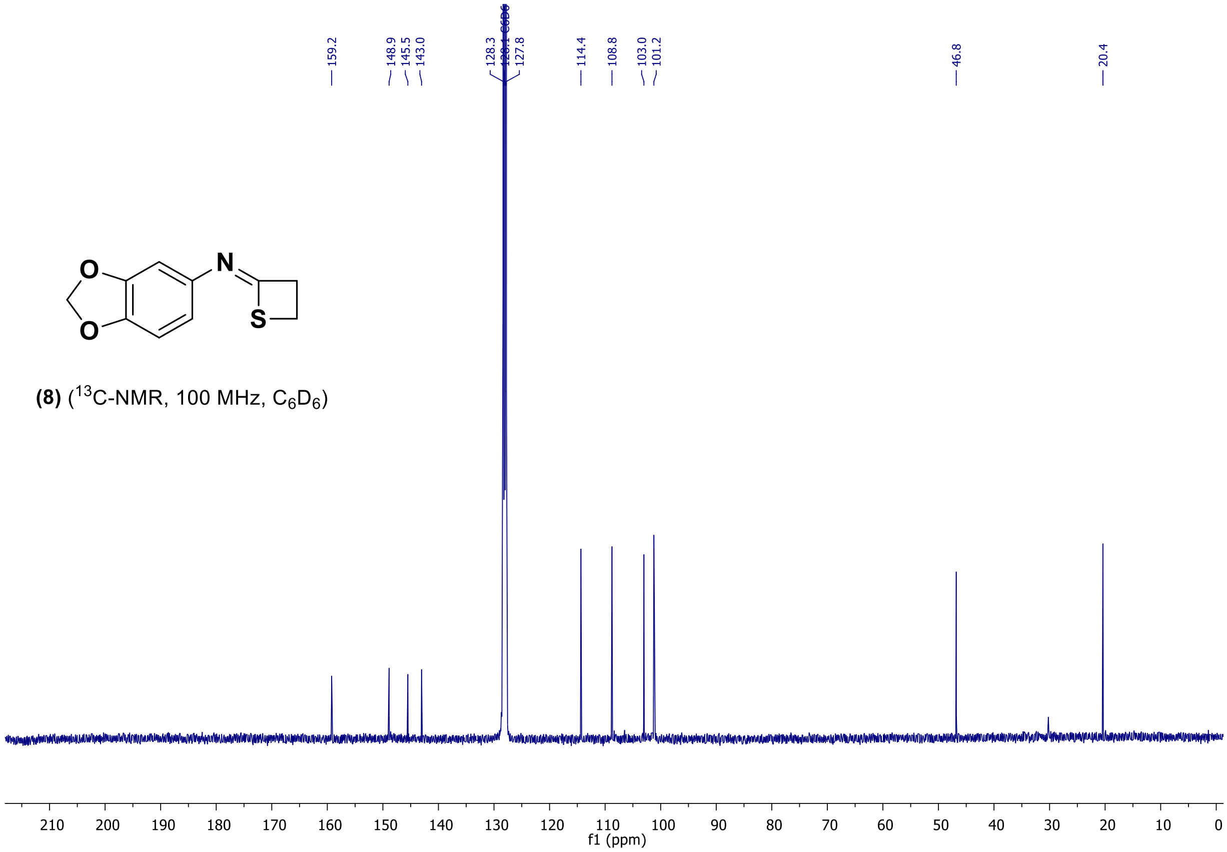

(8) (^{13}C-NMR, 100 MHz, C_6D_6)

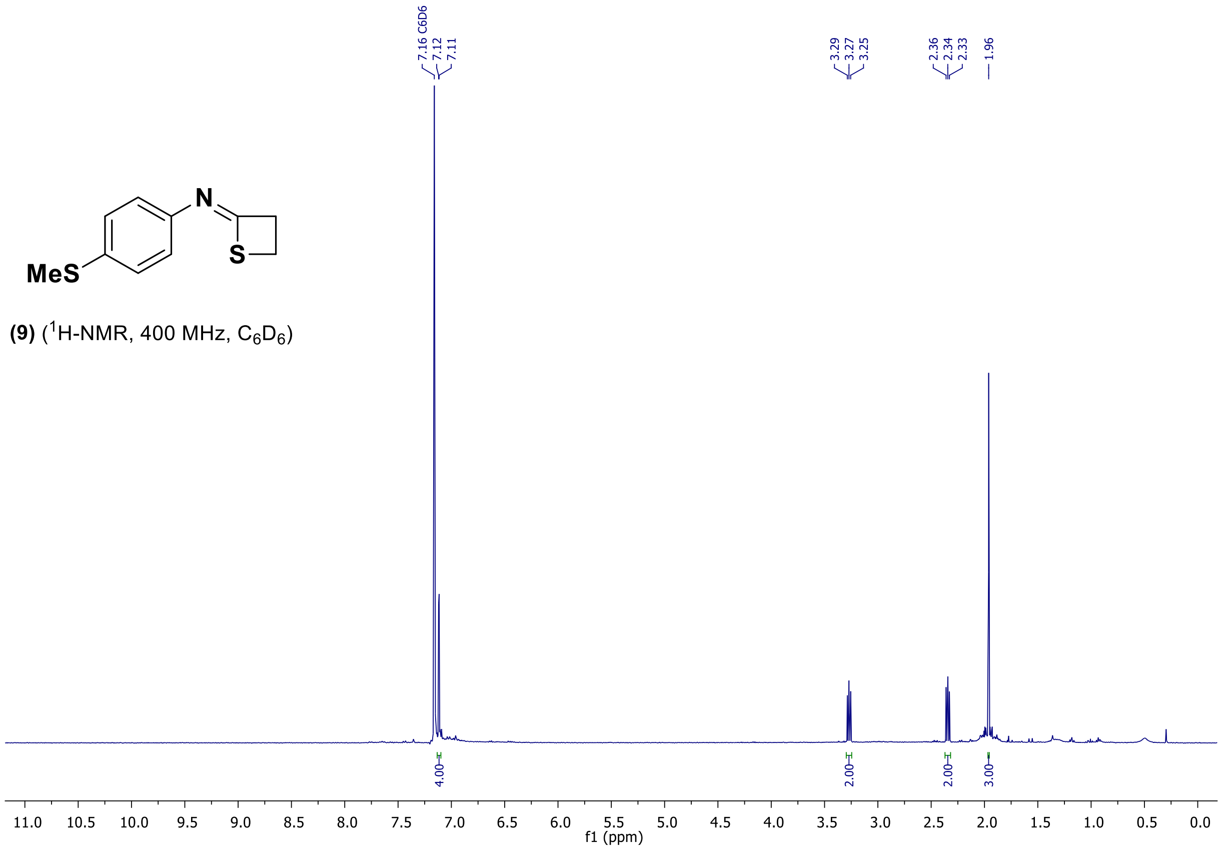

(9) (^{1}H-NMR, 400 MHz, C_6D_6)

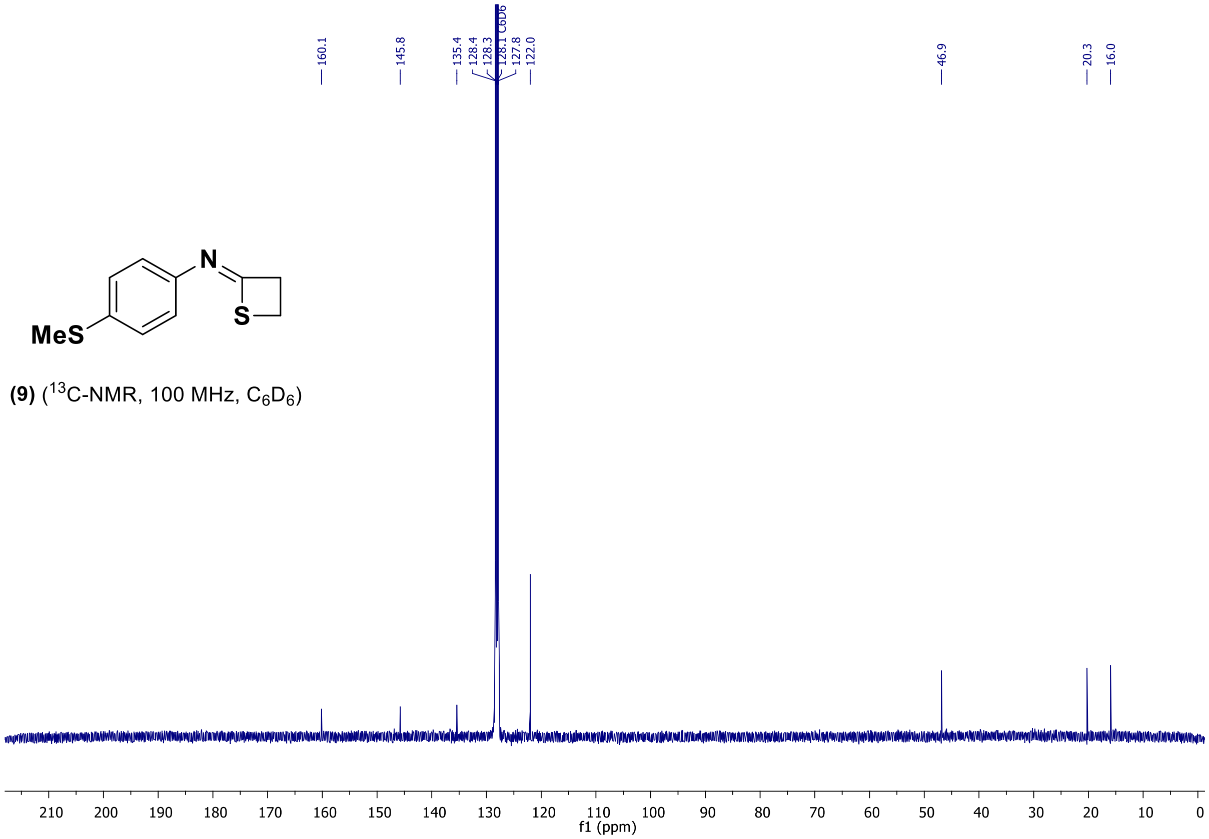

(9) (^{13}C-NMR, 100 MHz, C_6D_6)

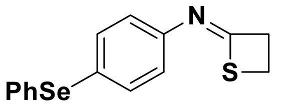

(10) (^{1}H-NMR, 400 MHz, C_6D_6)

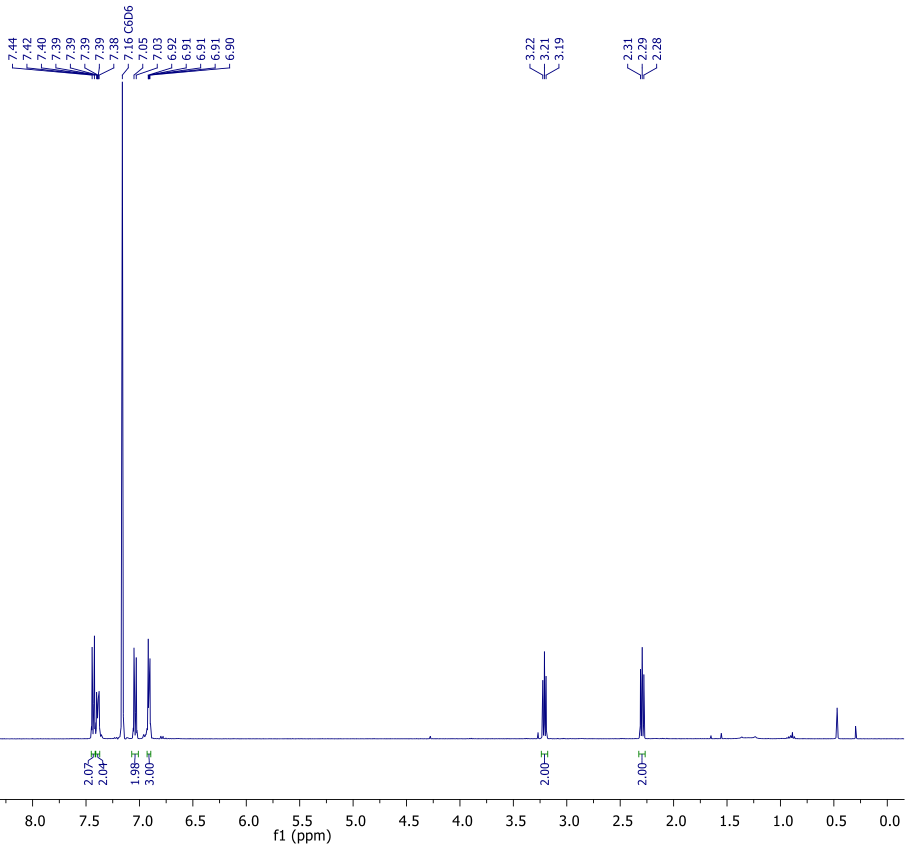

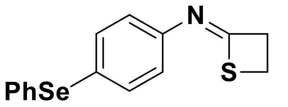

(10) (^{13}C-NMR, 100 MHz, C_6D_6)

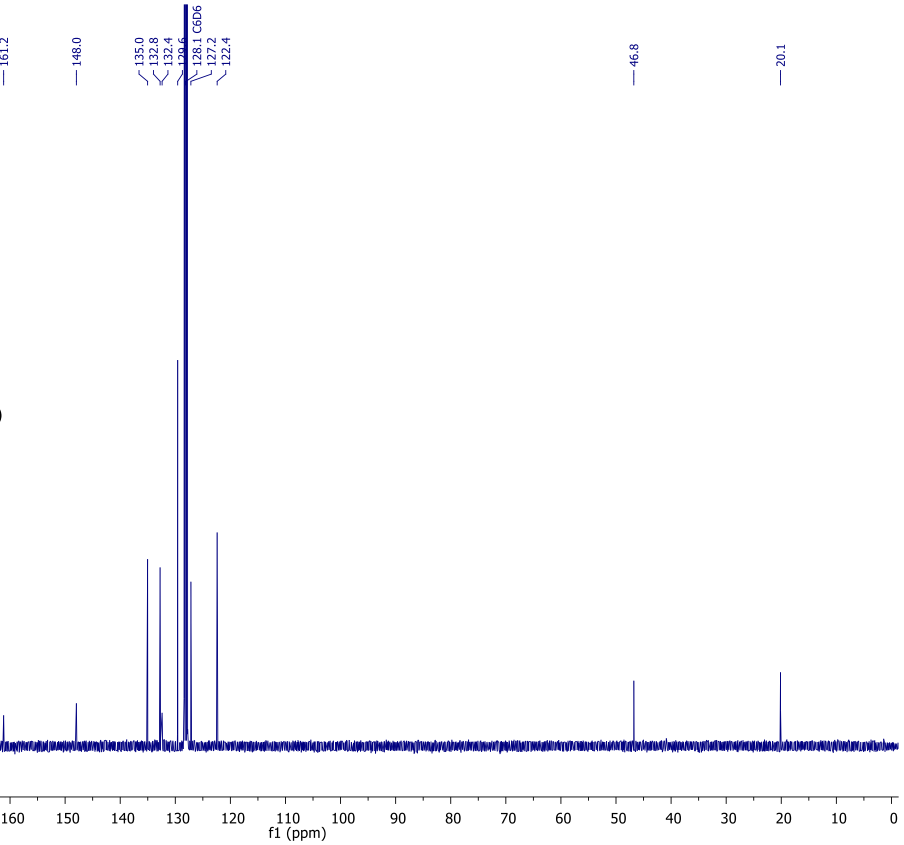

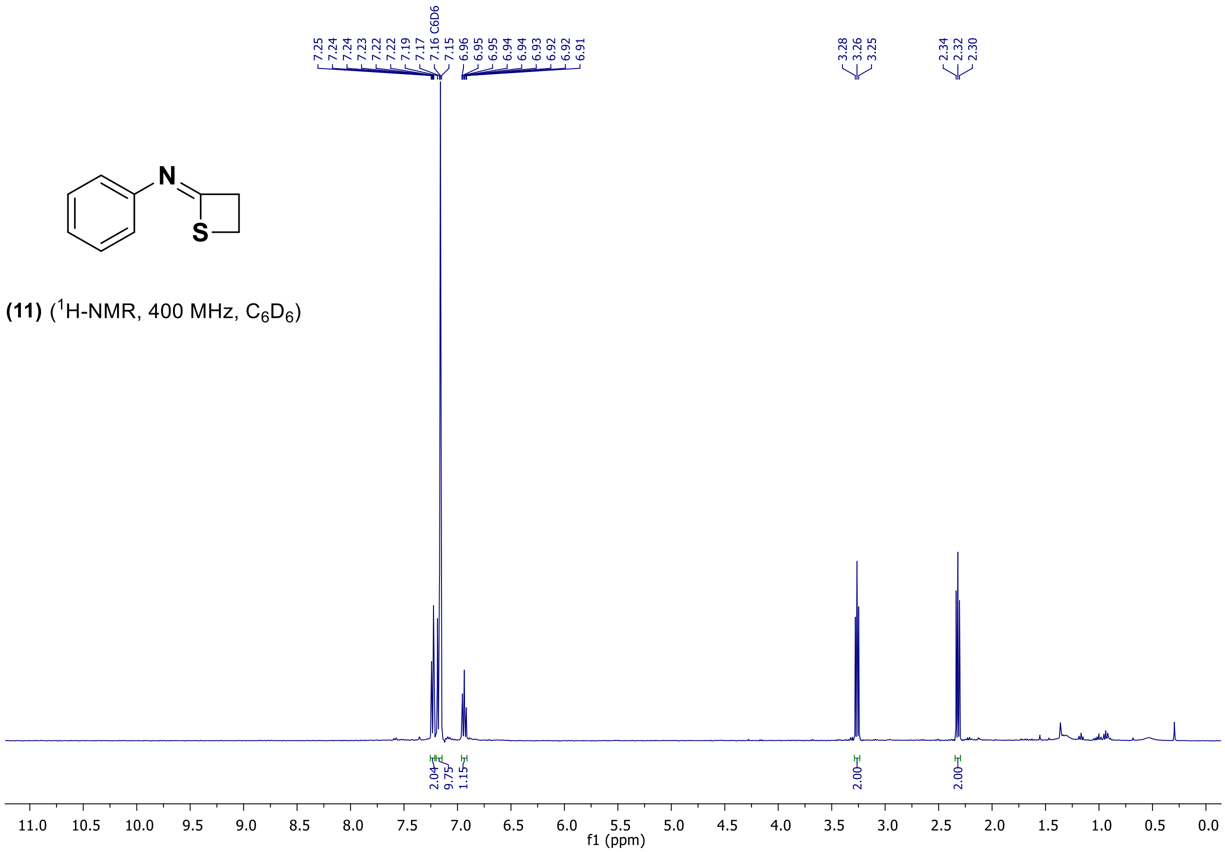

(11) (^{1}H-NMR, 400 MHz, C_6D_6)

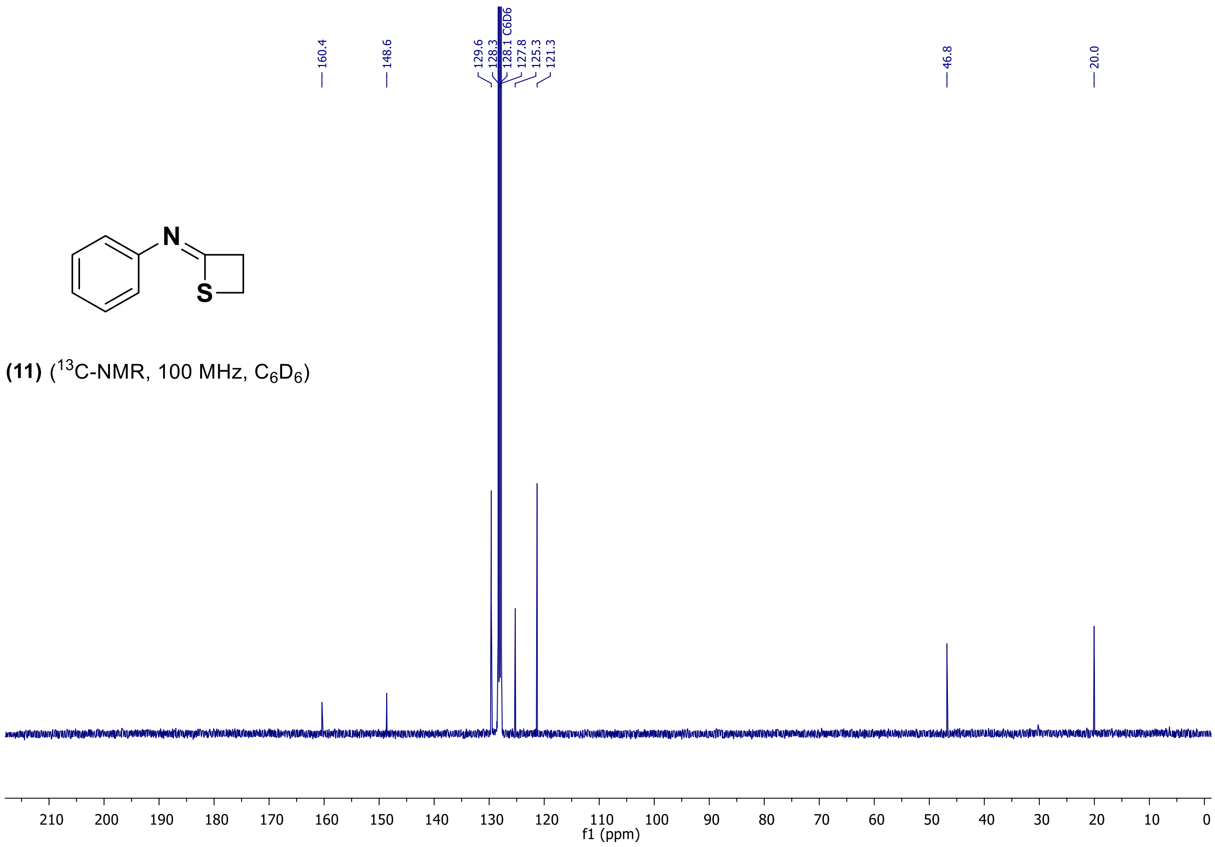

(11) (^{13}C-NMR, 100 MHz, C_6D_6)

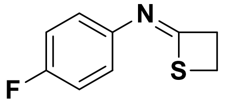

(12) (^{1}H-NMR, 400 MHz, C_6D_6)

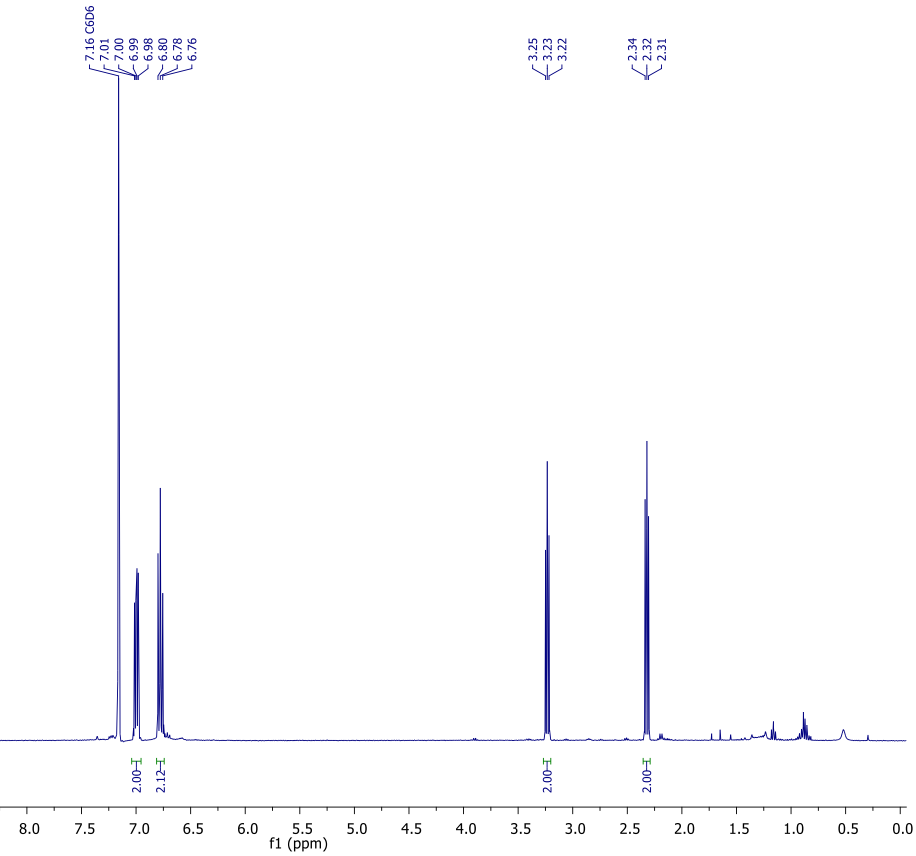

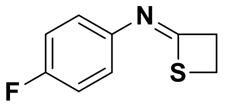

(12) (^{13}C-NMR, 100 MHz, C_6D_6)

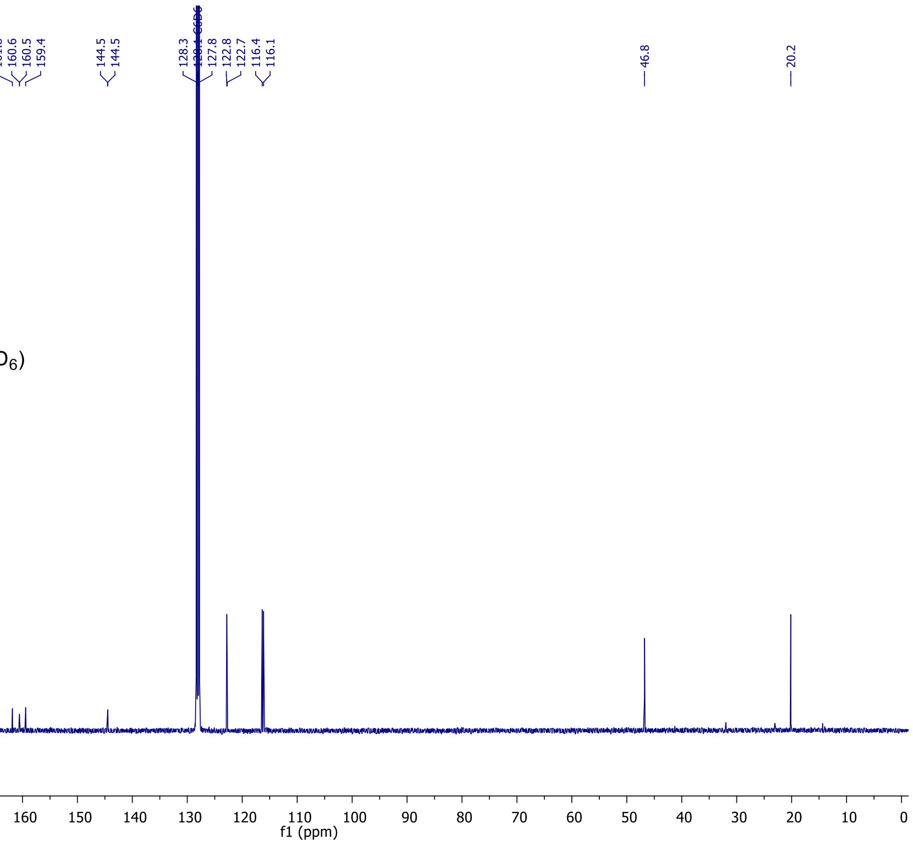

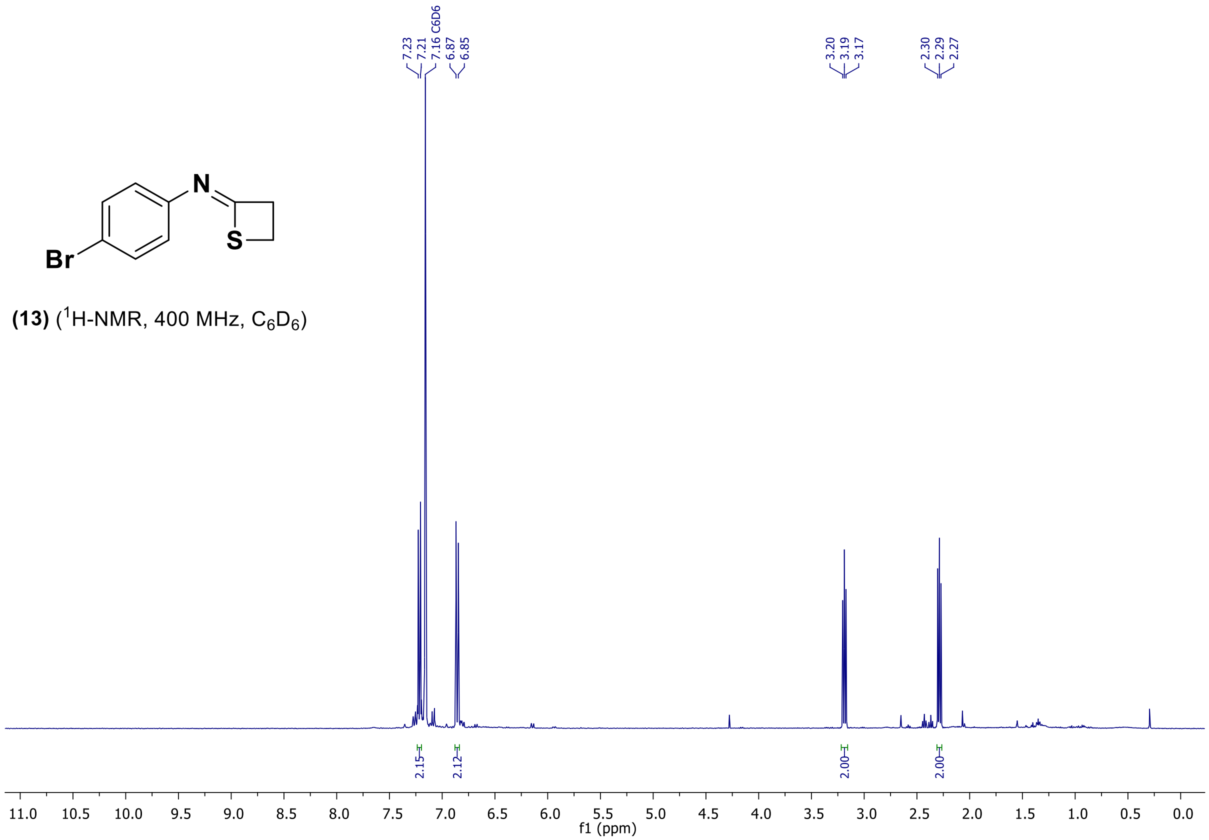

(13) (^{1}H-NMR, 400 MHz, C_6D_6)

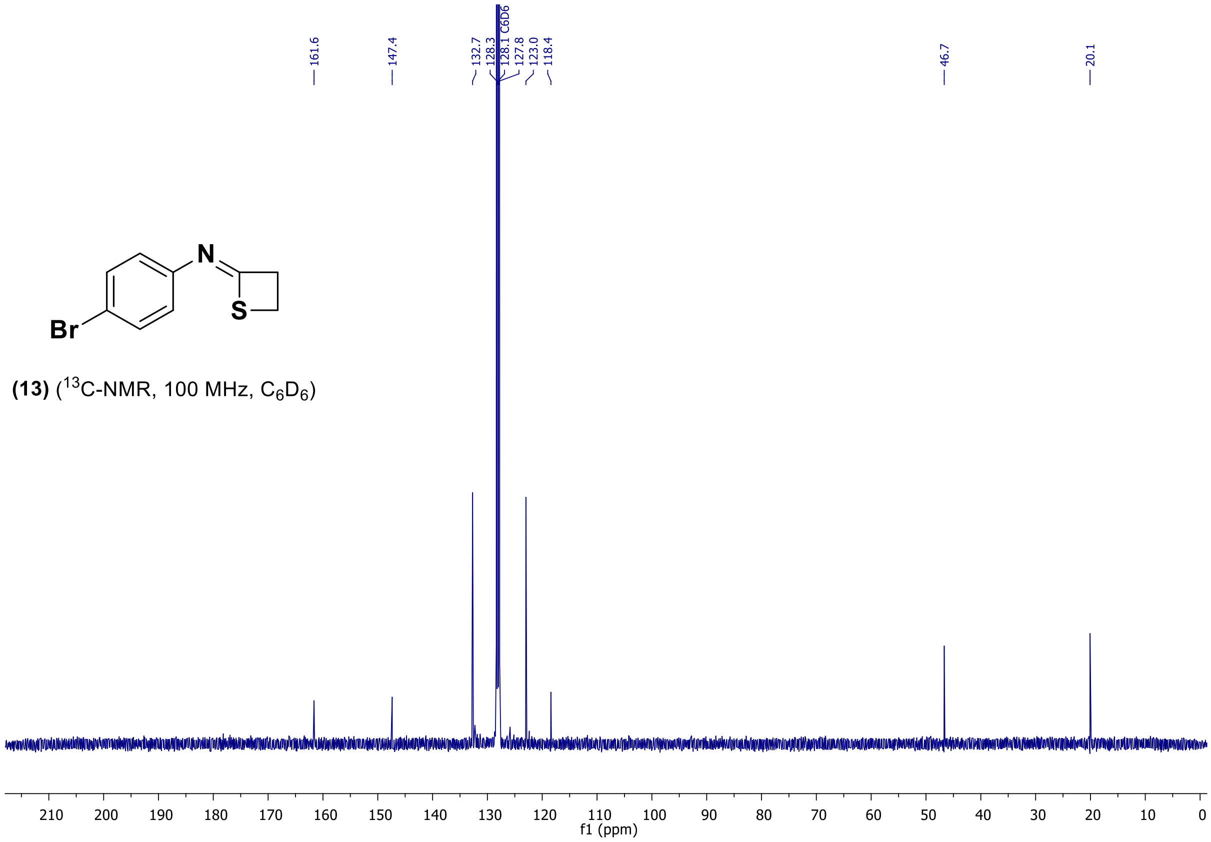

(13) (^{13}C-NMR, 100 MHz, C_6D_6)

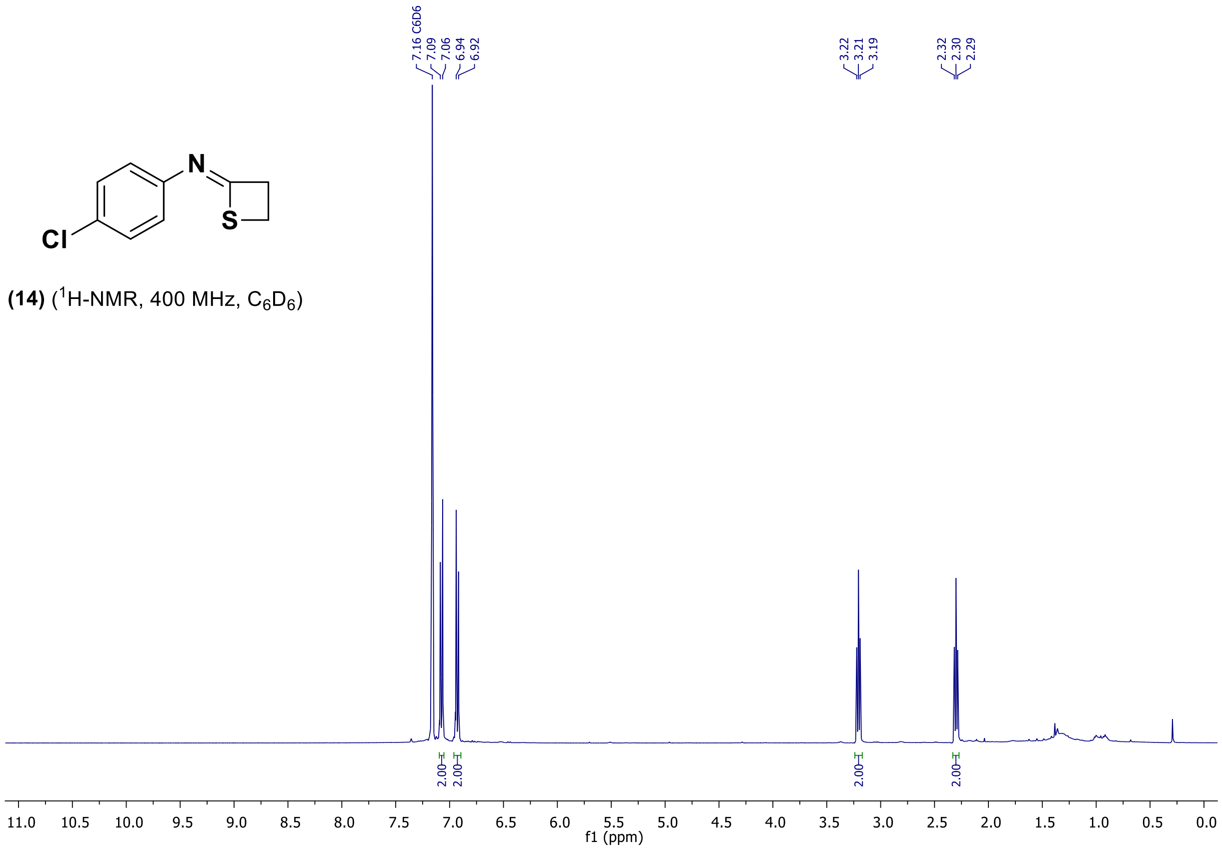

(14) (^{1}H-NMR, 400 MHz, C_6D_6)

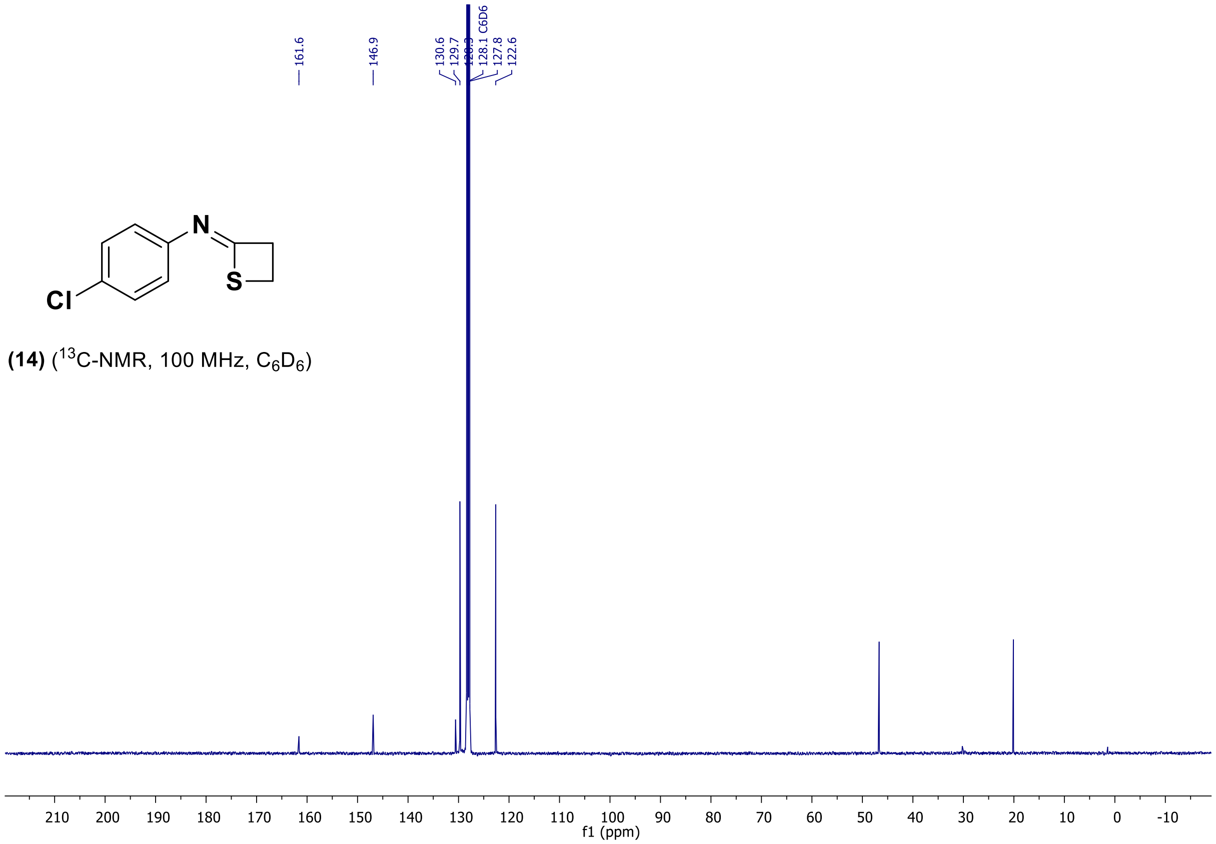

(14) (^{13}C-NMR, 100 MHz, C_6D_6)

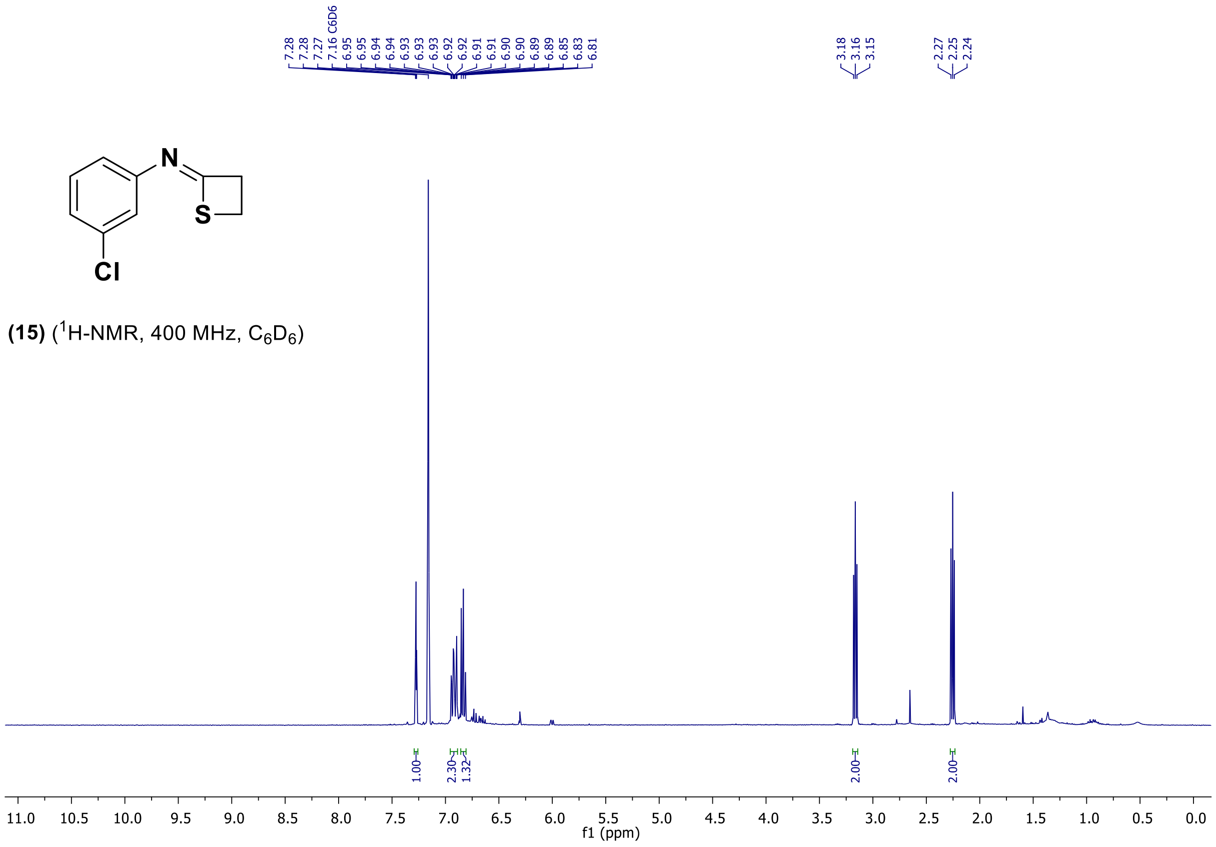

(15) (^{1}H-NMR, 400 MHz, C_6D_6)

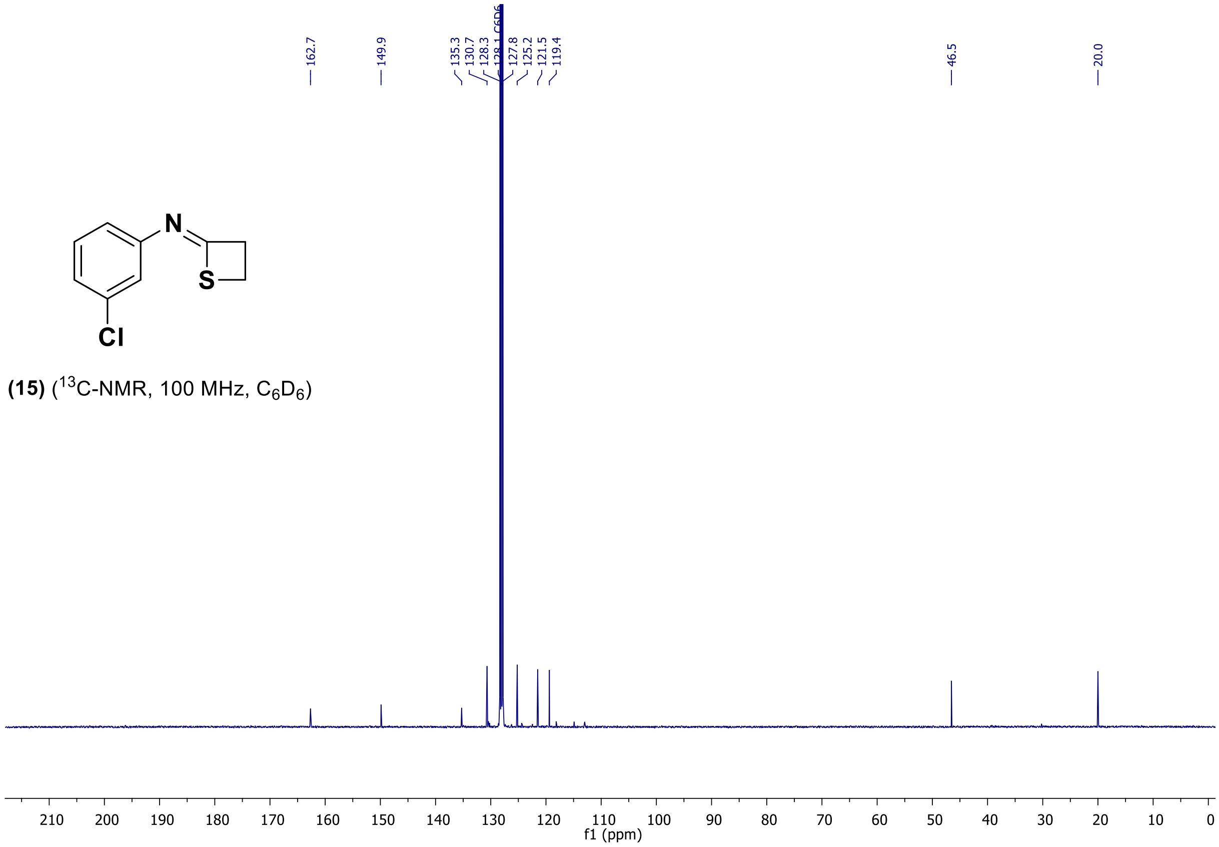

(15) (^{13}C-NMR, 100 MHz, C_6D_6)

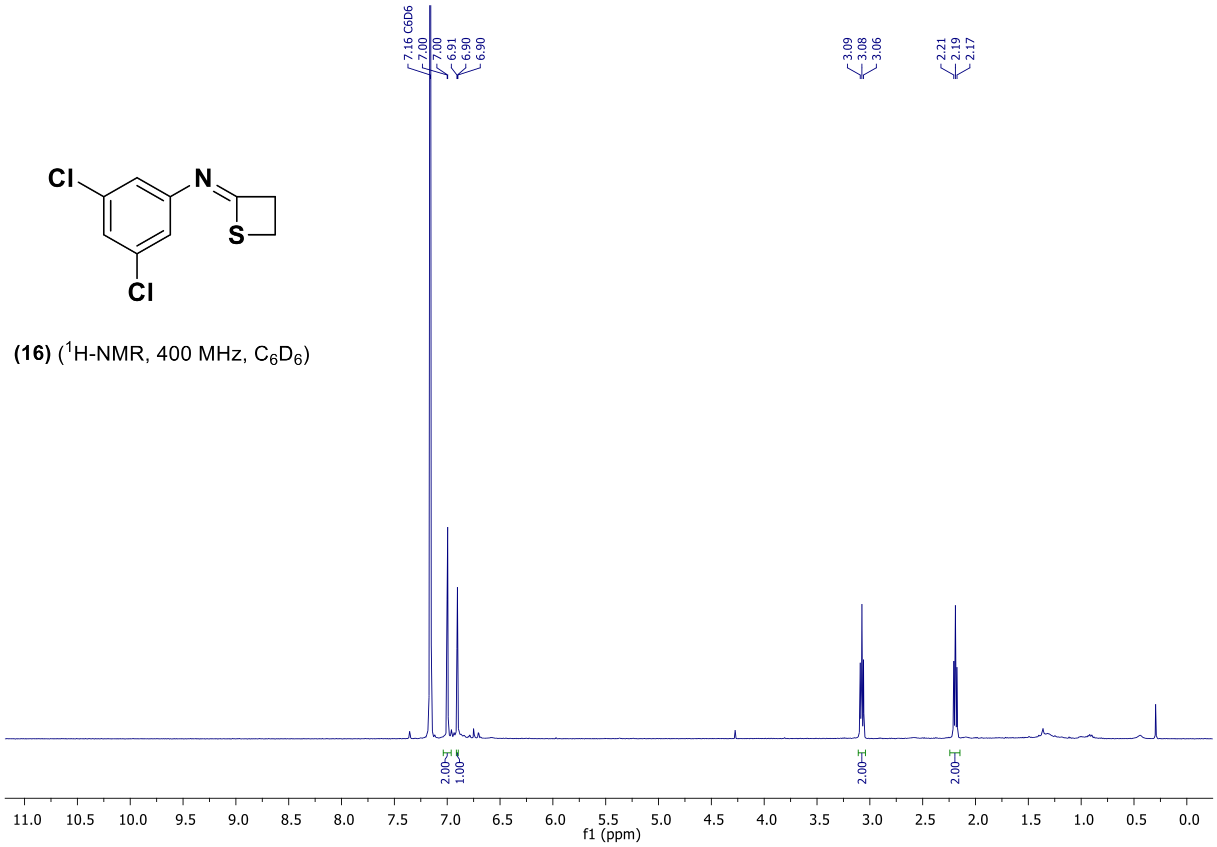

(16) (^{1}H-NMR, 400 MHz, C_6D_6)

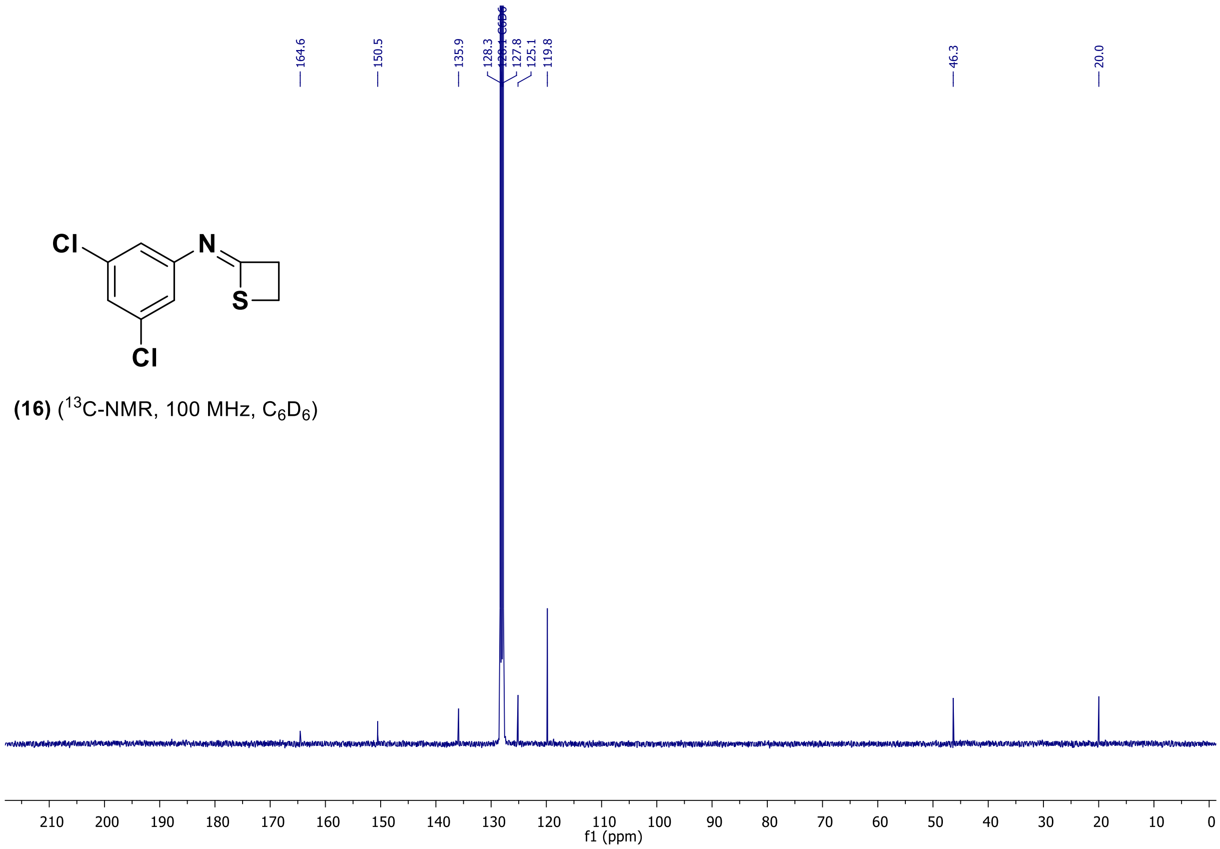

(16) (^{13}C-NMR, 100 MHz, C_6D_6)

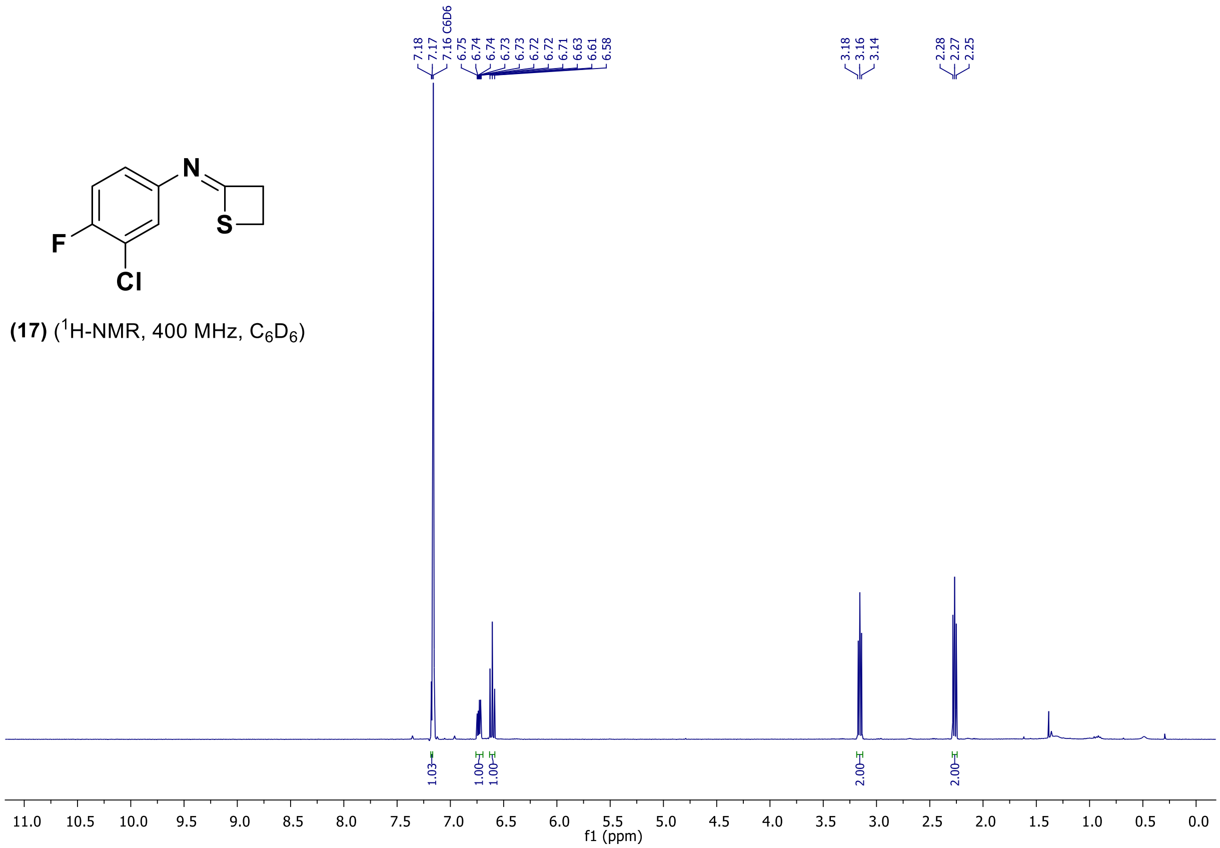

(17) (^{1}H-NMR, 400 MHz, C_6D_6)

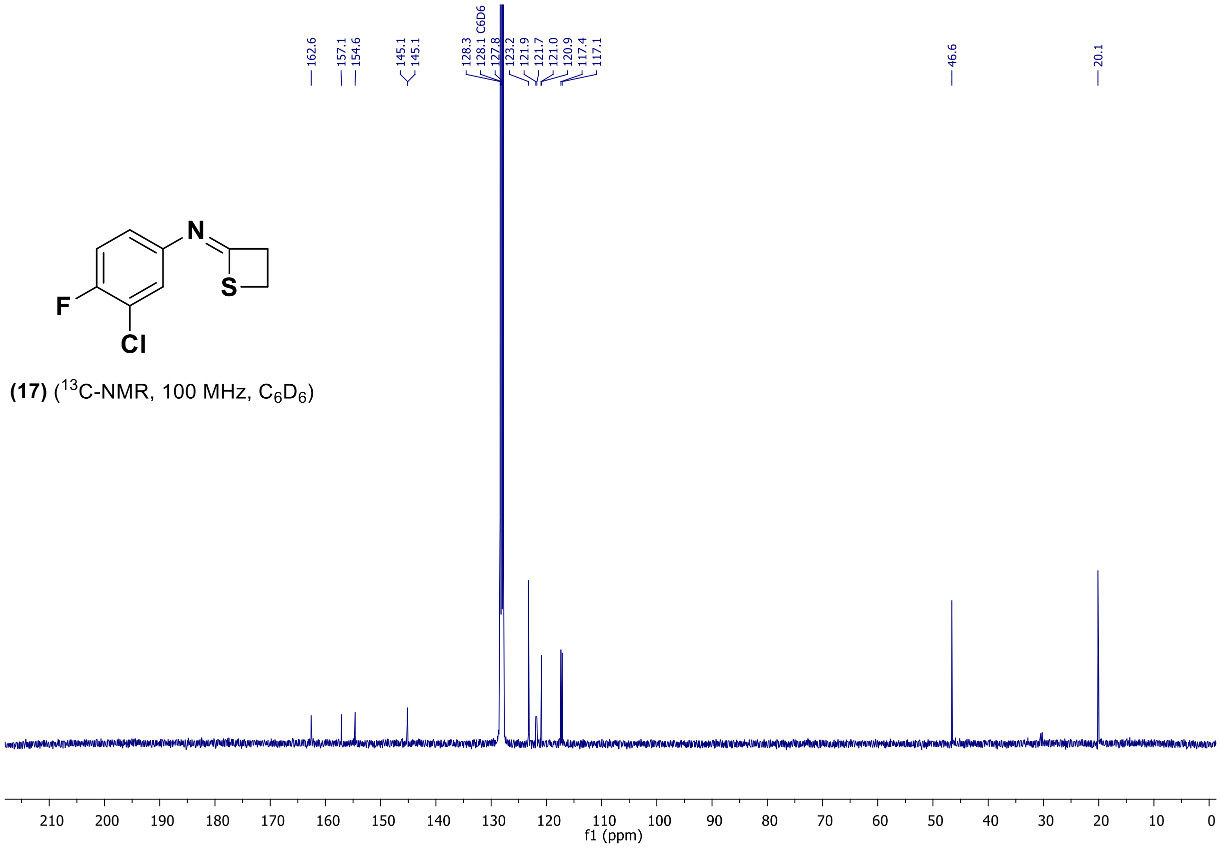

(17) (^{13}C-NMR, 100 MHz, C_6D_6)

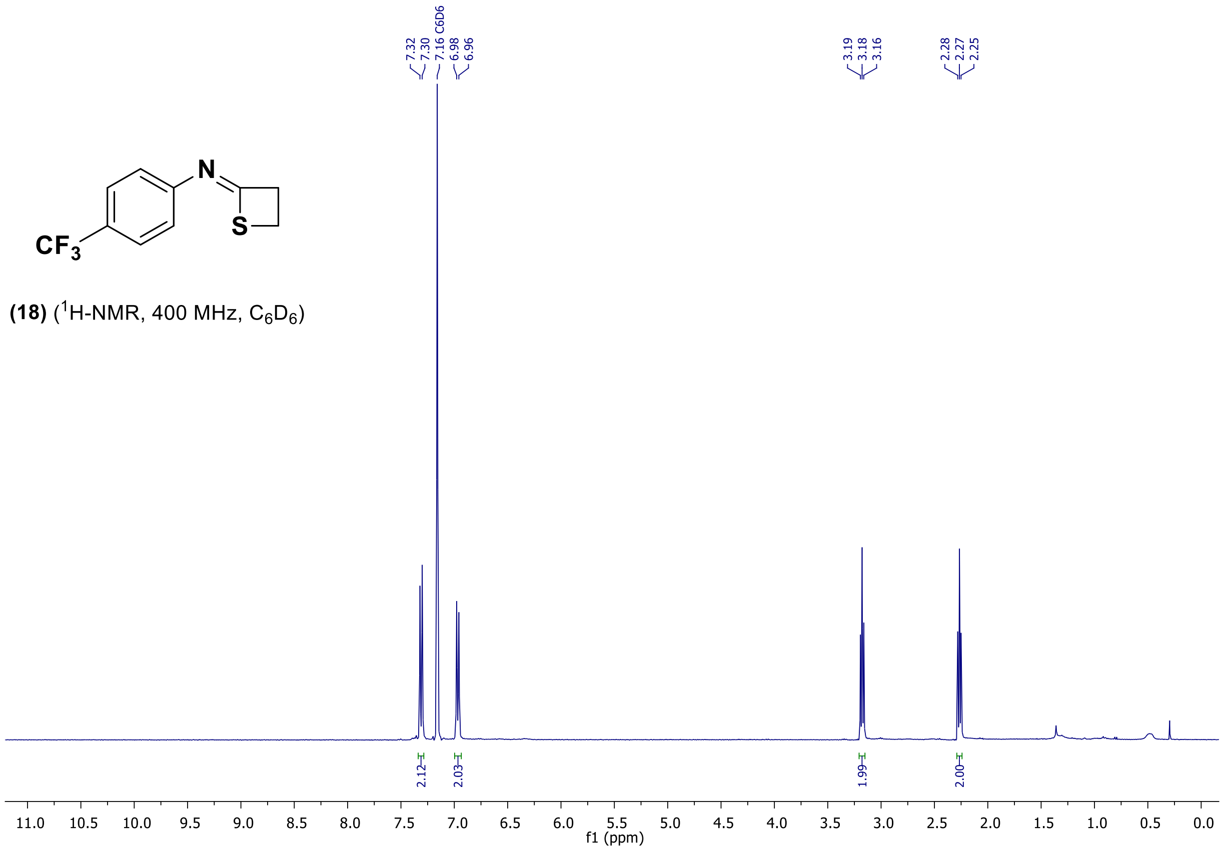

(18) (^{1}H-NMR, 400 MHz, C_6D_6)

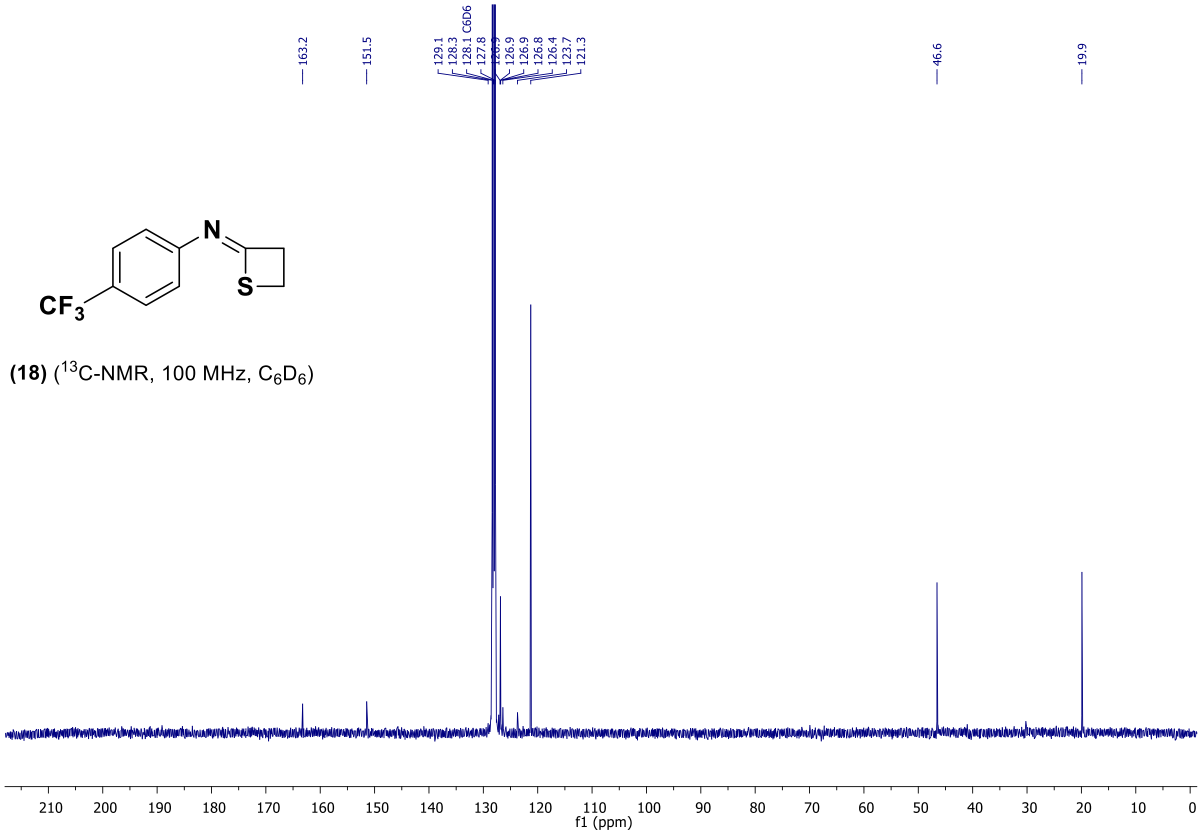

(18) (^{13}C-NMR, 100 MHz, C_6D_6)

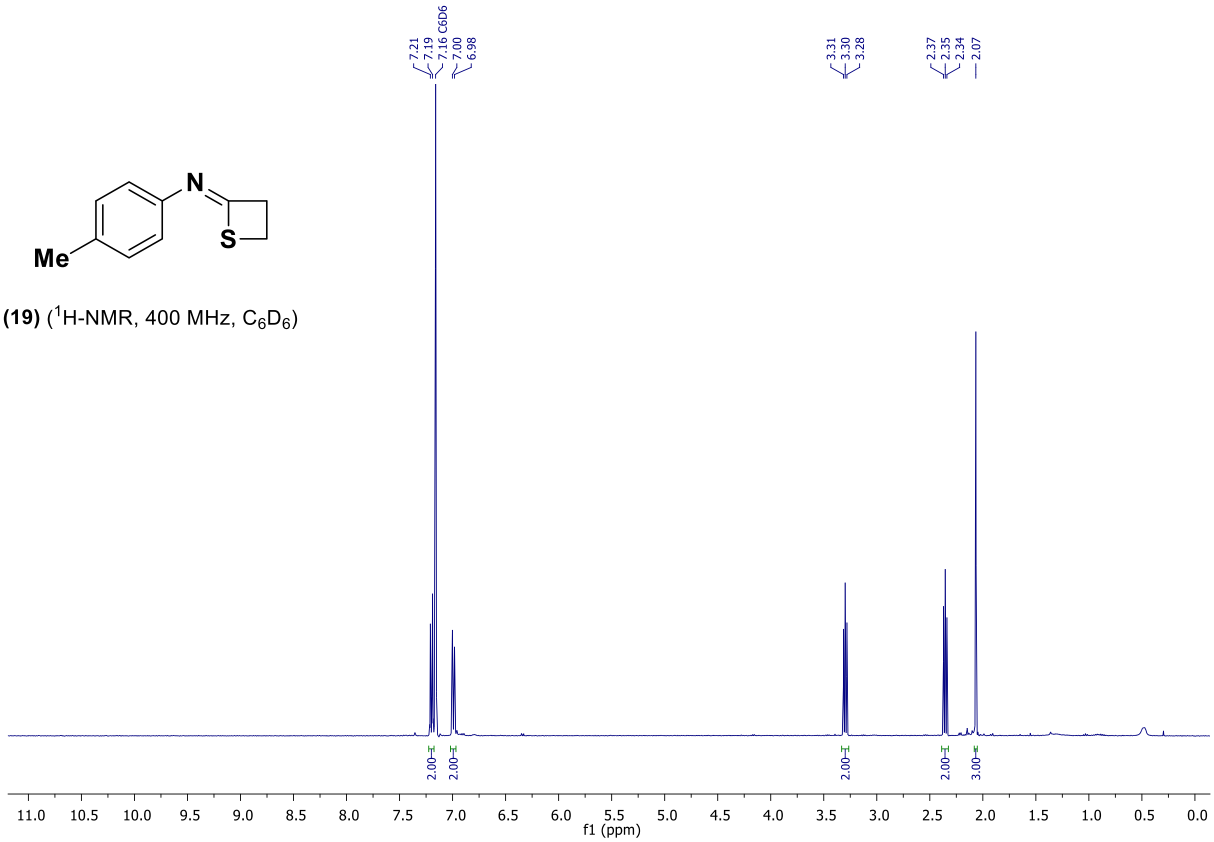

(19) (^{1}H-NMR, 400 MHz, C_6D_6)

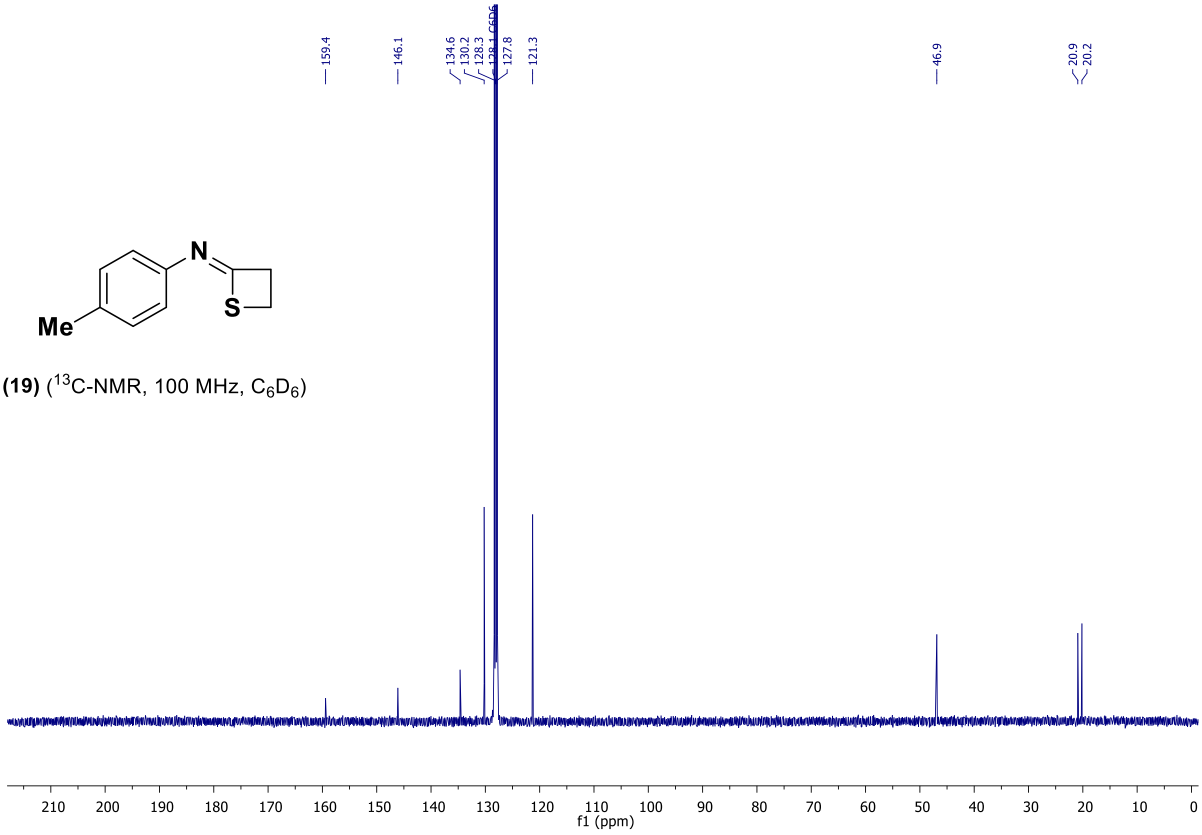

(19) (^{13}C-NMR, 100 MHz, C_6D_6)

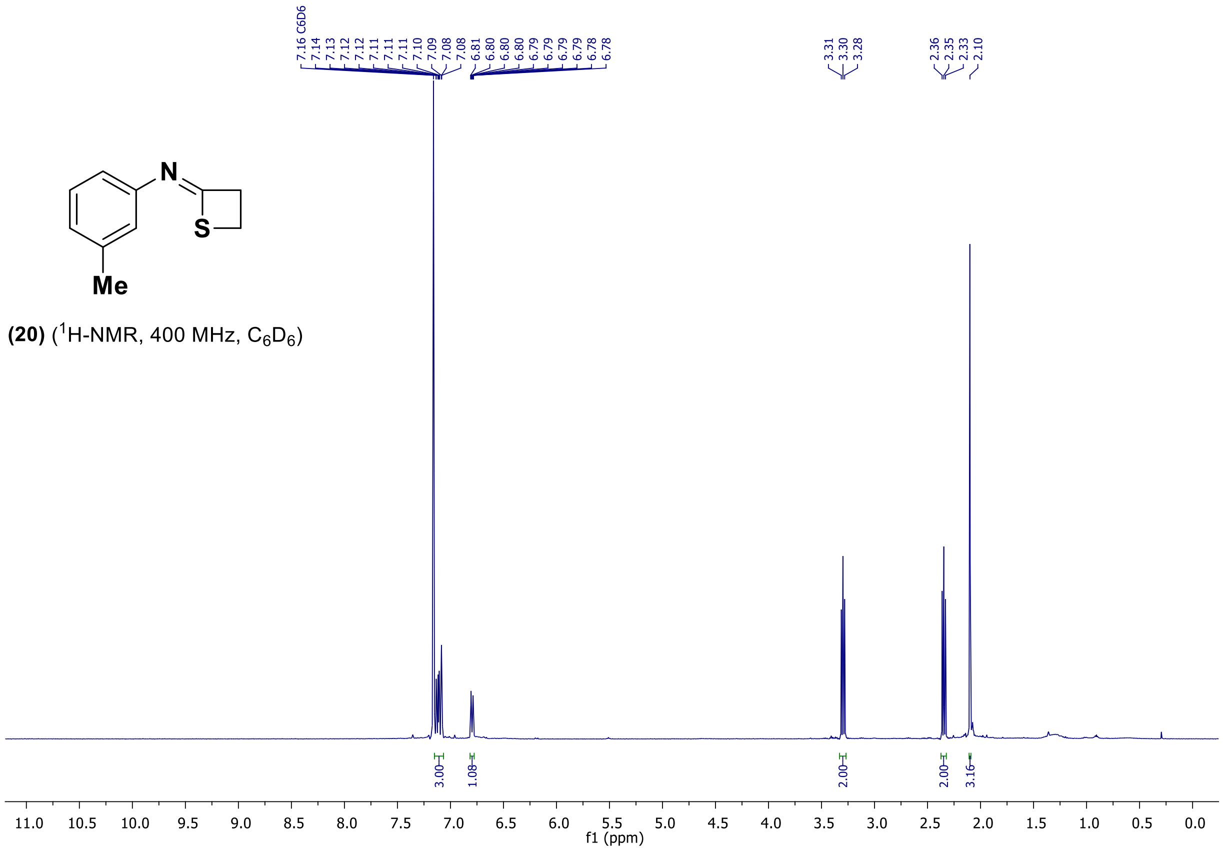

(20) (^{1}H-NMR, 400 MHz, C_6D_6)

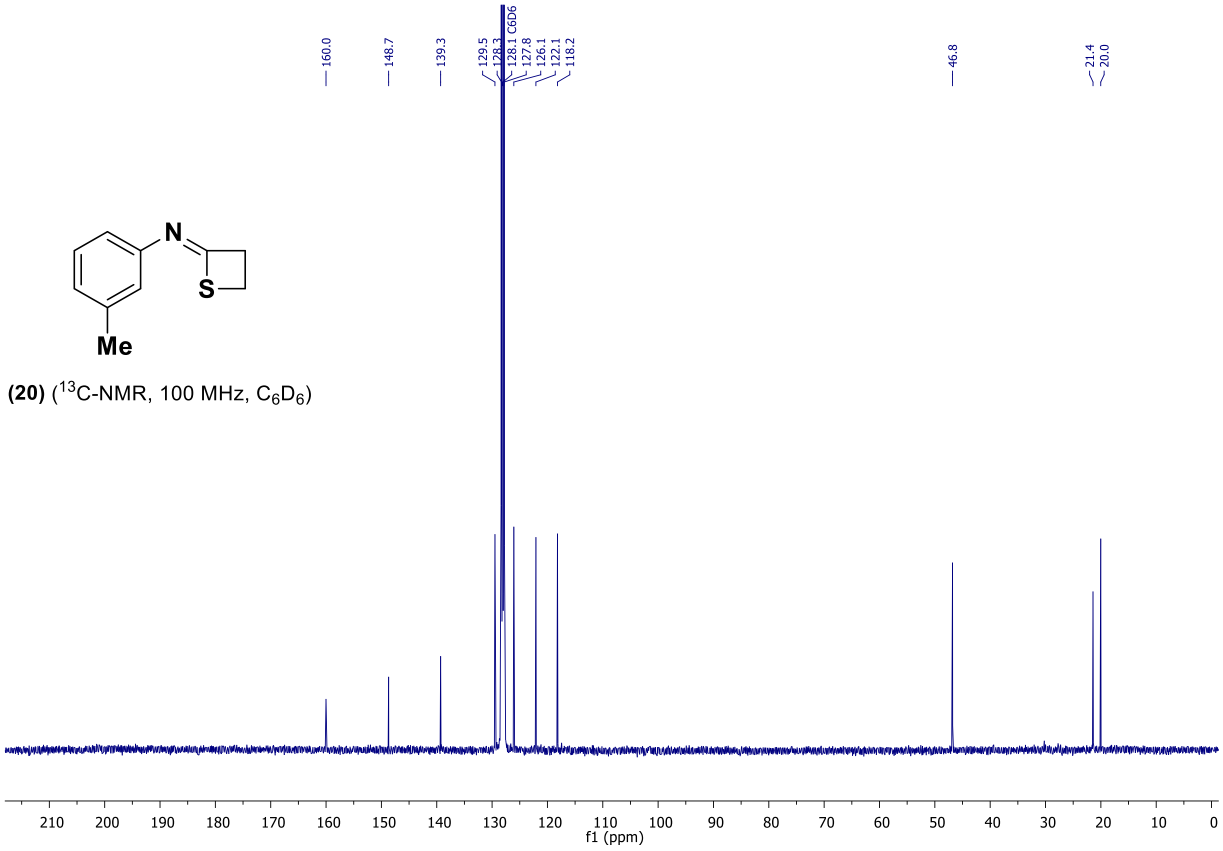

(20) (^{13}C-NMR, 100 MHz, C_6D_6)

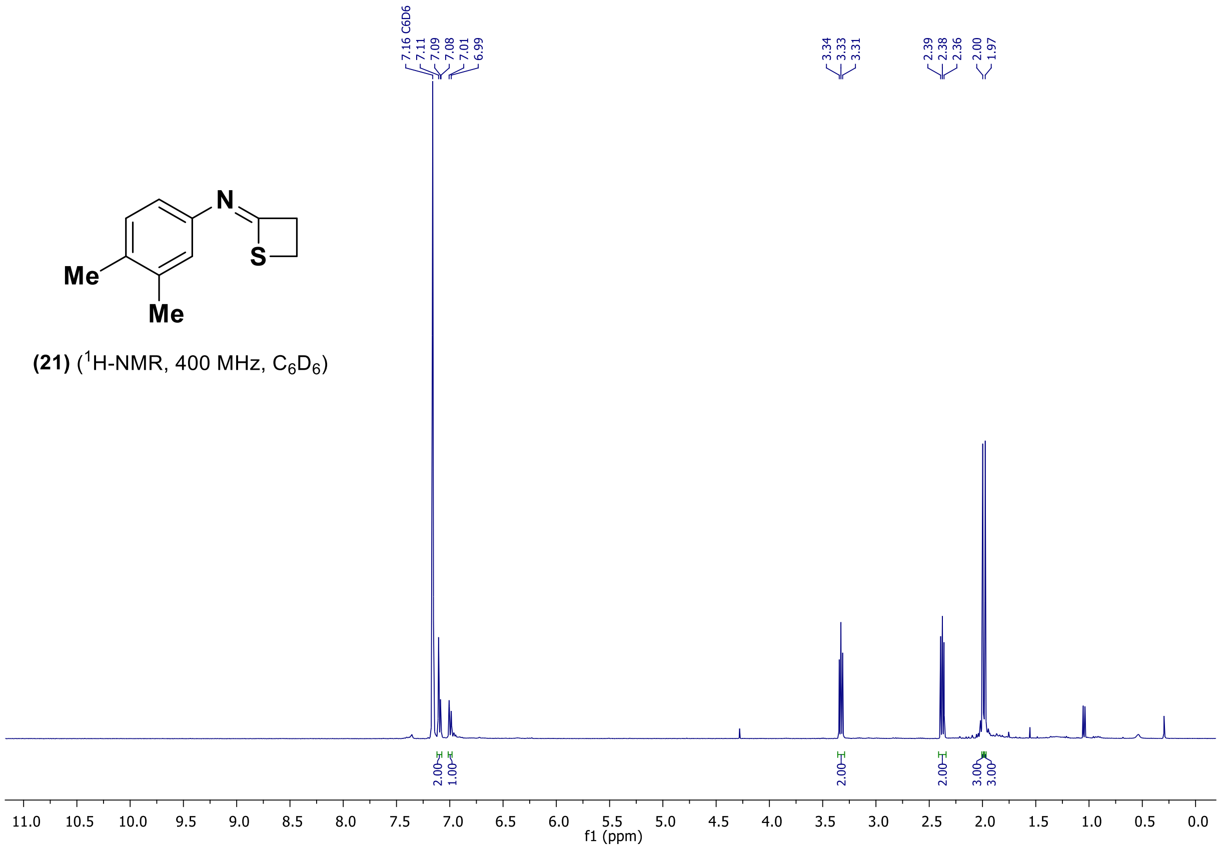

(21) (^{1}H-NMR, 400 MHz, C_6D_6)

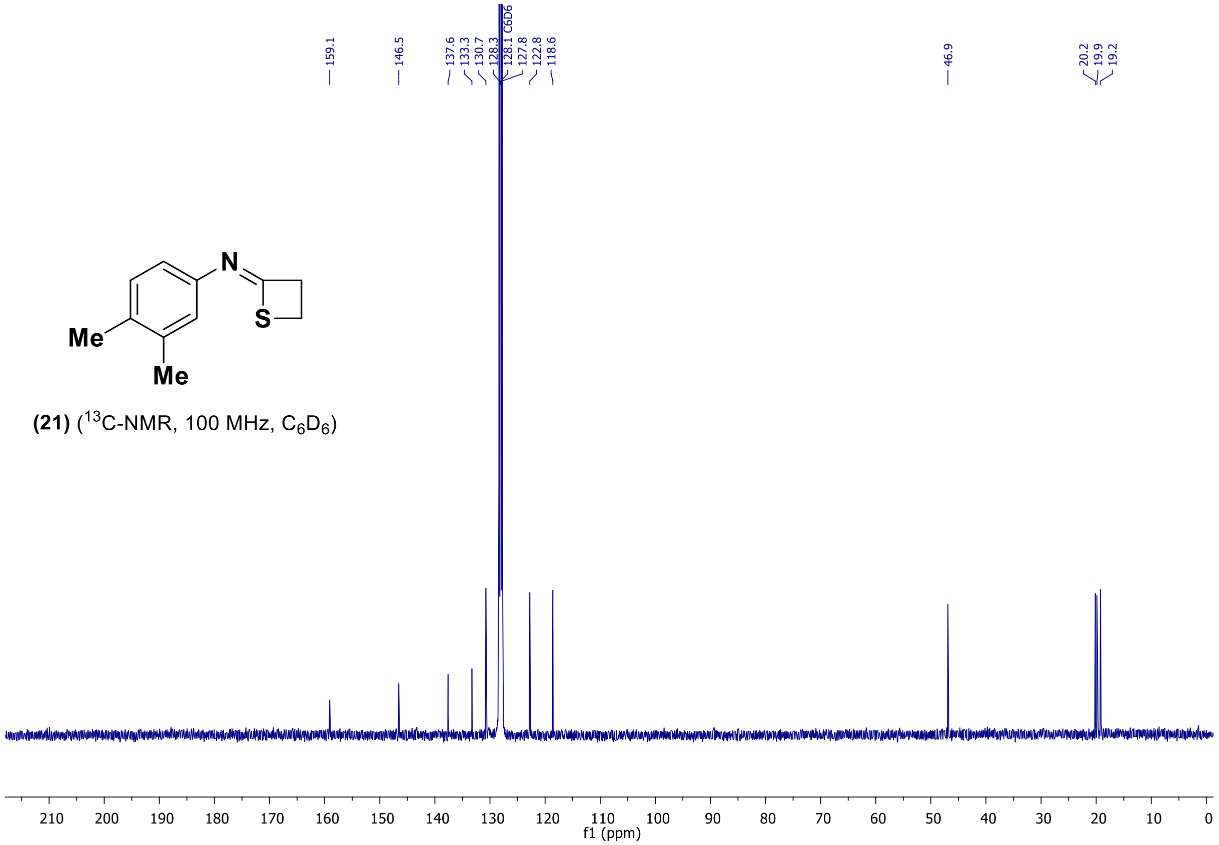

(21) (^{13}C-NMR, 100 MHz, C_6D_6)

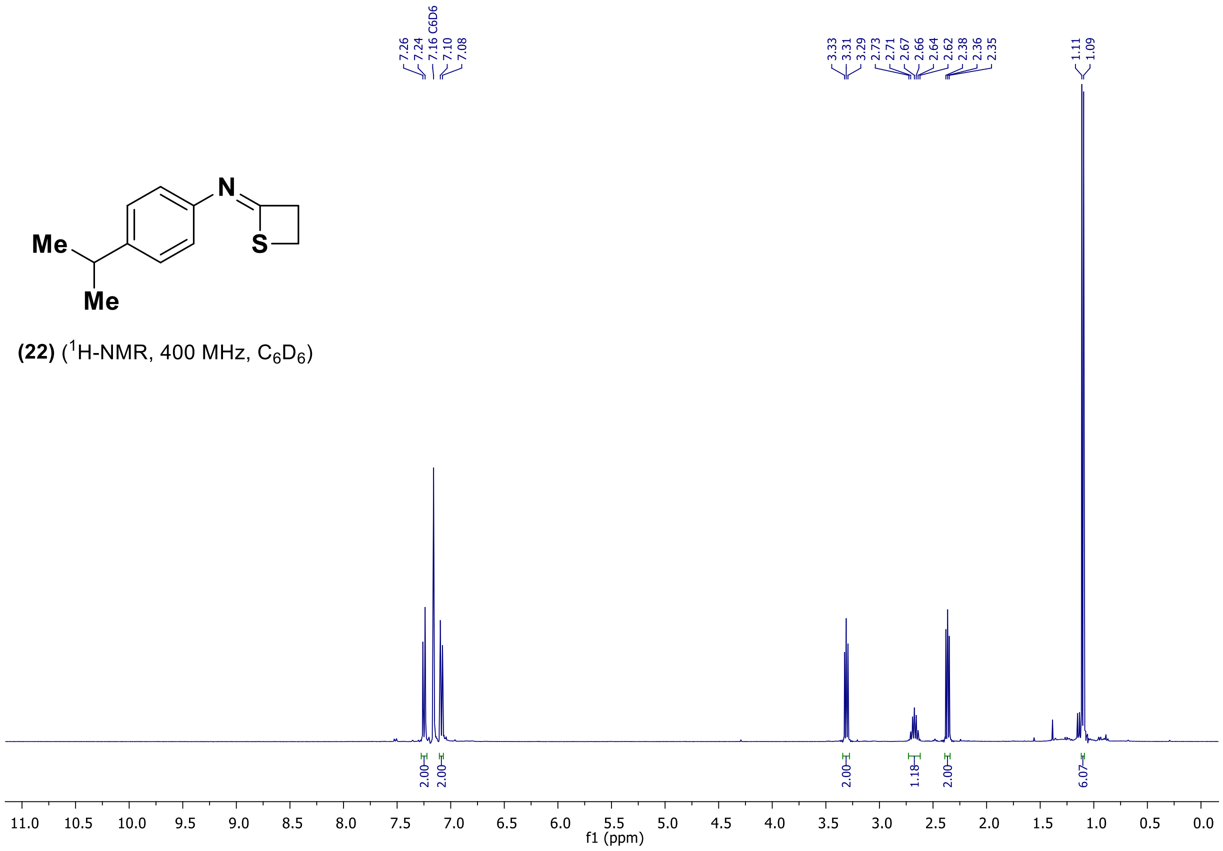

(22) (^{1}H-NMR, 400 MHz, C_6D_6)

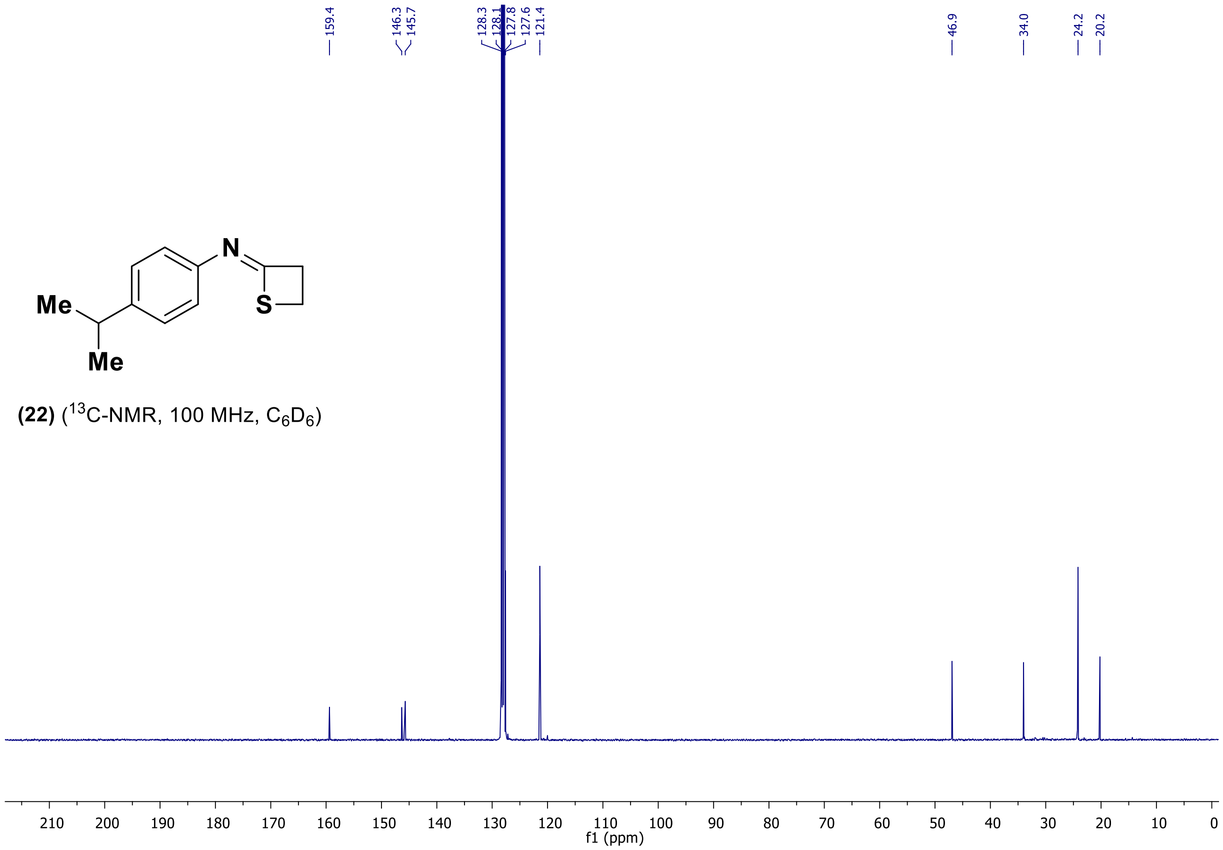

(22) (^{13}C-NMR, 100 MHz, C_6D_6)

(23) (^{1}H-NMR, 400 MHz, C_6D_6)

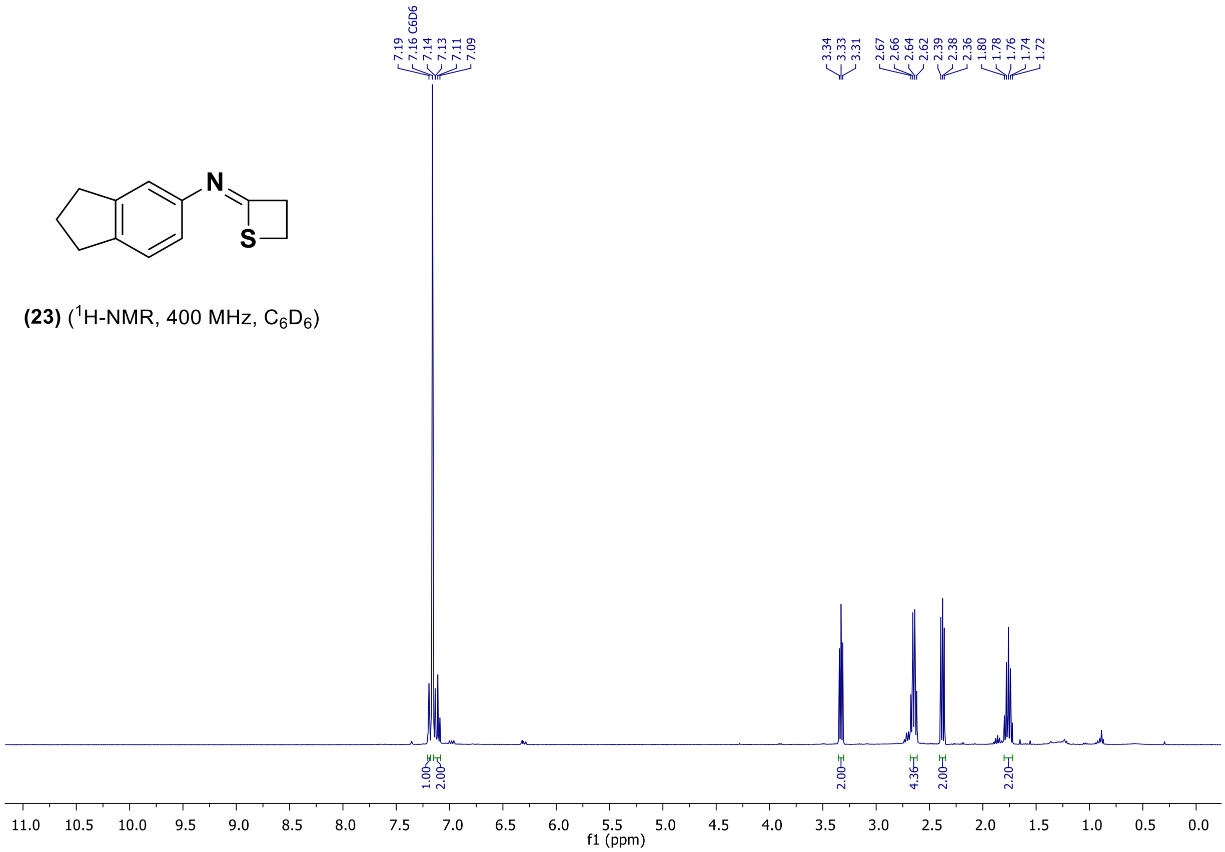

(23) (^{13}C-NMR, 100 MHz, C_6D_6)

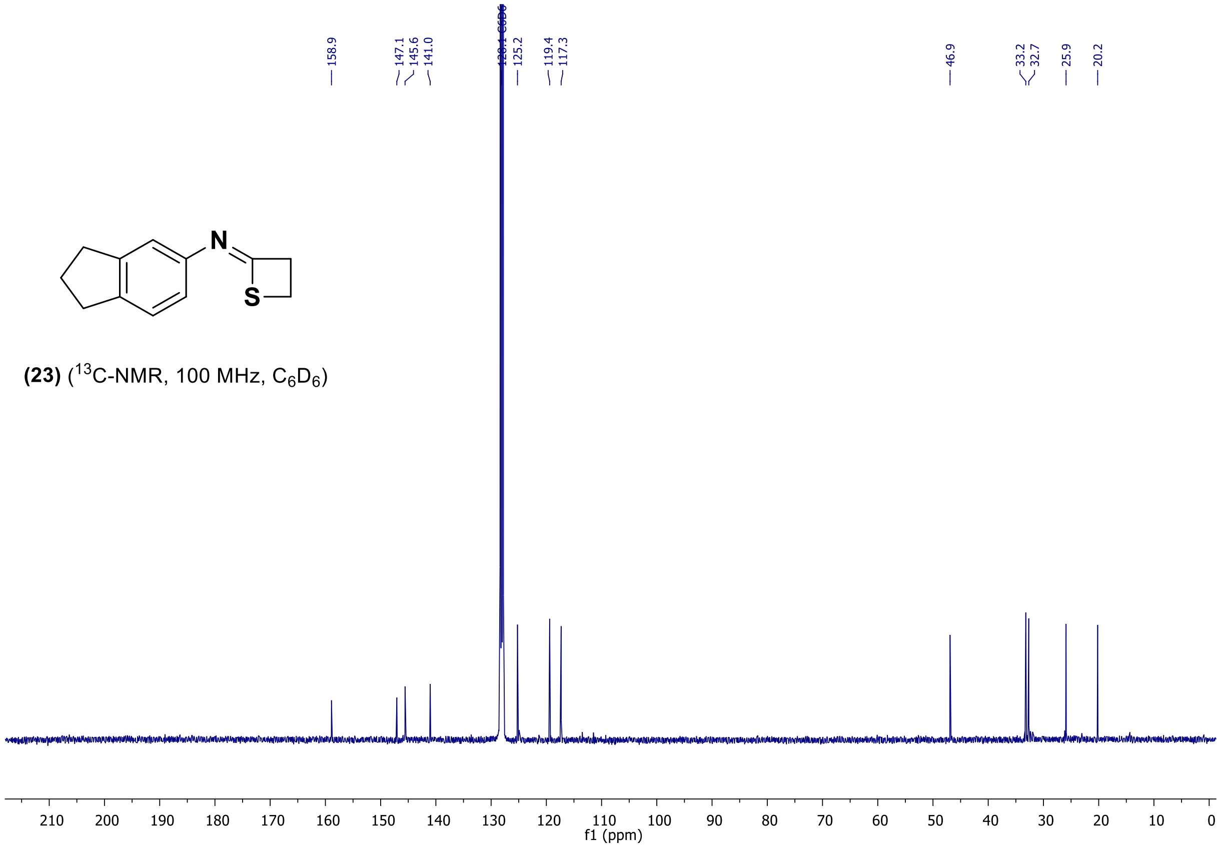

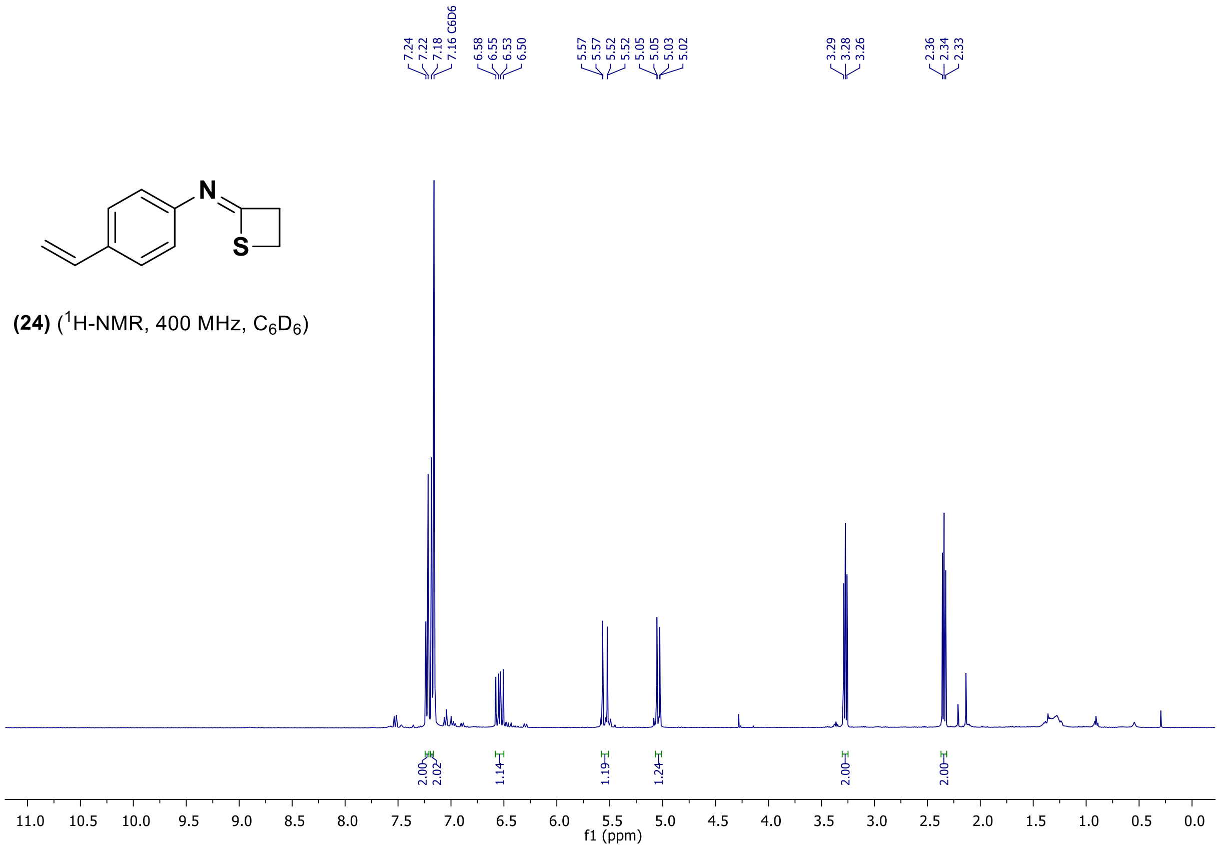

(24) (^{1}H-NMR, 400 MHz, C_6D_6)

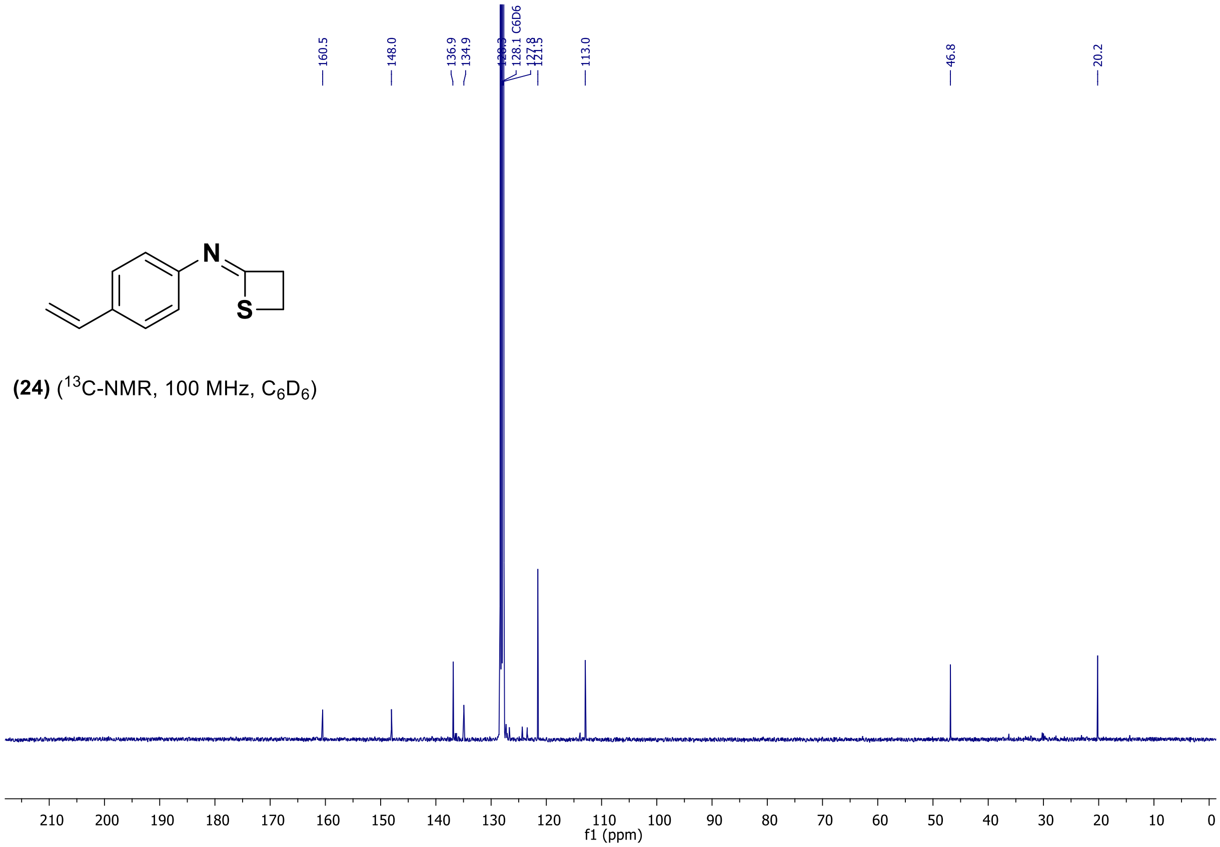

(24) (^{13}C-NMR, 100 MHz, C_6D_6)

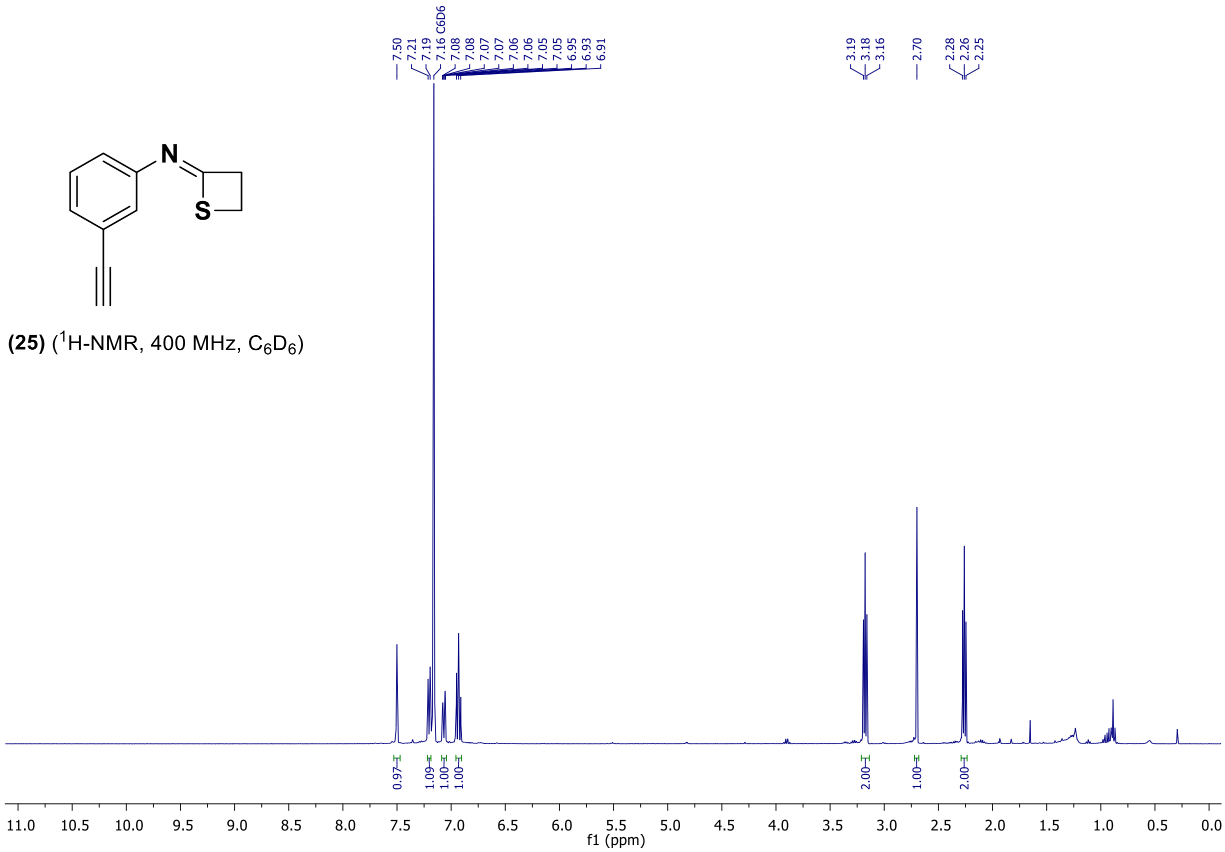

(25) (^{1}H-NMR, 400 MHz, C_6D_6)

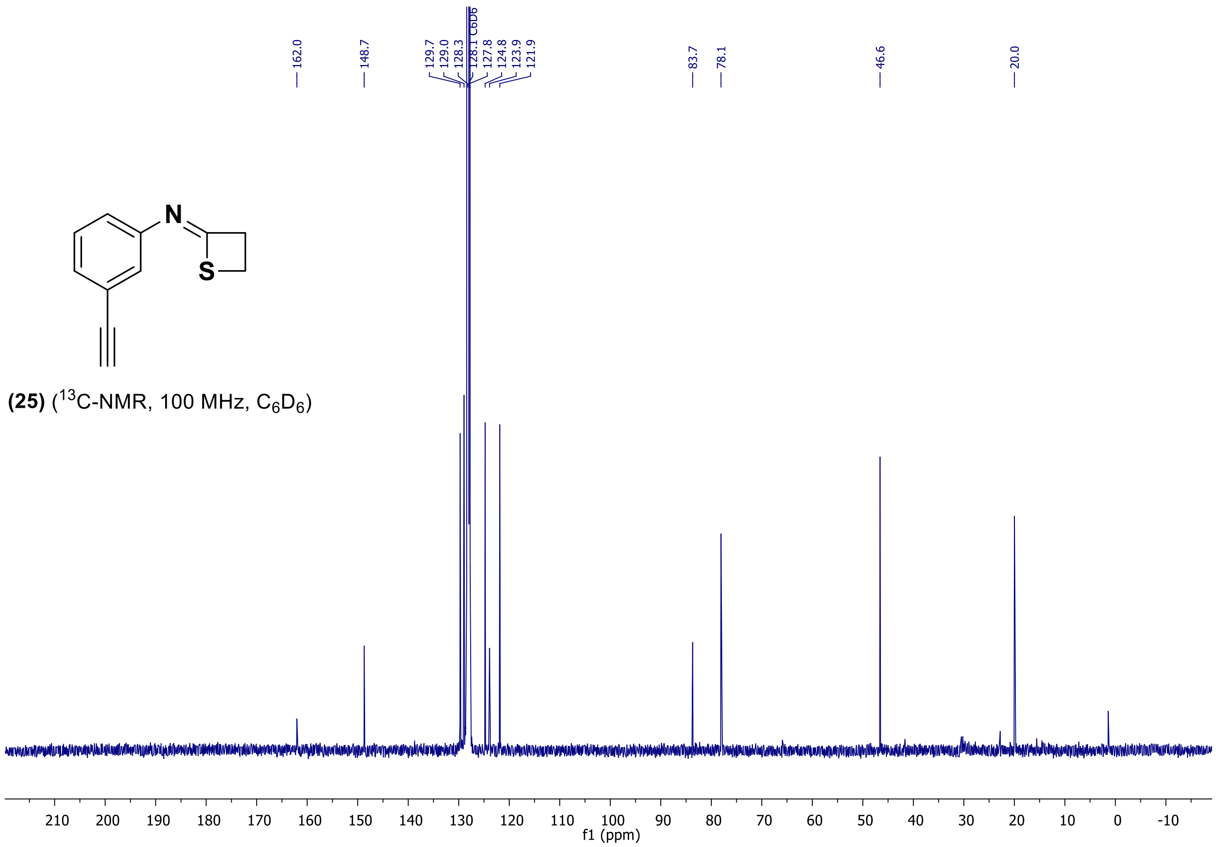

(25) (^{13}C-NMR, 100 MHz, C_6D_6)

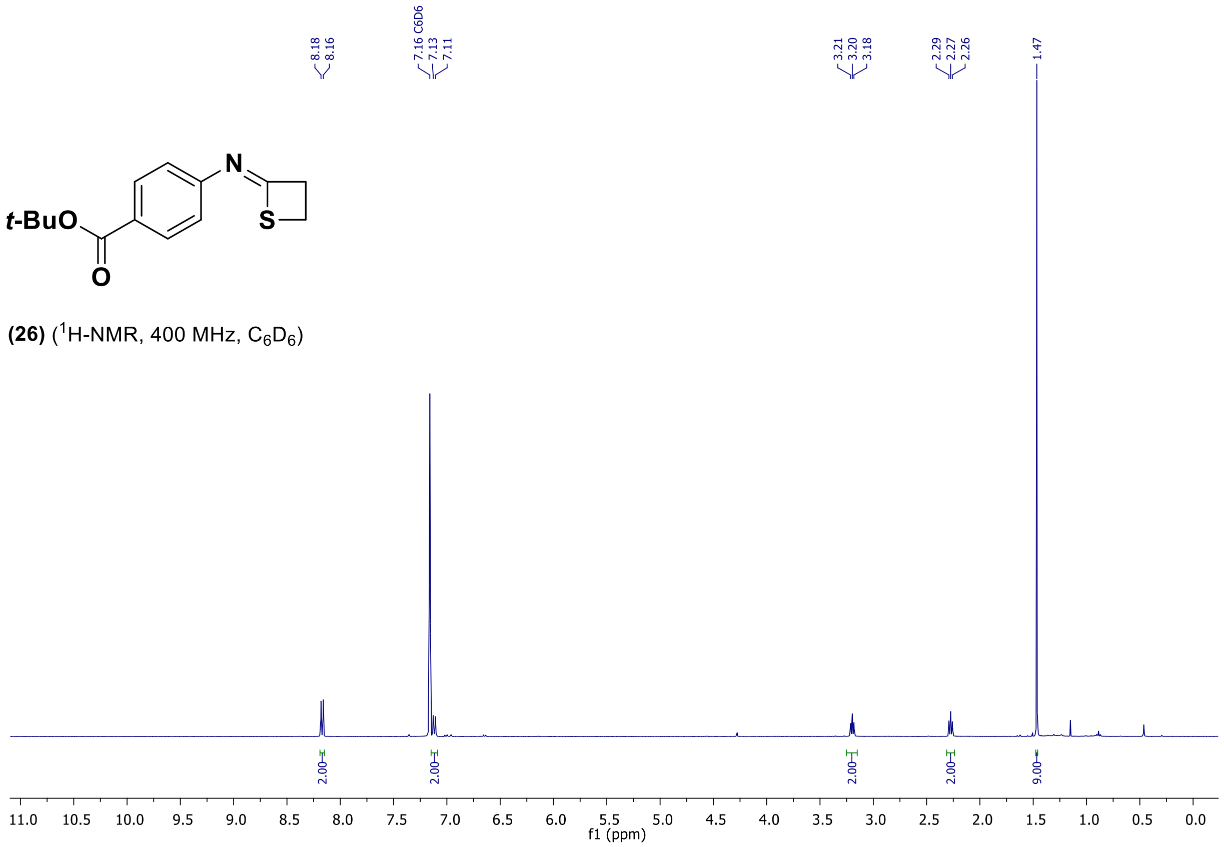

(26) (^{1}H-NMR, 400 MHz, C_6D_6)

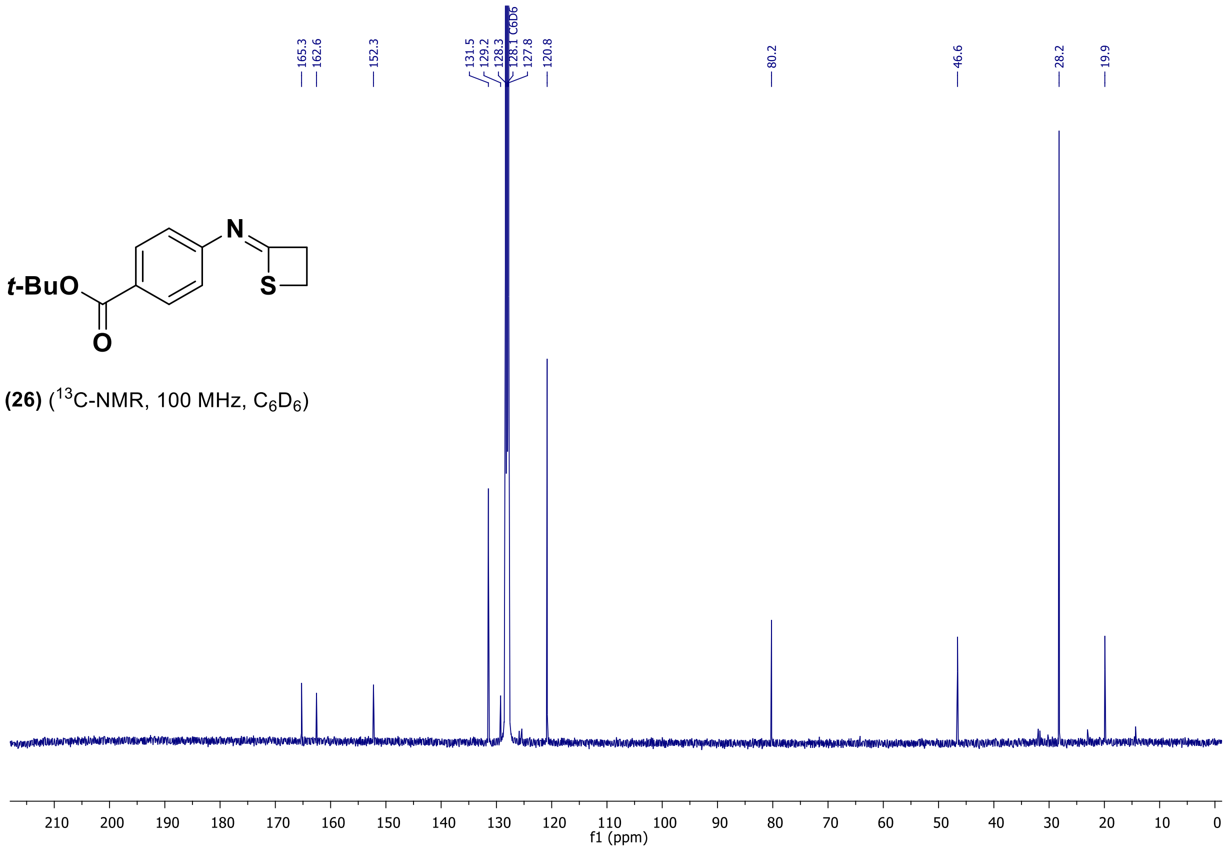

(26) (^{13}C-NMR, 100 MHz, C_6D_6)

(27) (^{1}H-NMR, 400 MHz, C_6D_6)

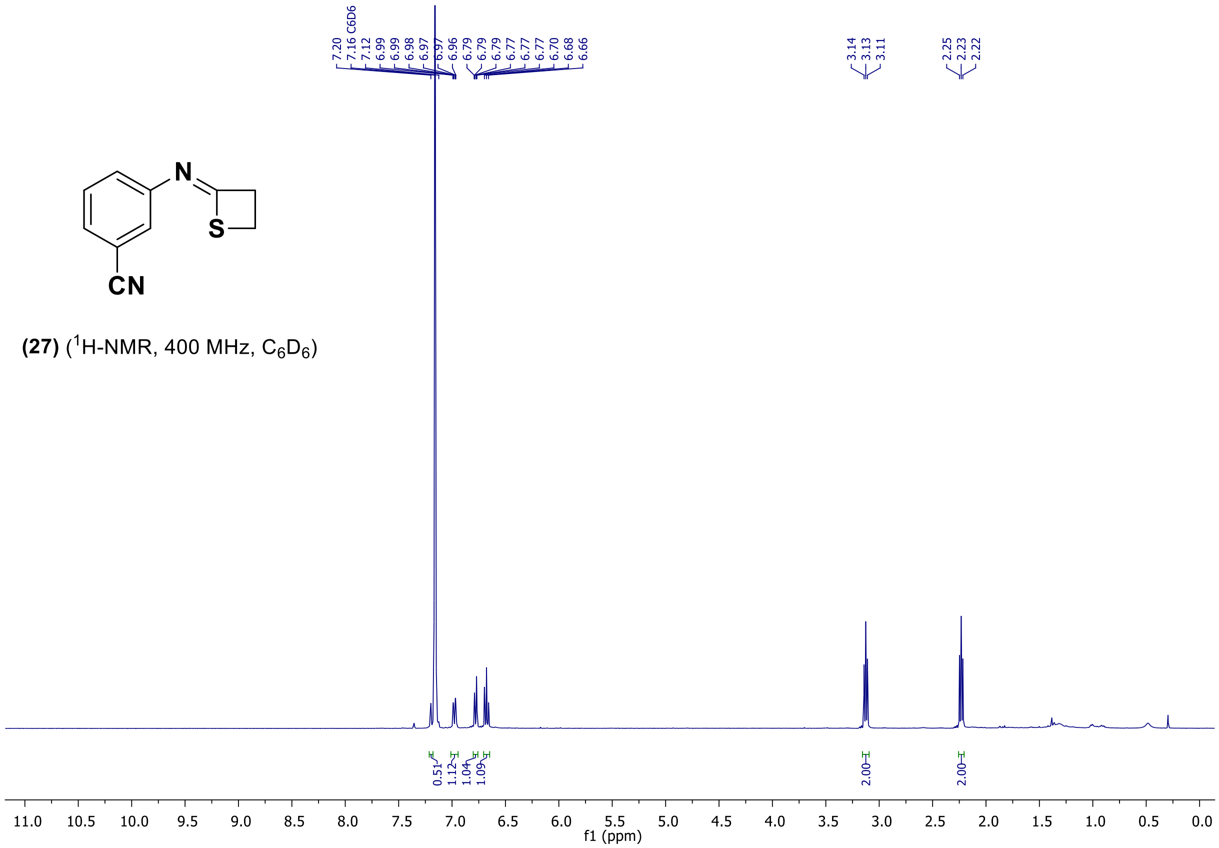

(27) (^{13}C-NMR, 100 MHz, C_6D_6)

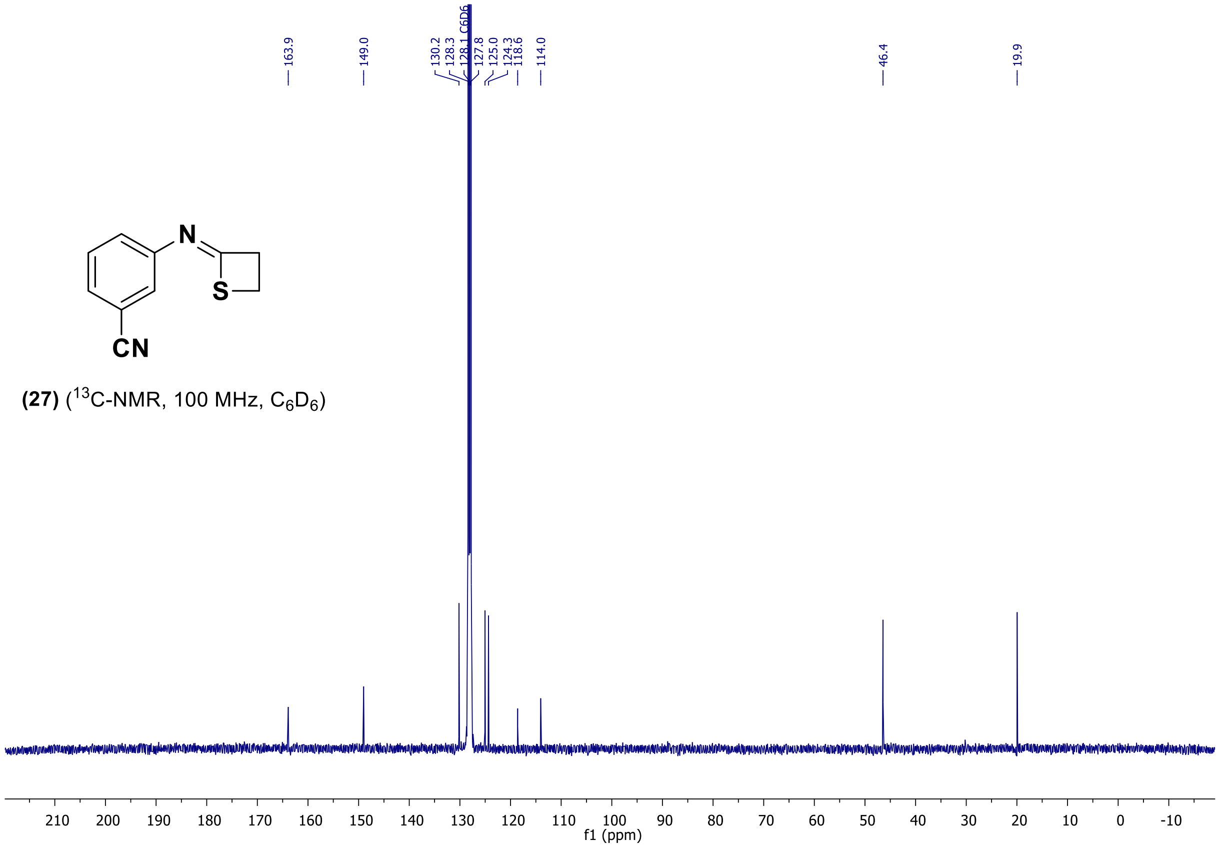

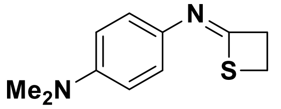

(28) (^{1}H-NMR, 400 MHz, C_6D_6)

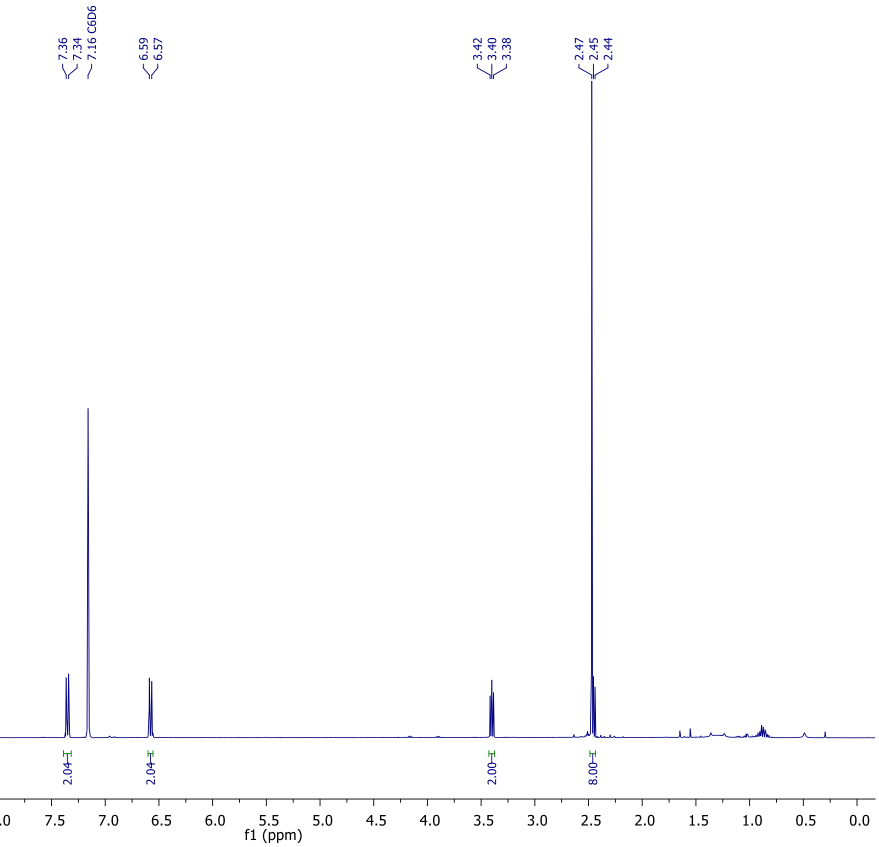

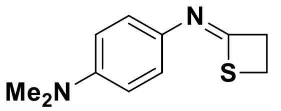

(28) (^{13}C-NMR, 100 MHz, C_6D_6)

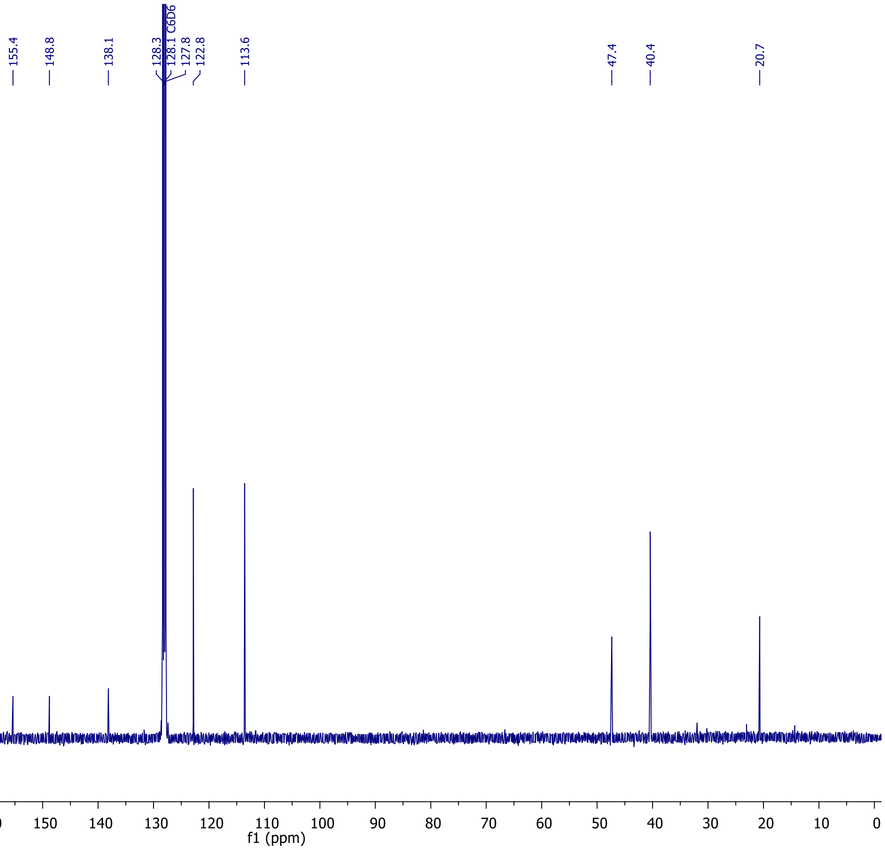

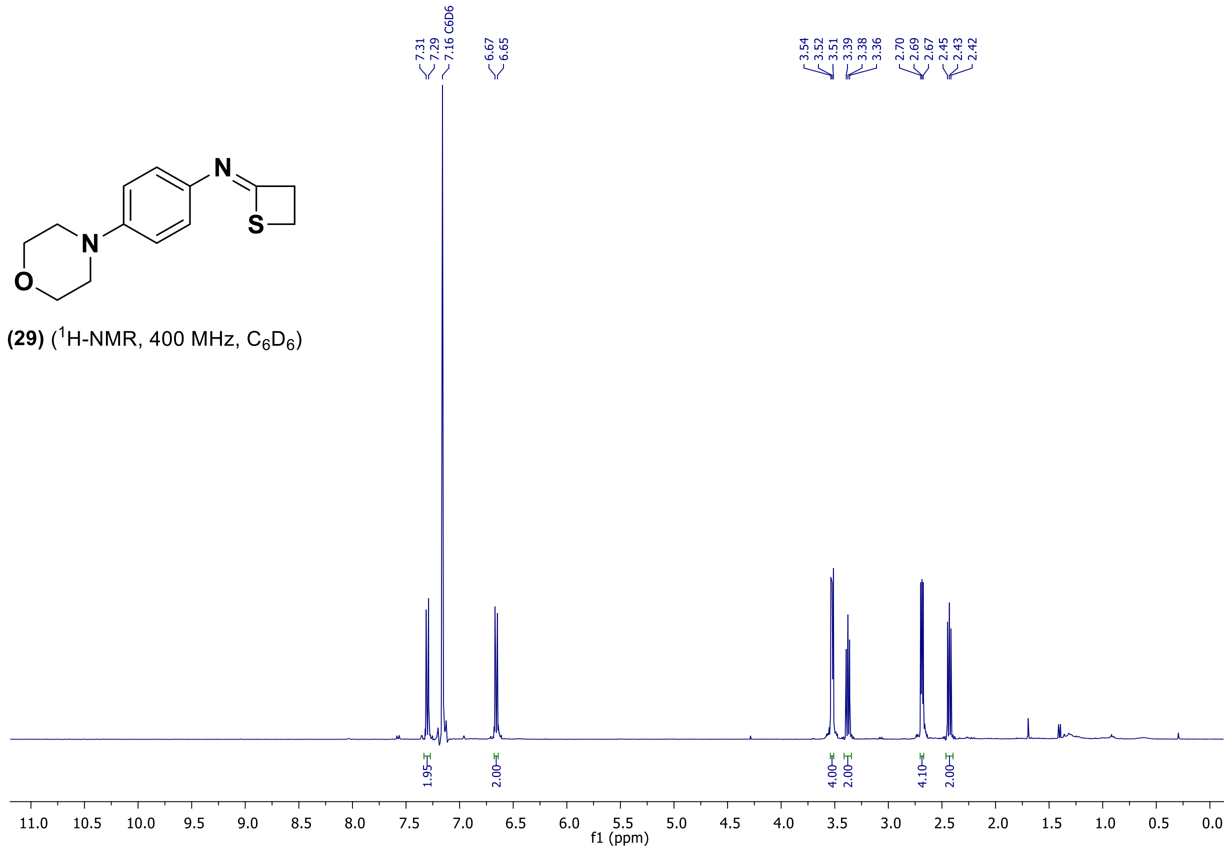

(29) (^{1}H-NMR, 400 MHz, C_6D_6)

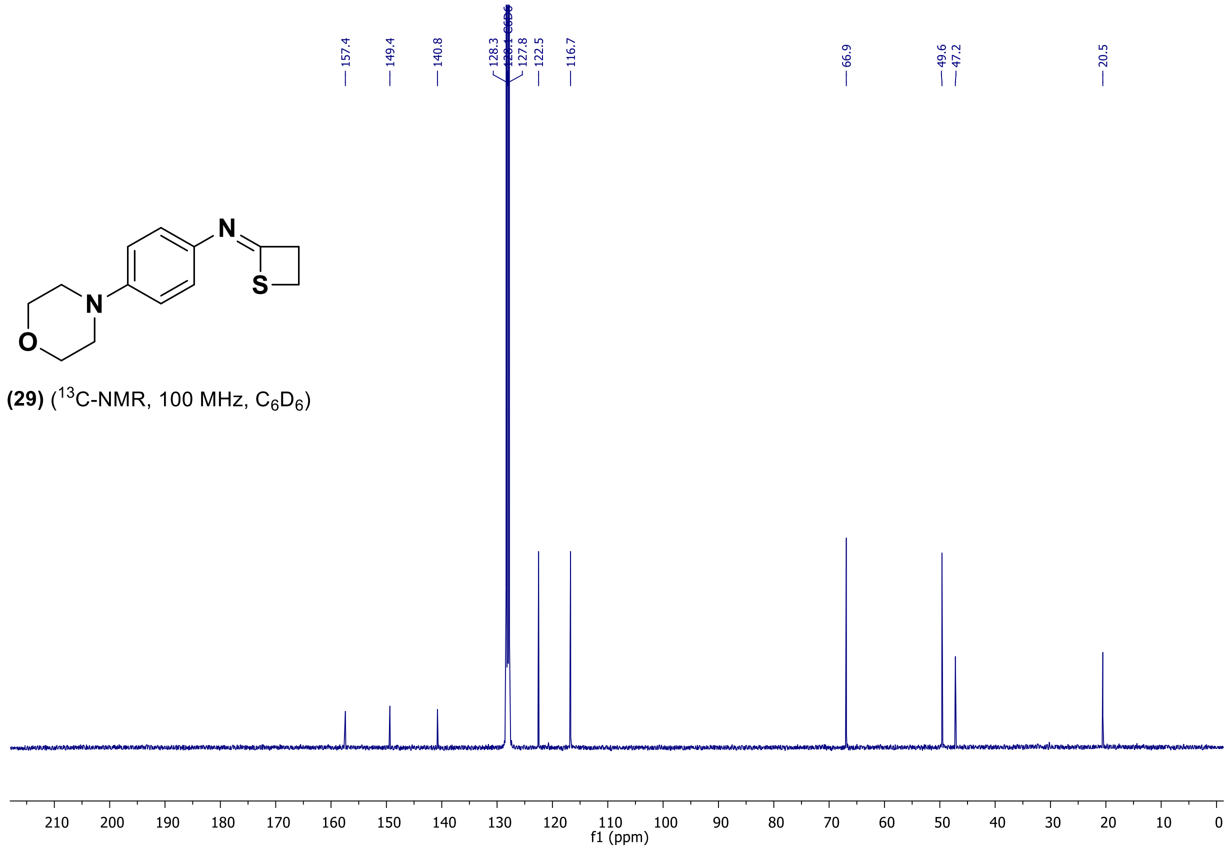

(29) (^{13}C-NMR, 100 MHz, C_6D_6)

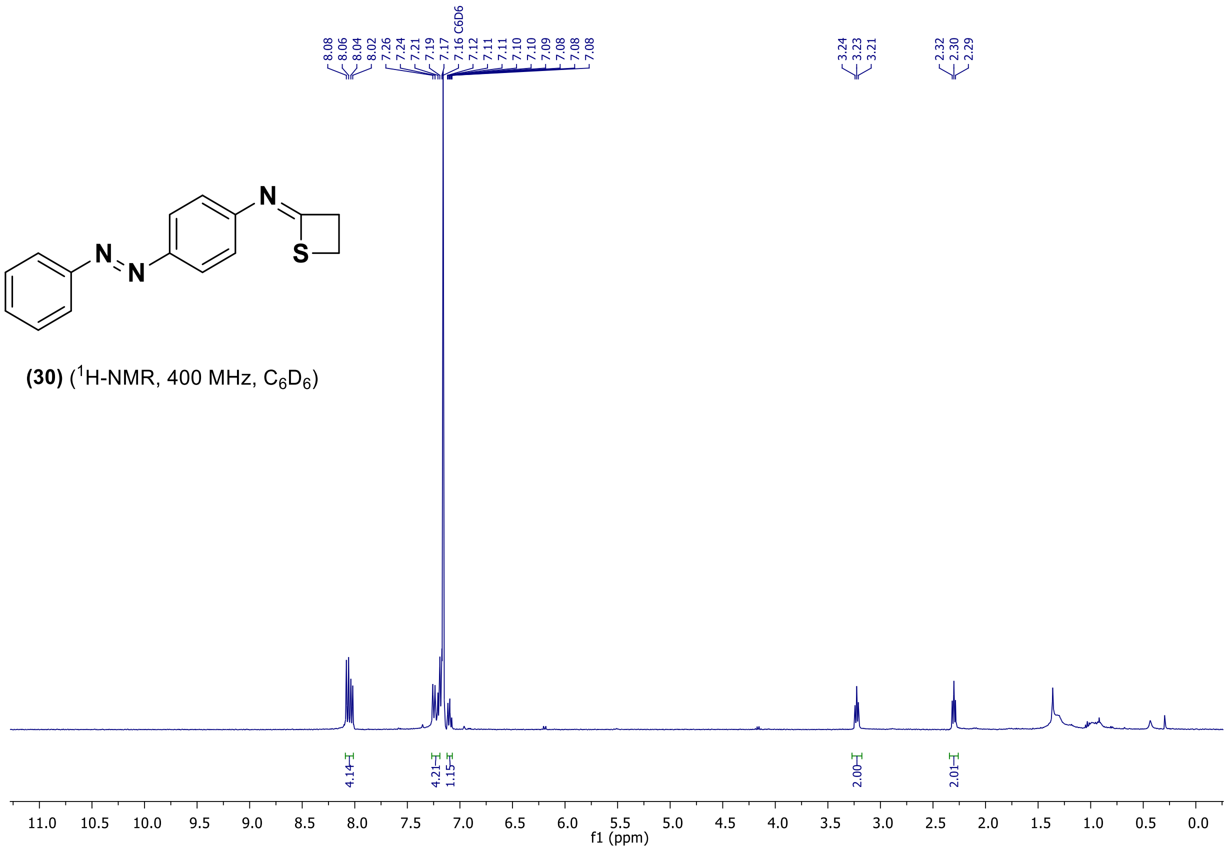

(30) (^{1}H-NMR, 400 MHz, C_6D_6)

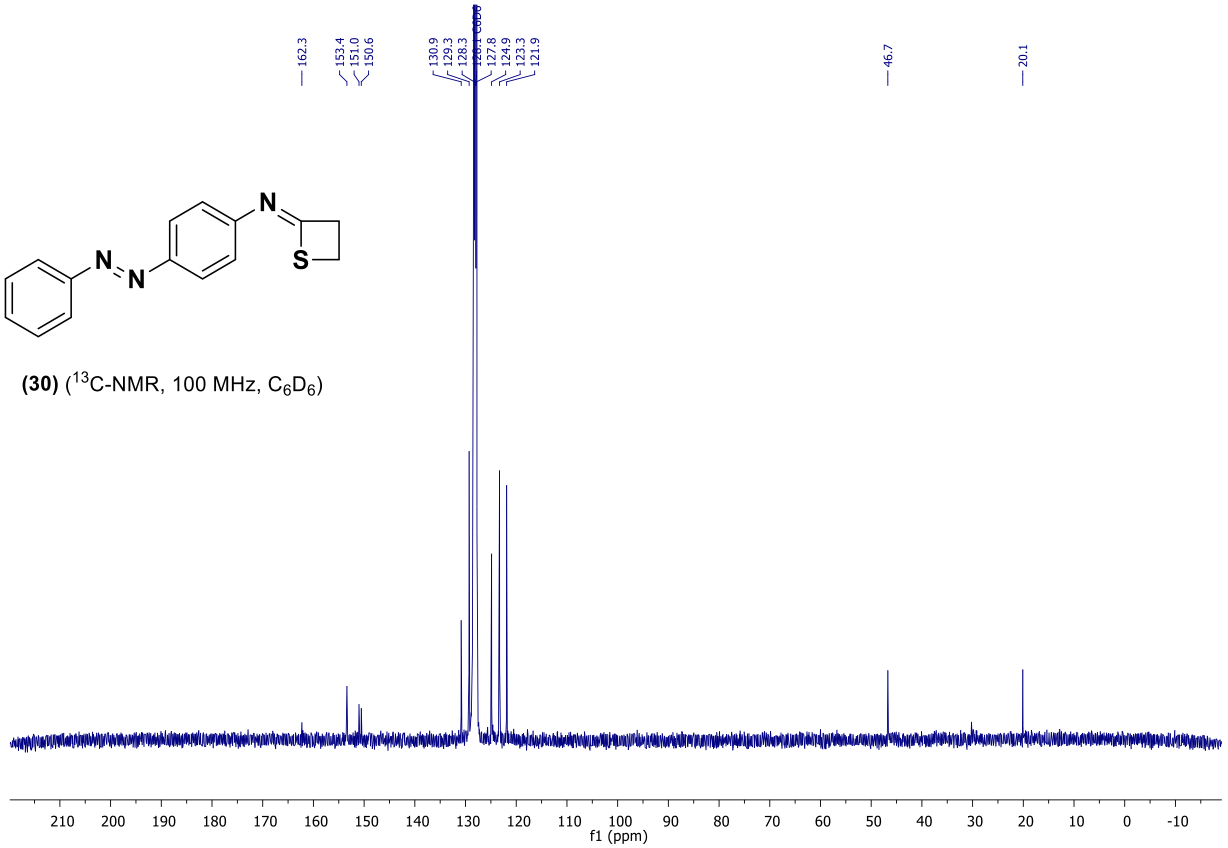

(30) (^{13}C-NMR, 100 MHz, C_6D_6)

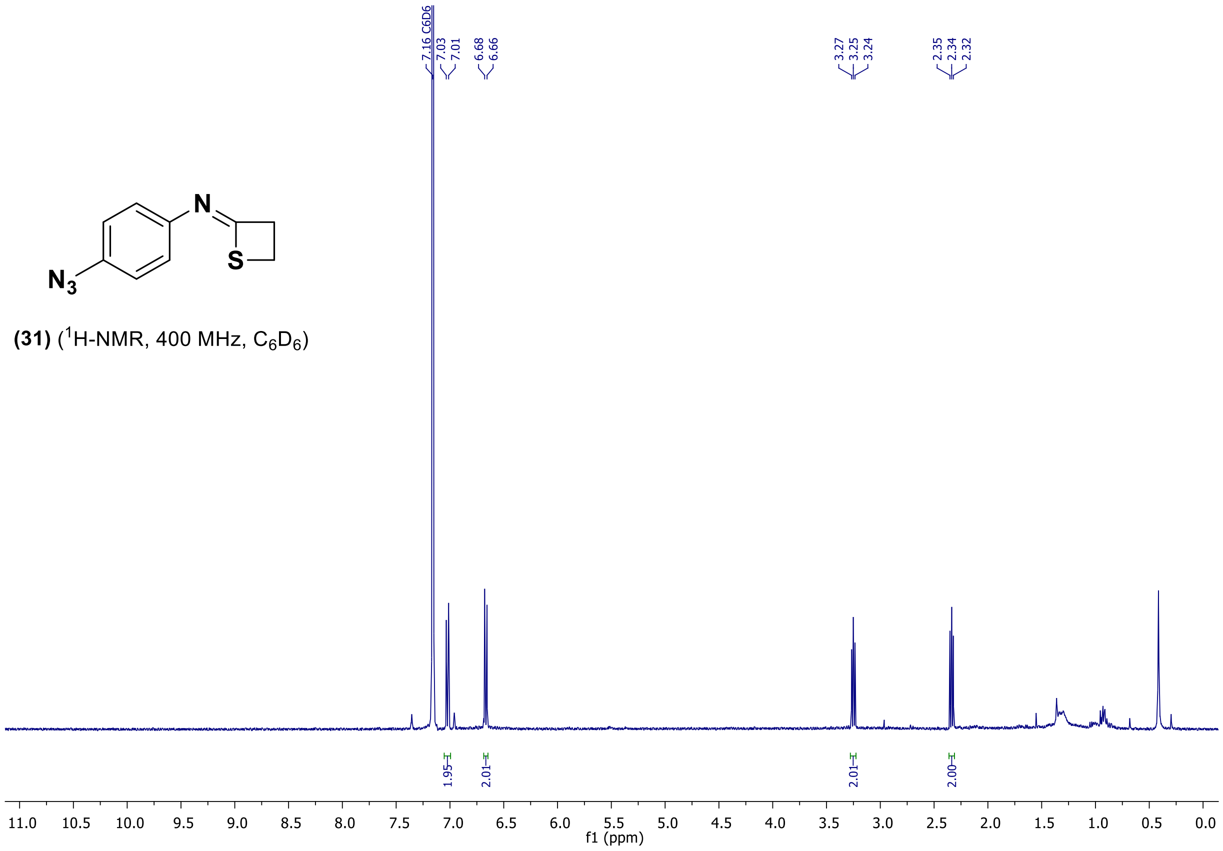

(31) (^{1}H-NMR, 400 MHz, C_6D_6)

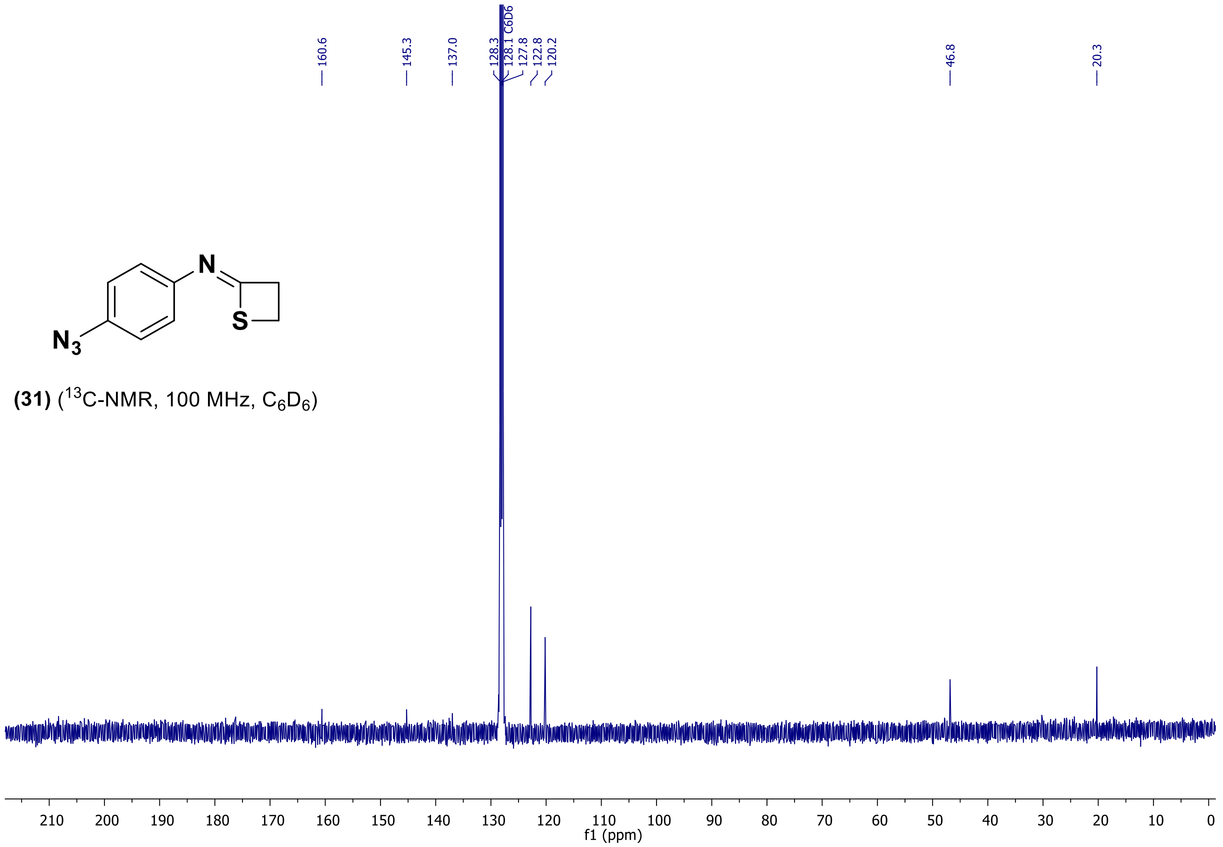

(31) (^{13}C-NMR, 100 MHz, C_6D_6)

(32) (^{1}H-NMR, 400 MHz, C_6D_6)

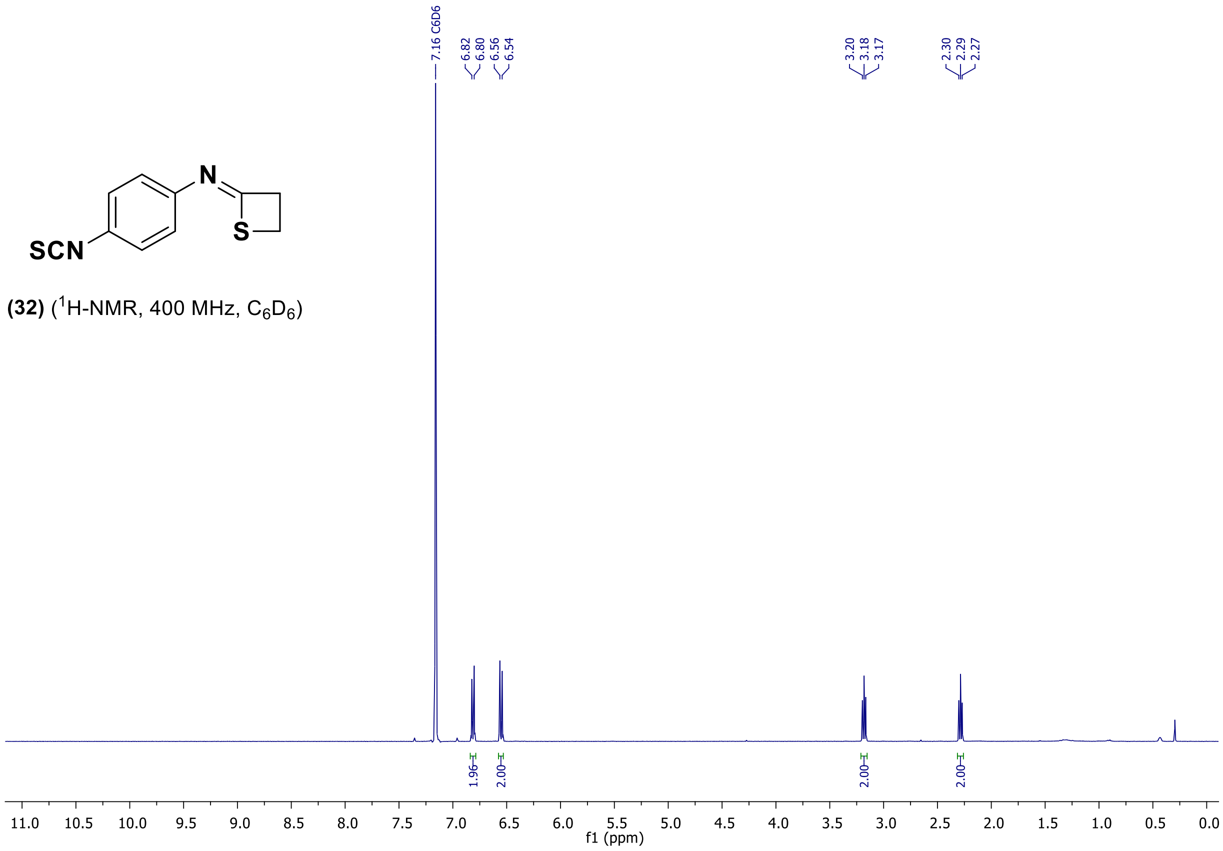

(32) (^{13}C-NMR, 100 MHz, C_6D_6)

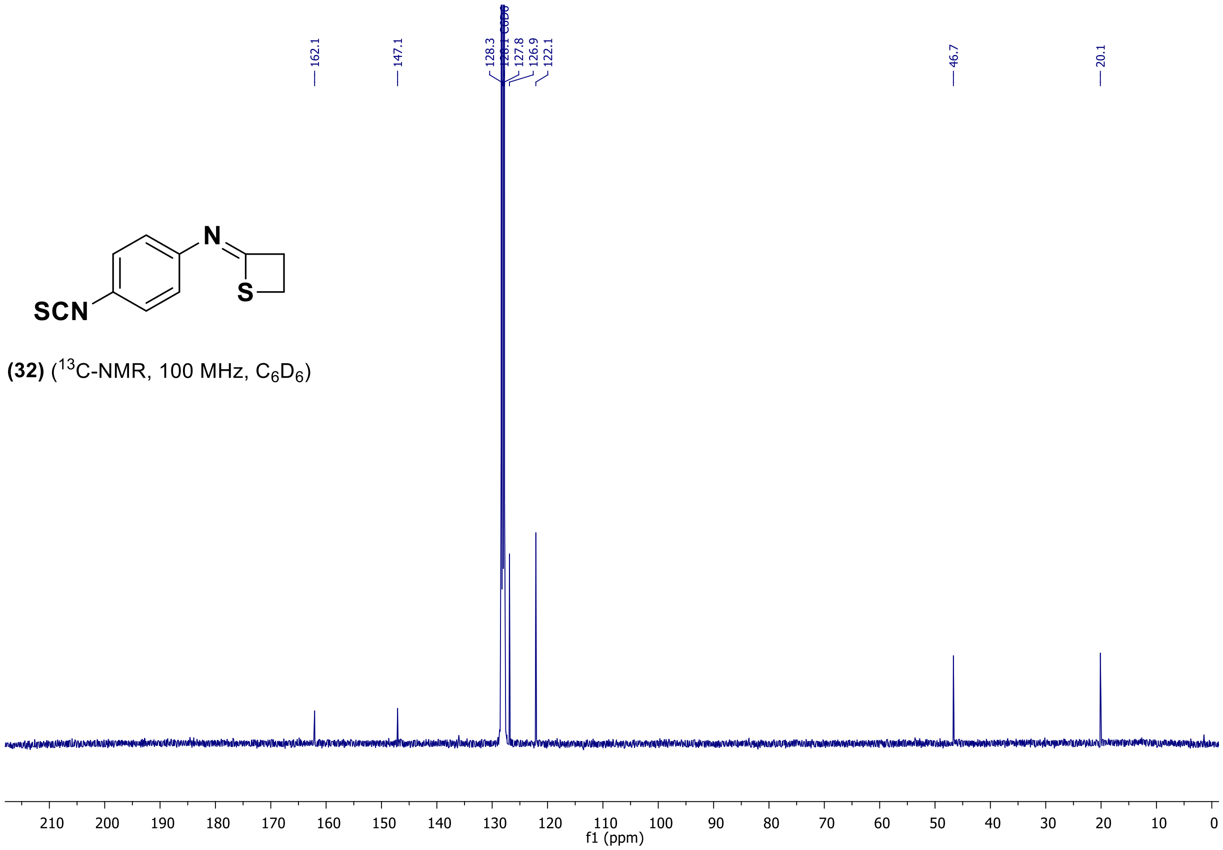

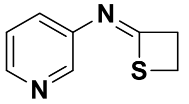

(33) (^{1}H-NMR, 400 MHz, C_6D_6)

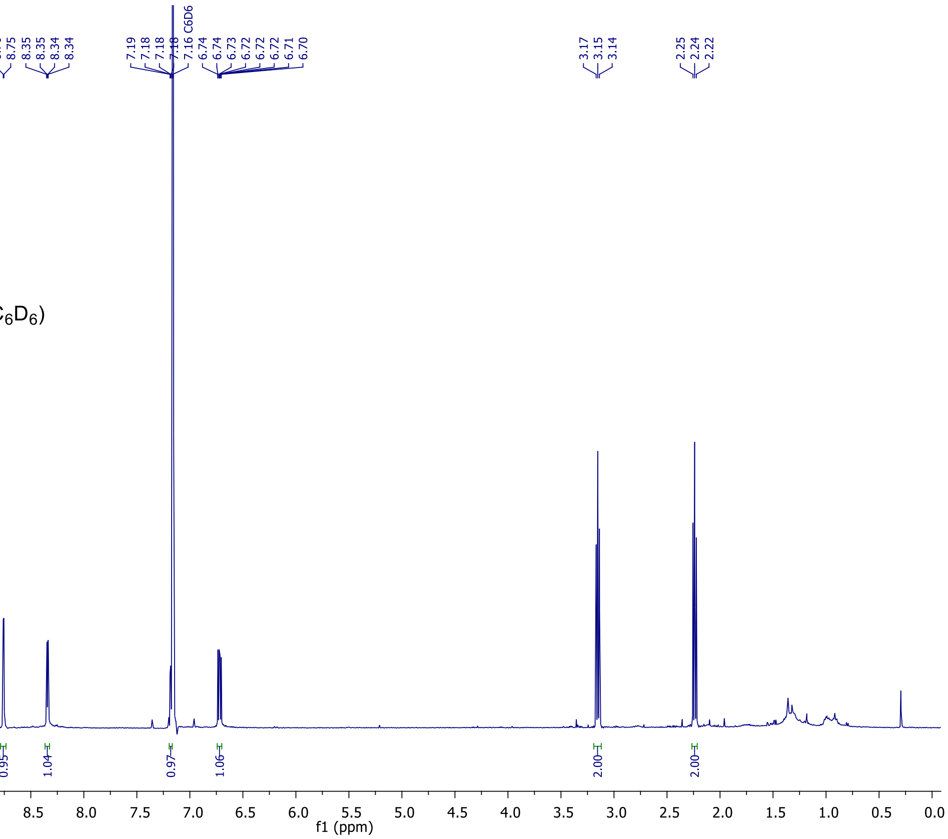

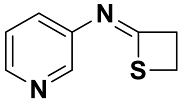

(33) (^{13}C-NMR, 100 MHz, C_6D_6)

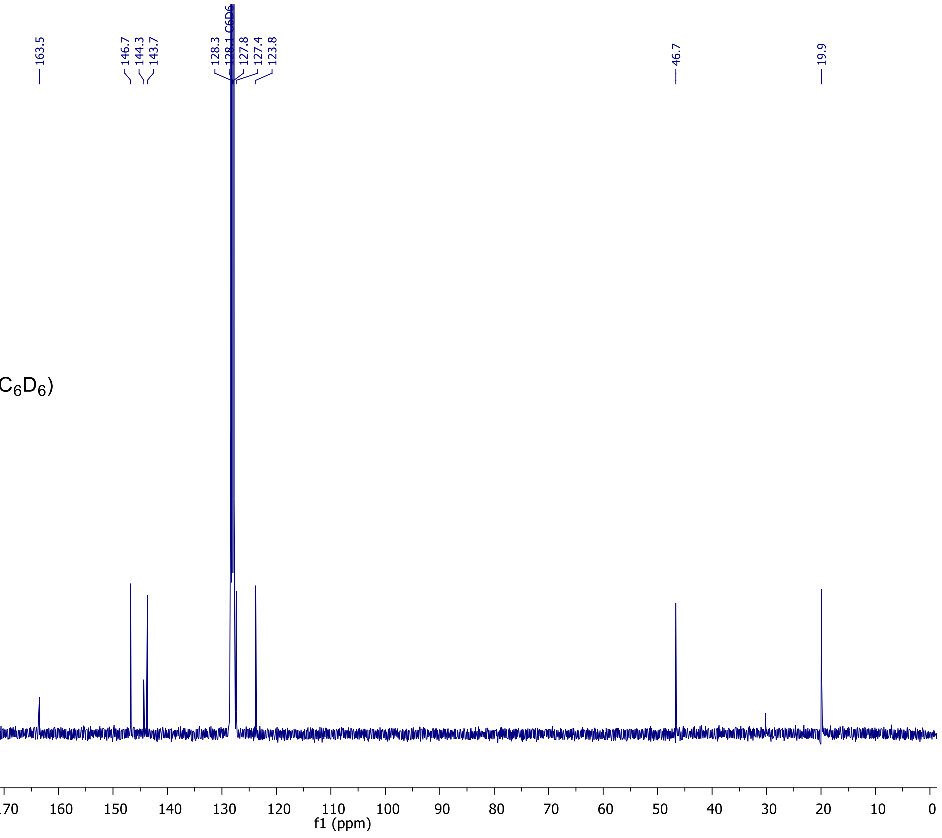

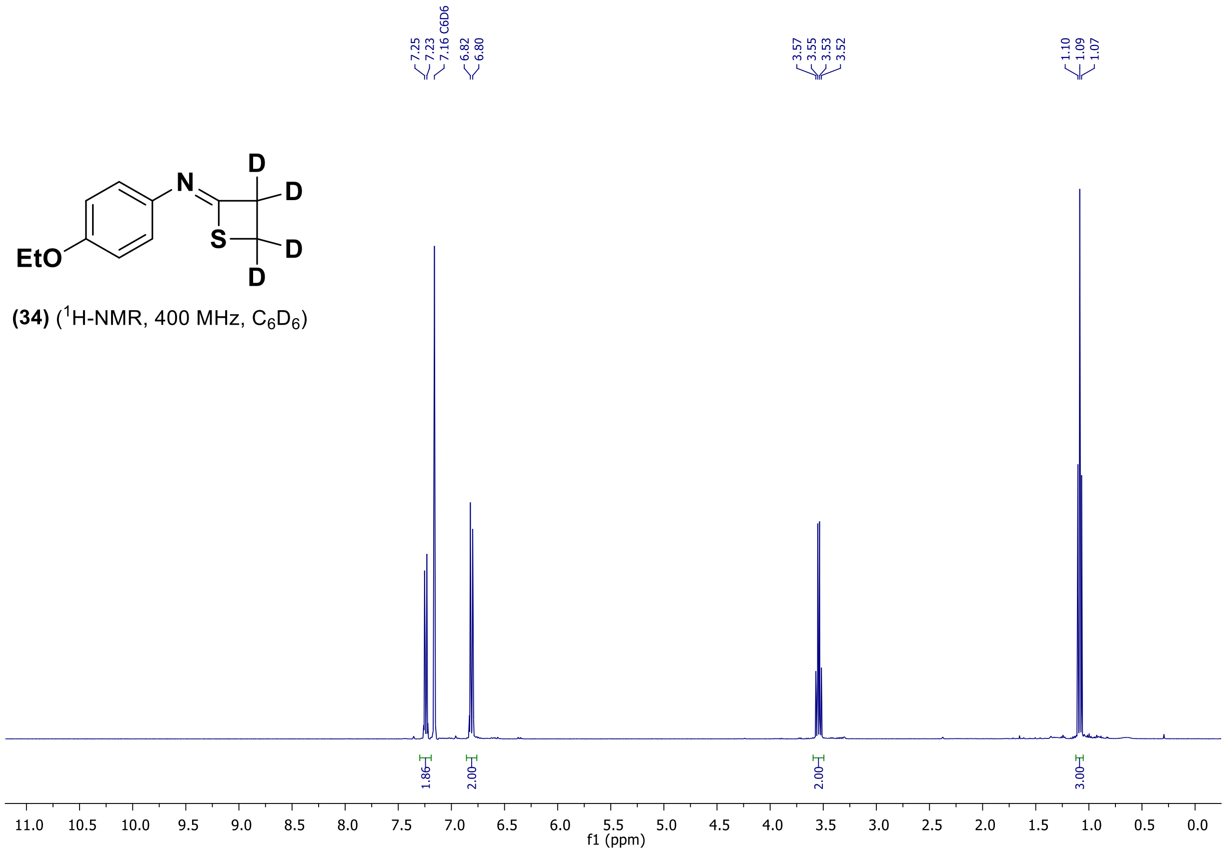

(34) (^{1}H-NMR, 400 MHz, C_6D_6)

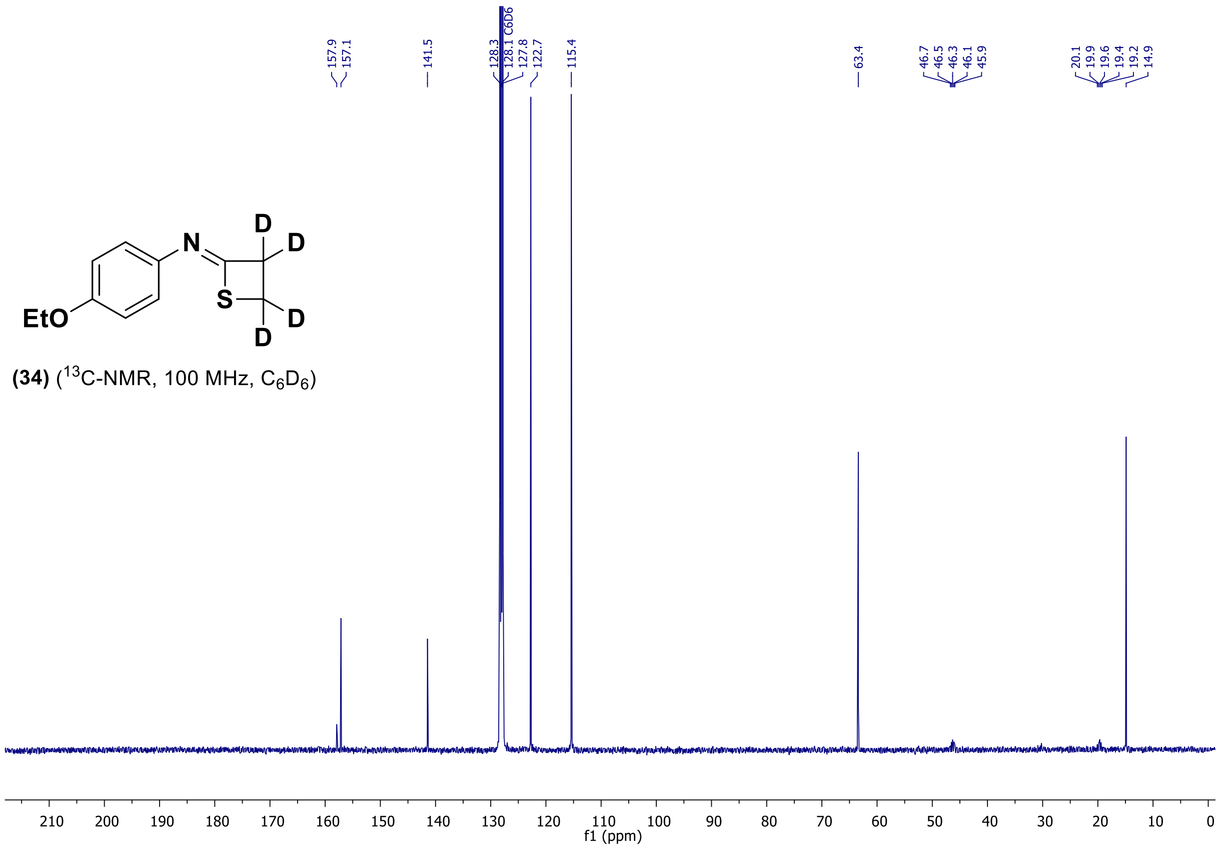

(34) (^{13}C-NMR, 100 MHz, C_6D_6)

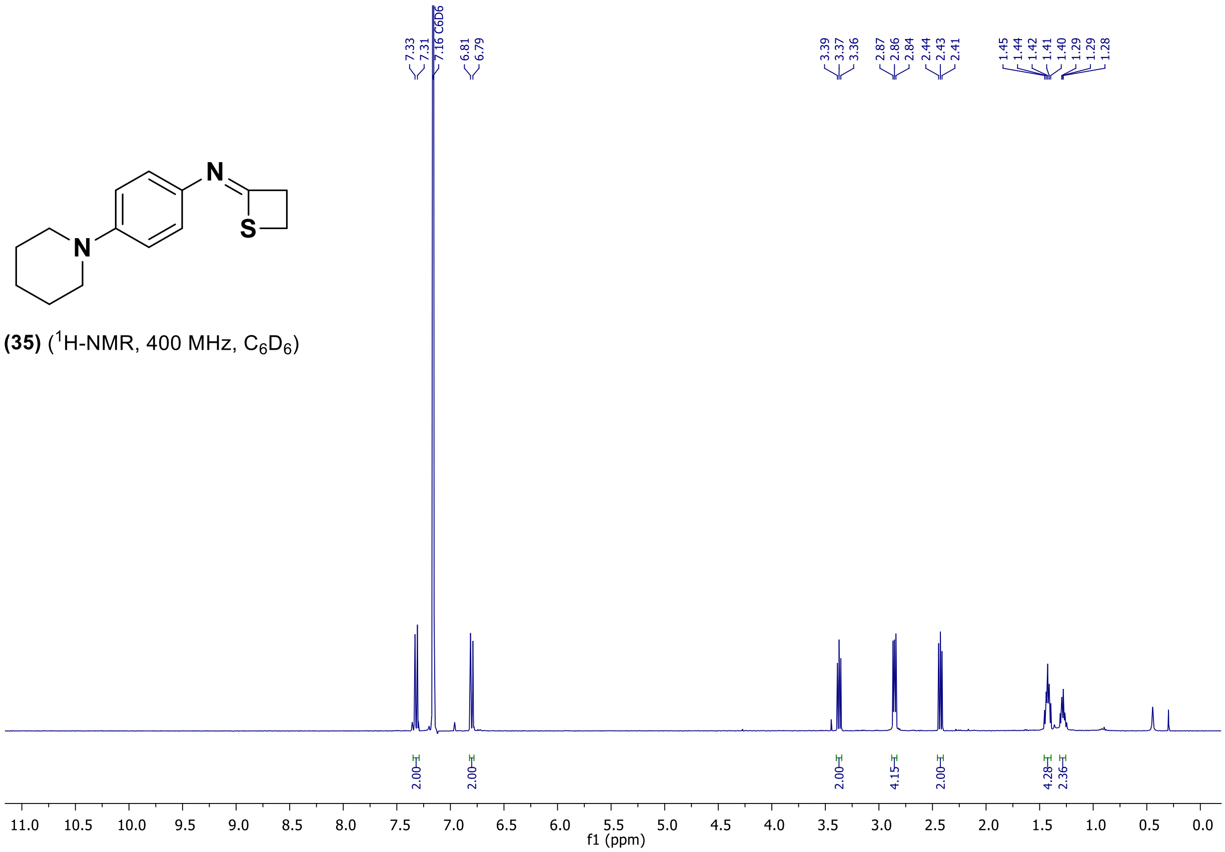

(35) (^{1}H-NMR, 400 MHz, C_6D_6)

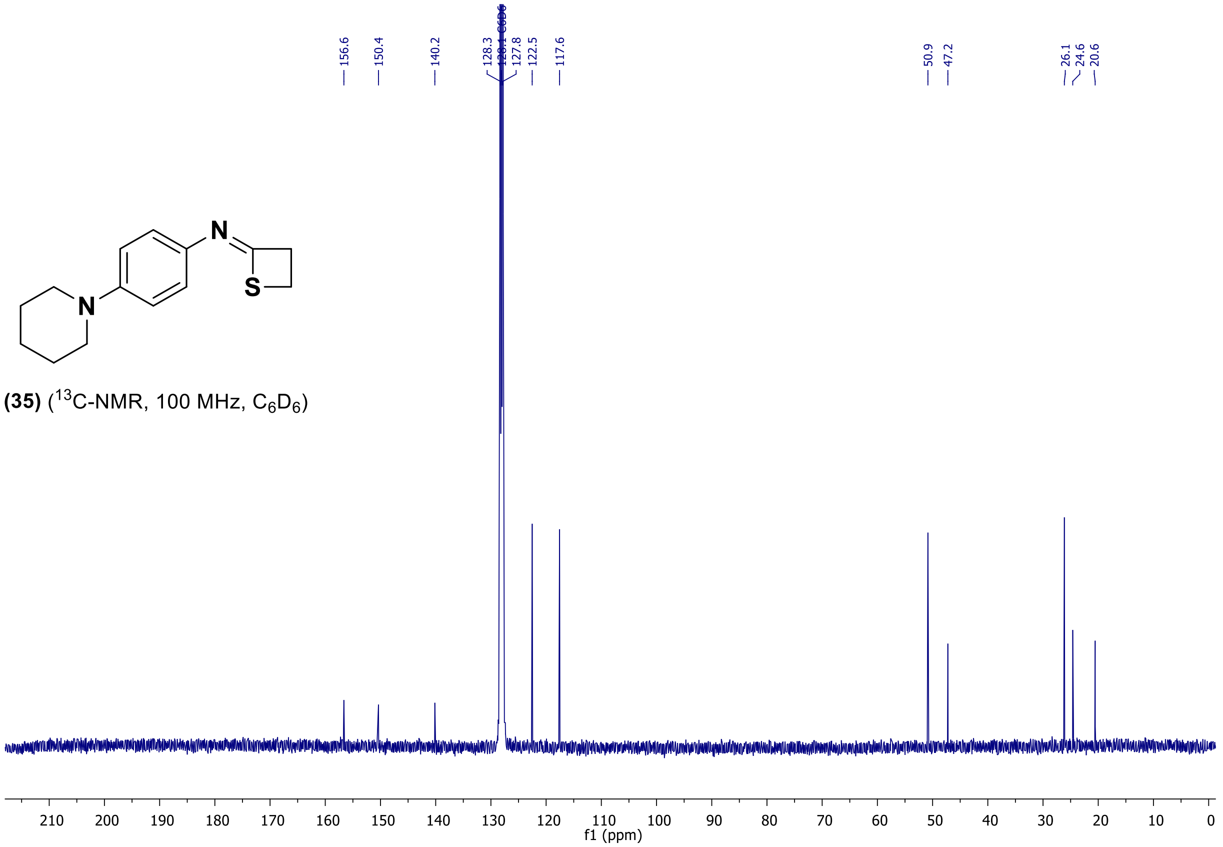

(35) (^{13}C-NMR, 100 MHz, C_6D_6)

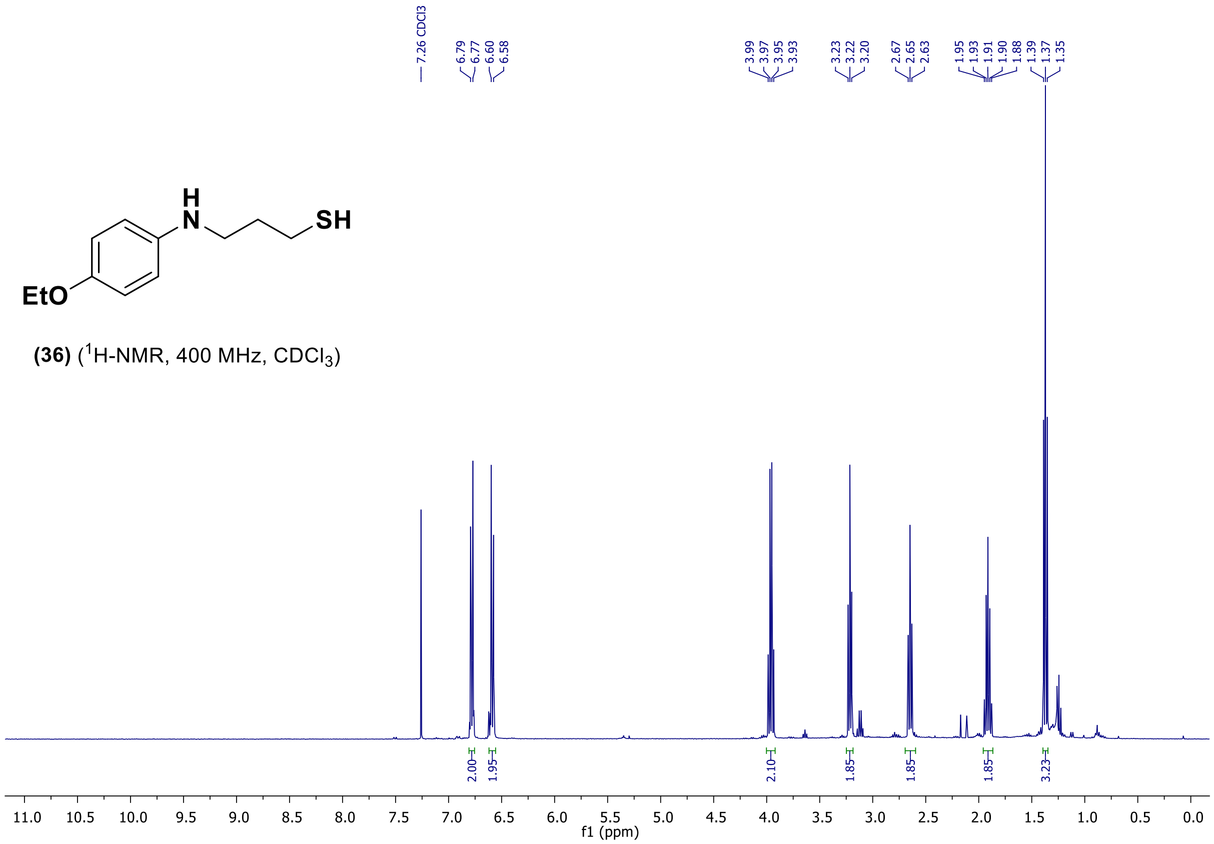

(36) (^{1}H-NMR, 400 MHz, $CDCl_3$)

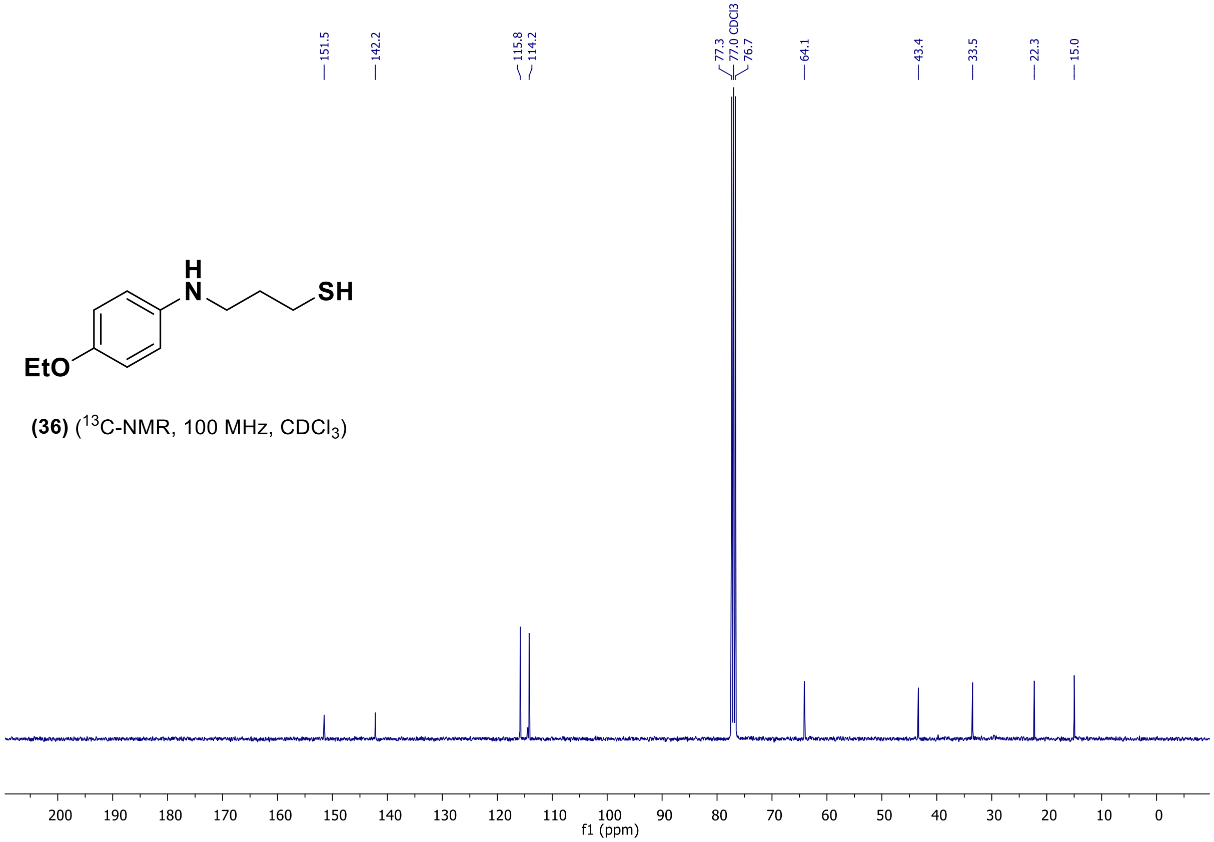

(36) (^{13}C-NMR, 100 MHz, $CDCl_3$)

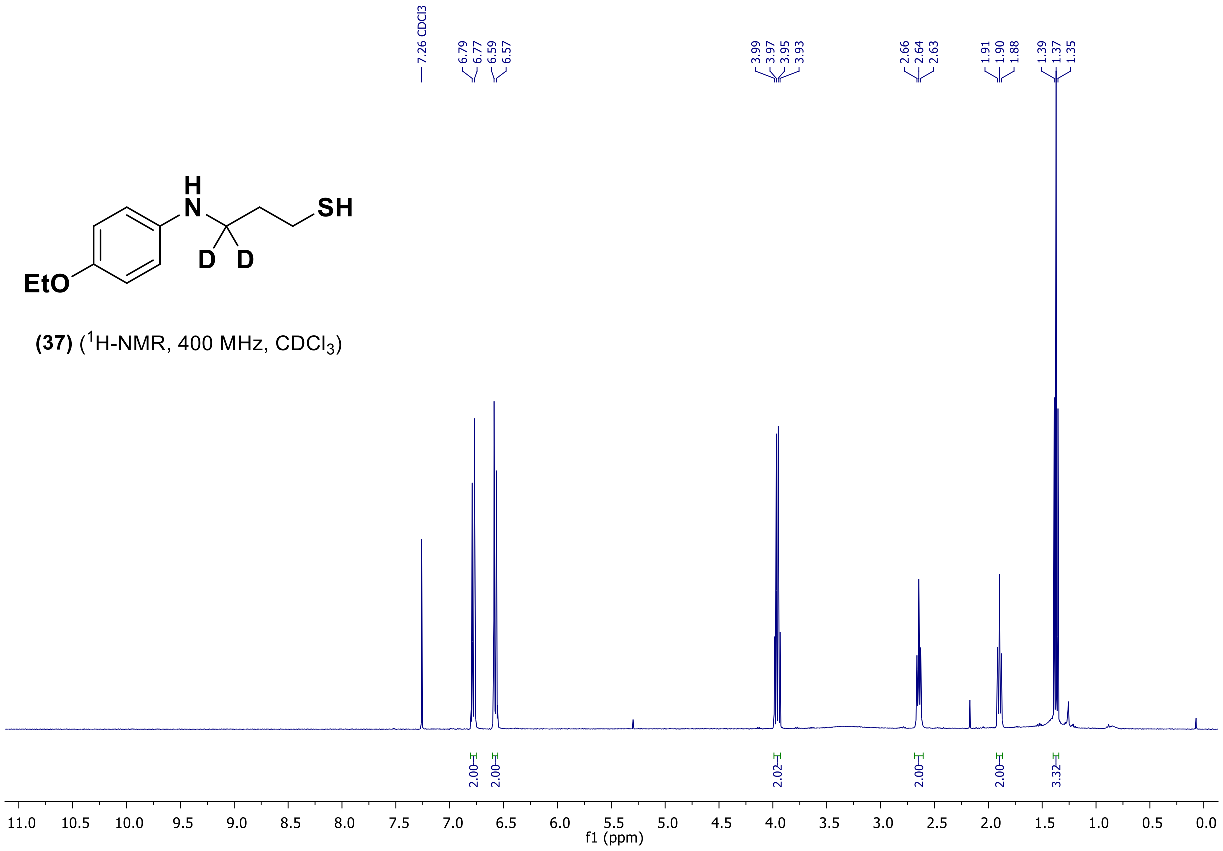

(37) (^{1}H-NMR, 400 MHz, $CDCl_3$)

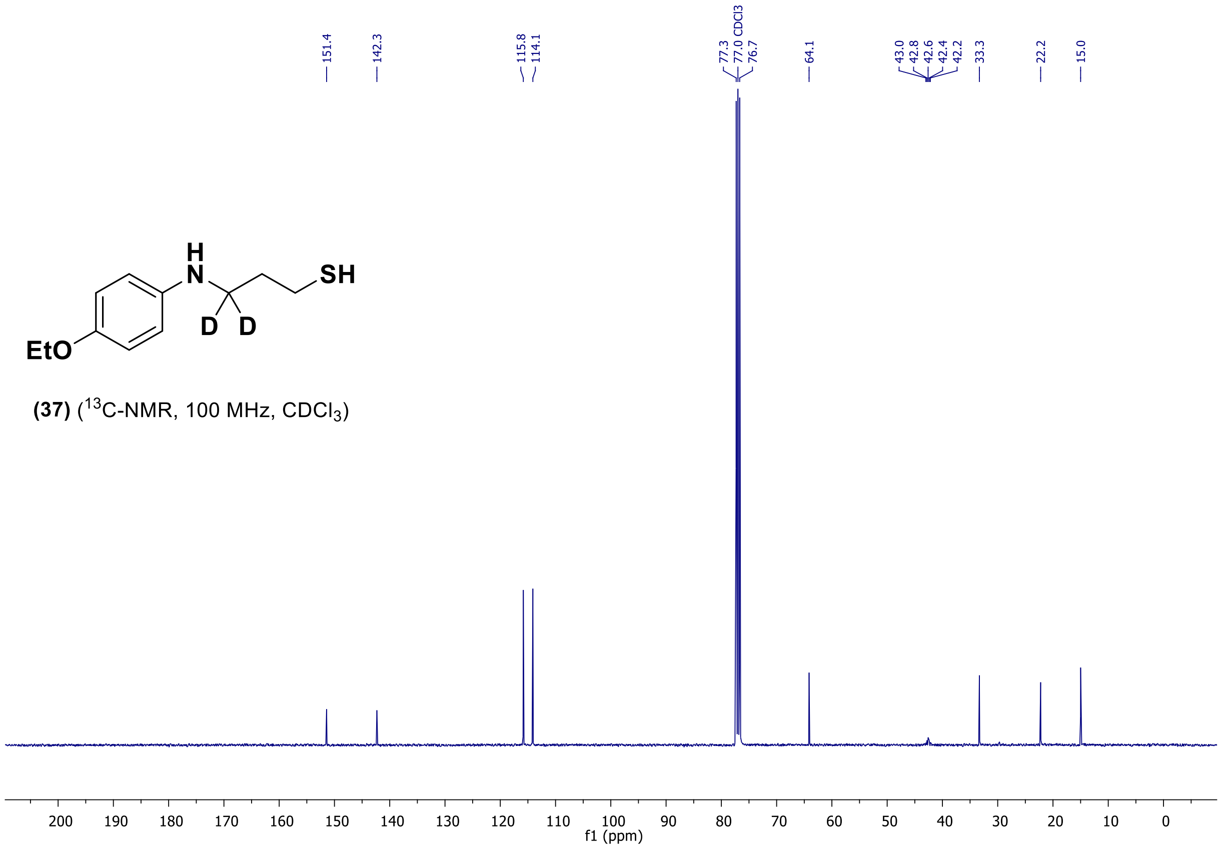

(37) (^{13}C-NMR, 100 MHz, $CDCl_3$)

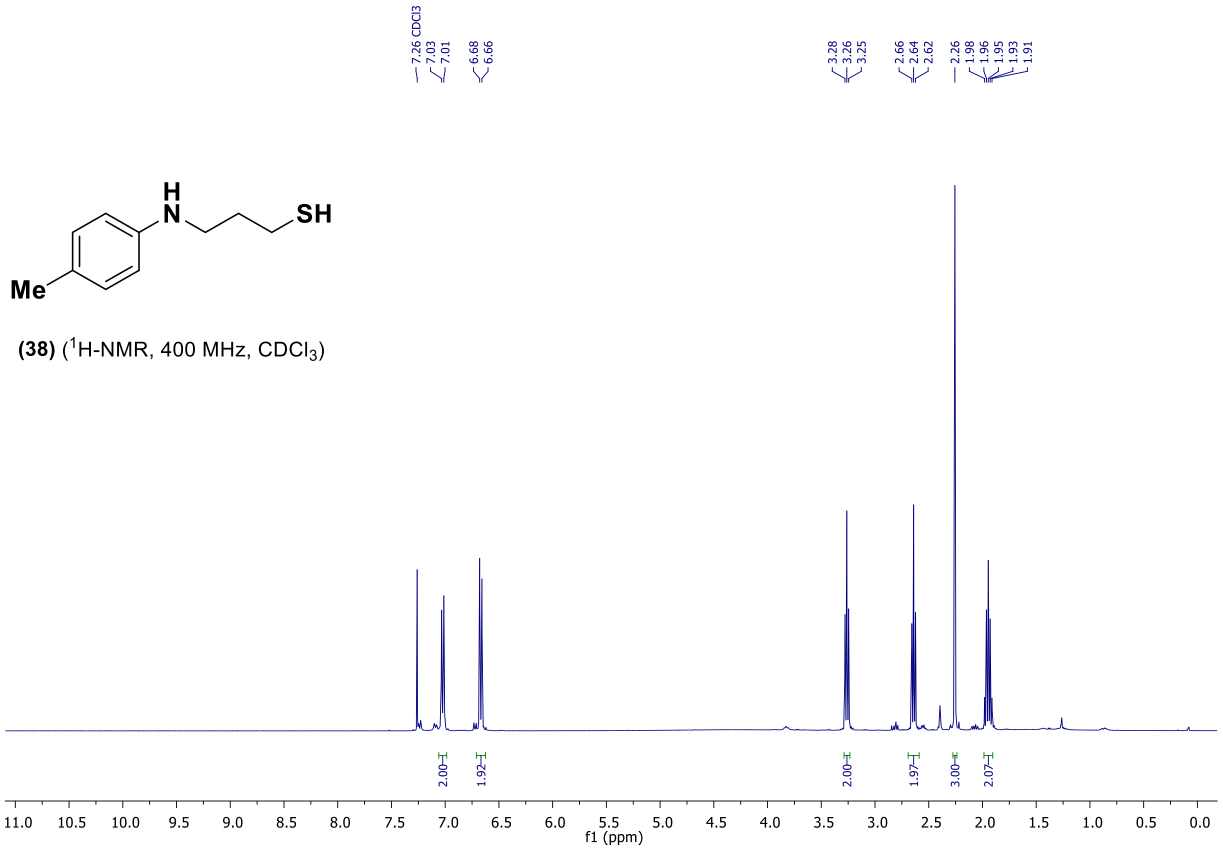

H
N
SH
Me
(38) (^{1}H-NMR, 400 MHz, $CDCl_3$)
7.26 CDCl3
7.03
7.01
6.68
6.66
3.28
3.26
3.25
2.66
2.64
2.62
2.26
1.98
1.96
1.95
1.93
1.91
2.00
1.92
2.00
1.97
3.00
2.07
f1 (ppm)

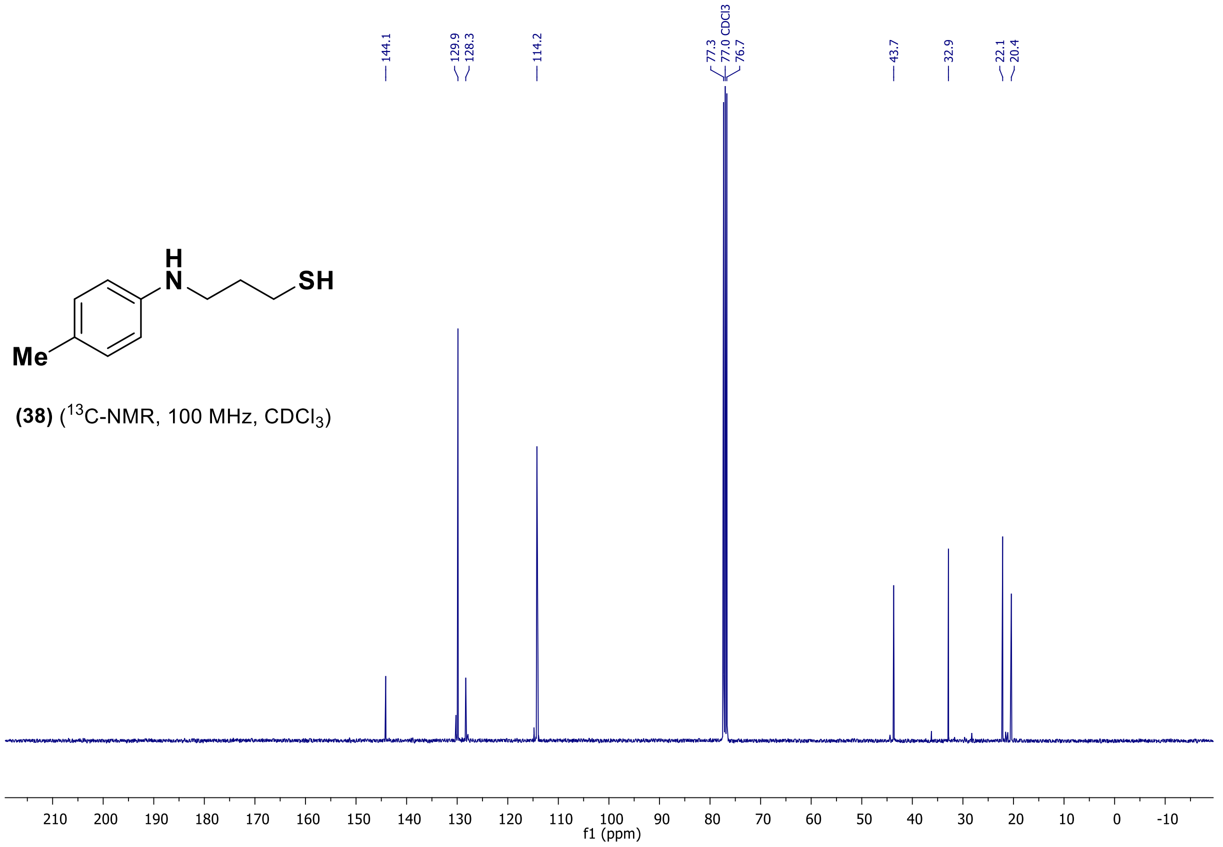

H
N
SH
Me
(38) (^{13}C-NMR, 100 MHz, $CDCl_3$)
144.1
129.9
128.3
114.2
77.3
77.0 CDCl3
76.7
43.7
32.9
22.1
20.4
f1 (ppm)

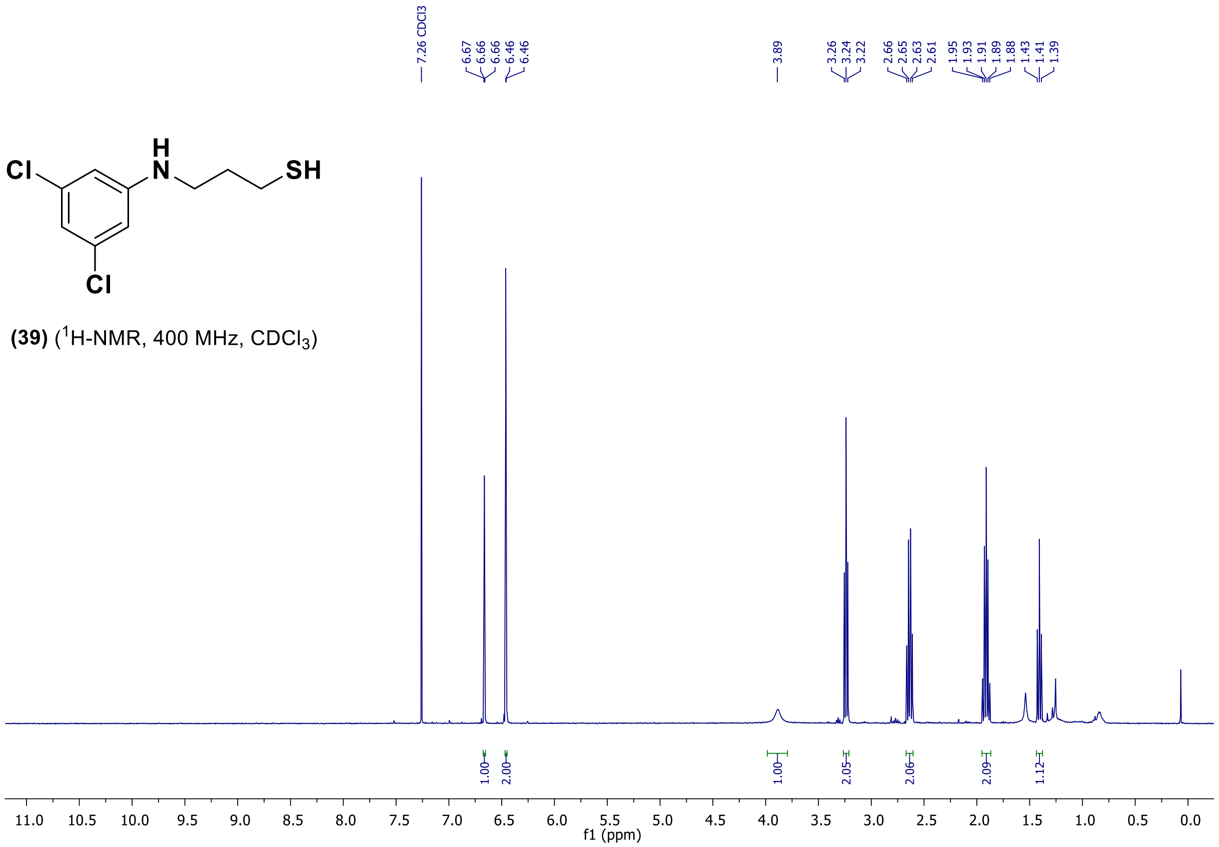

(39) (^{1}H-NMR, 400 MHz, $CDCl_3$)

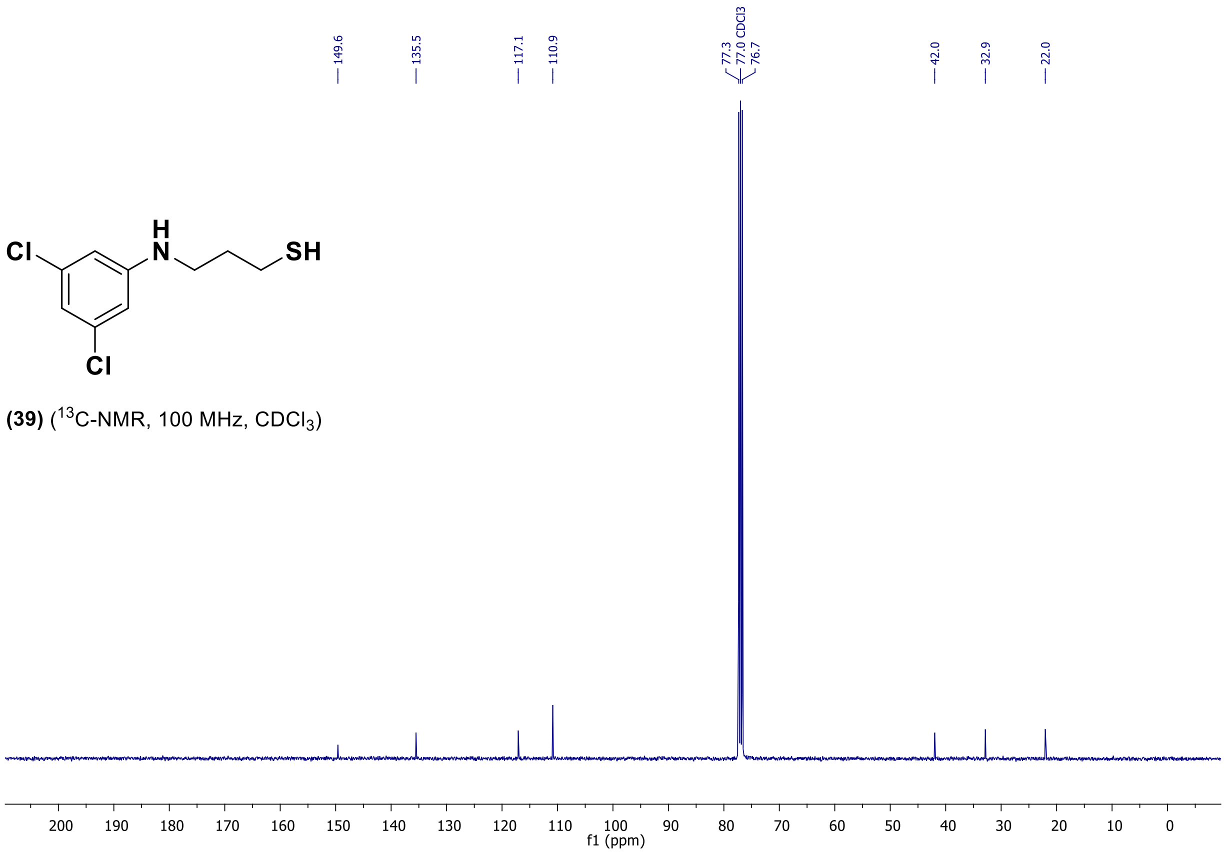

(39) (^{13}C-NMR, 100 MHz, $CDCl_3$)

Copies of ^{19}F- and ^{77}Se-NMR Spectra

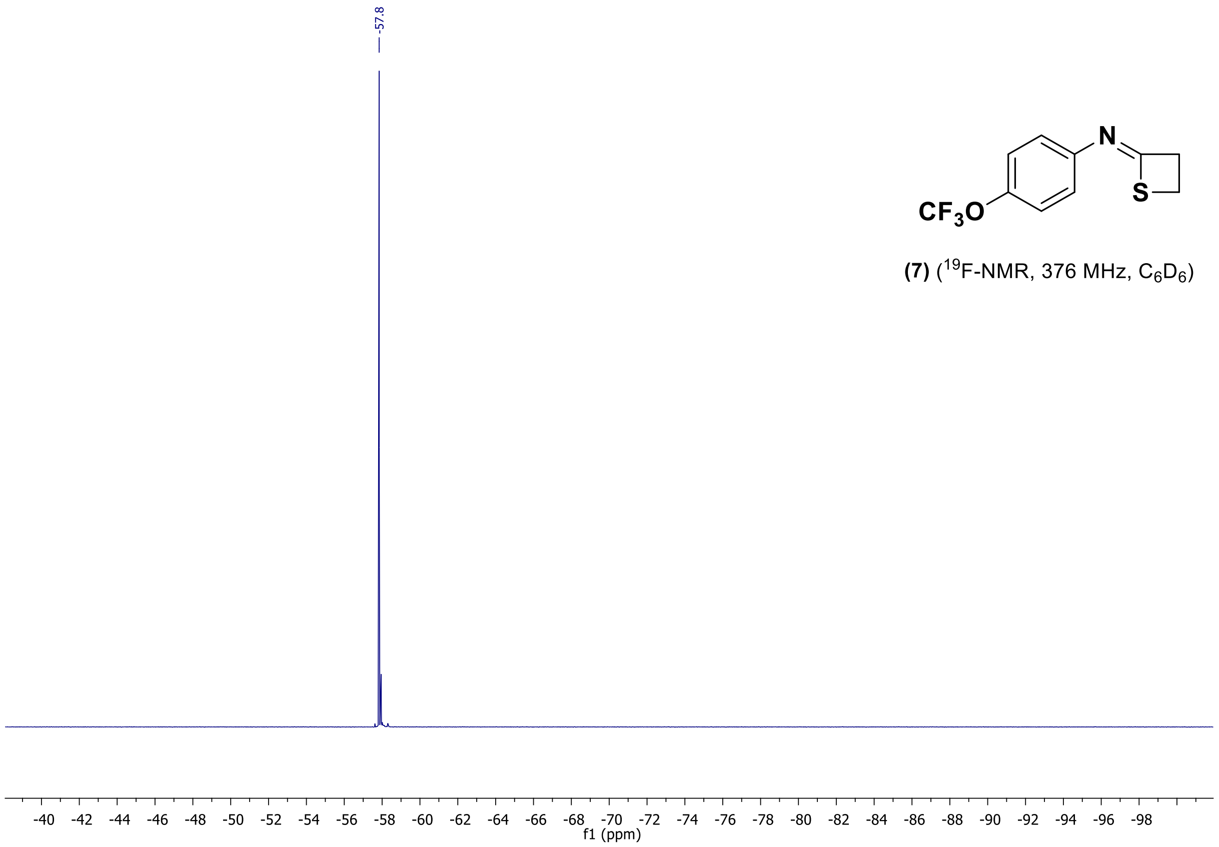

(7) (^{19}F-NMR, 376 MHz, C_6D_6)

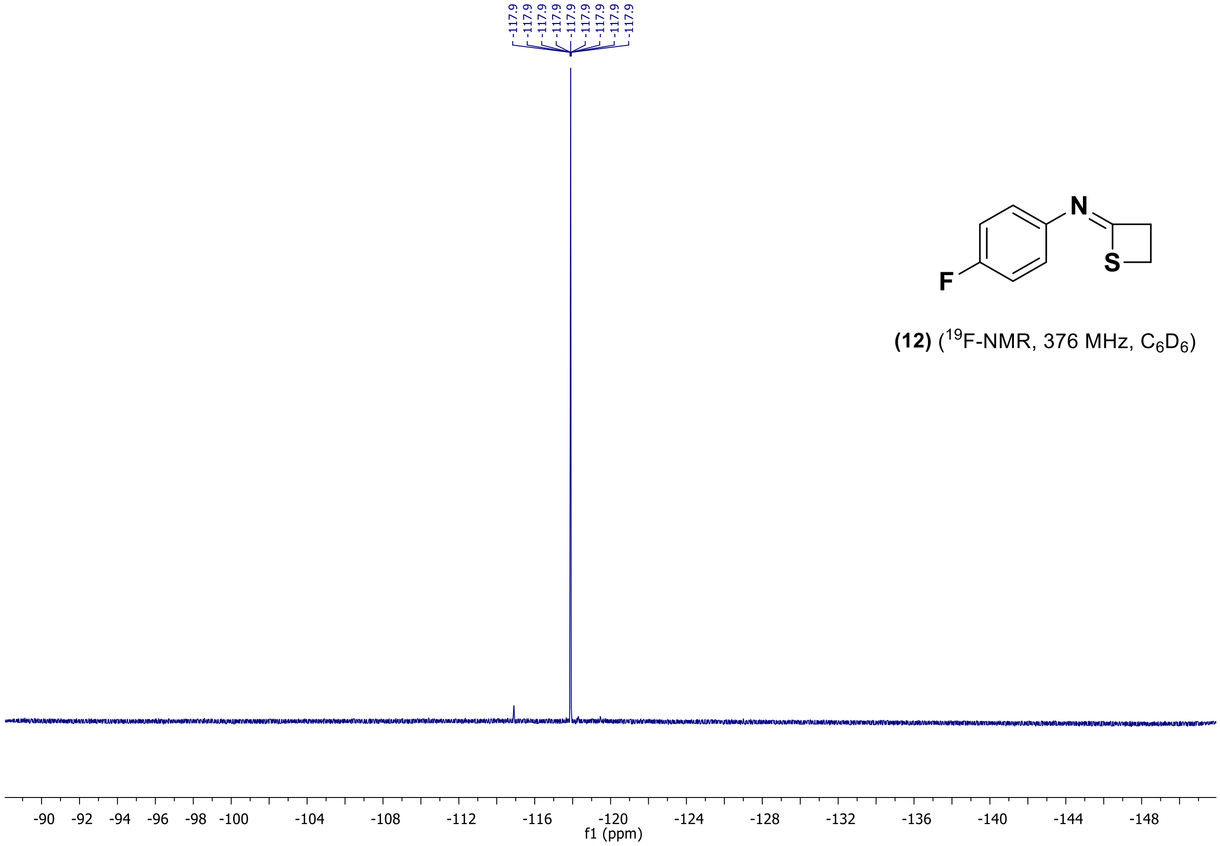

(12) (^{19}F-NMR, 376 MHz, C_6D_6)

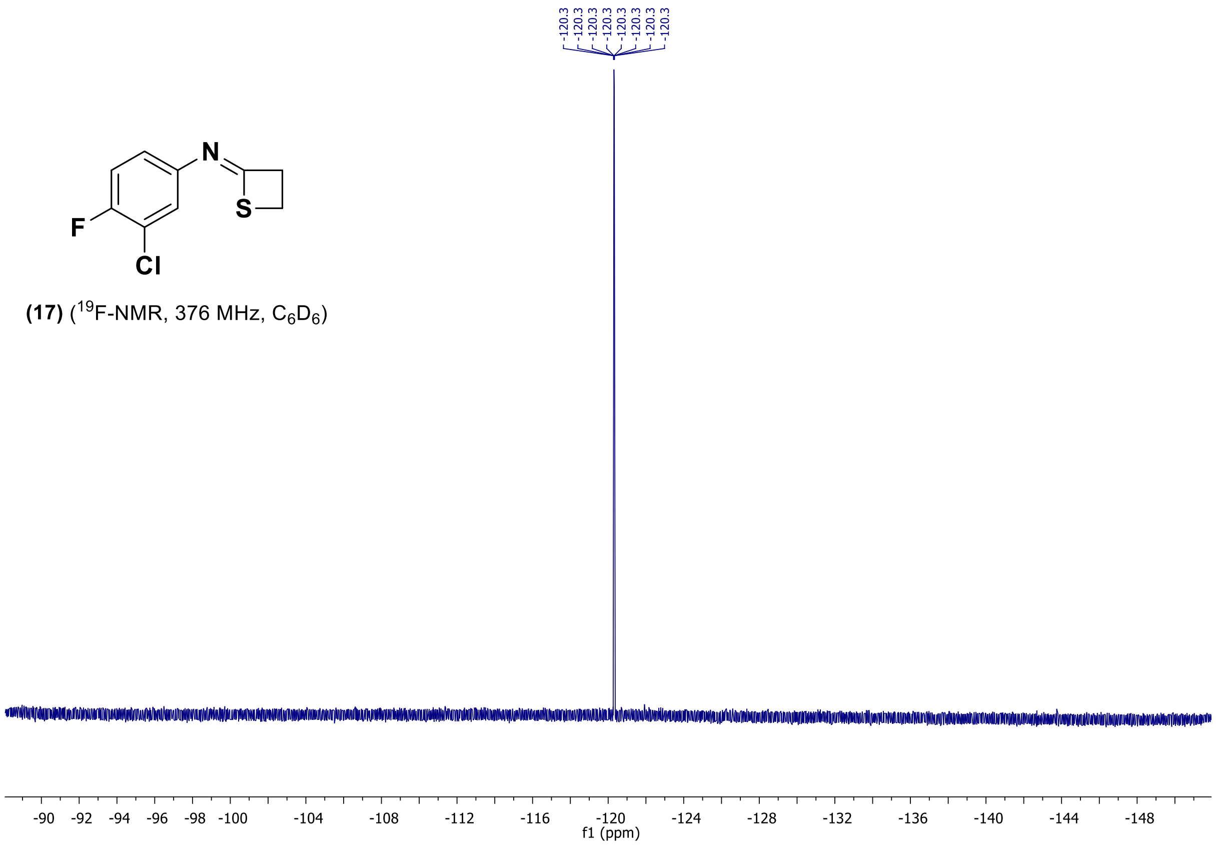

(17) (^{19}F-NMR, 376 MHz, C_6D_6)

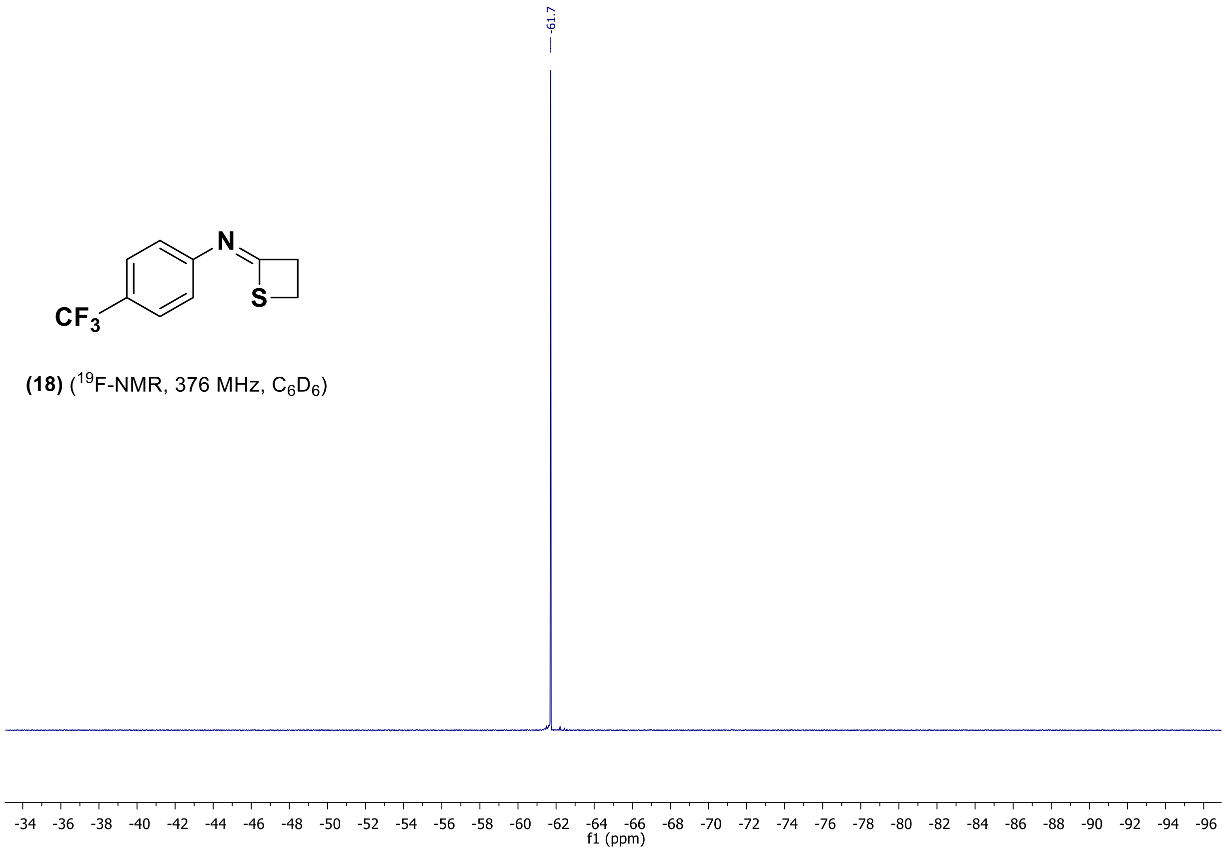

(18) (^{19}F-NMR, 376 MHz, C_6D_6)

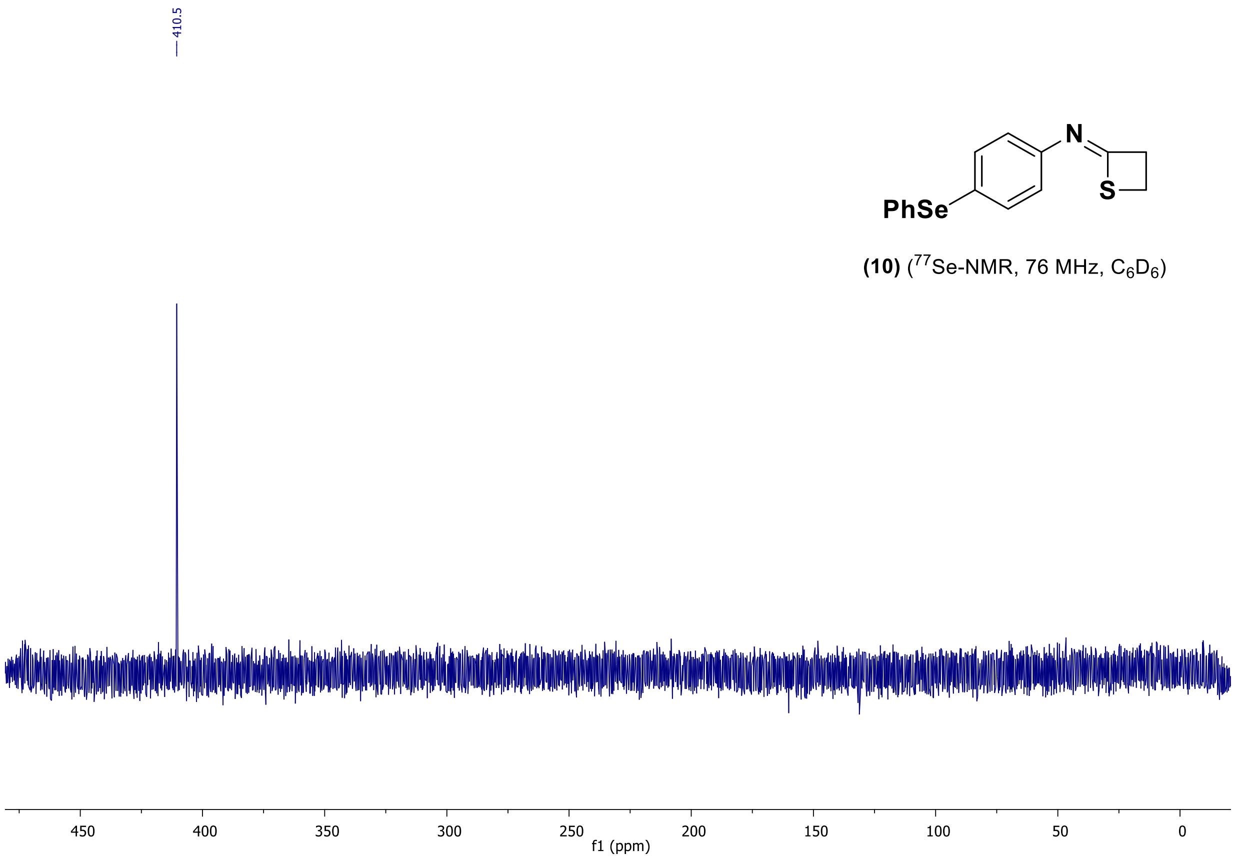

(10) (^{77}Se-NMR, 76 MHz, C_6D_6)

X-ray Analysis for Compound 2

The X-ray intensity data were measured on Bruker D8 Venture diffractometer equipped with multilayer monochromator, Mo K/α INCOATEC micro focus sealed tube and Oxford cooling system. The structure was solved by *Direct Methods*. Non-hydrogen atoms were refined with *anisotropic displacement parameters*. Hydrogen atoms were inserted at calculated positions and refined with riding model. The following software was used: *Bruker SAINT software package*[3] using a narrow-frame algorithm for frame integration, *SADABS*[4] for absorption correction, *OLEX*[5] for structure solution, refinement, molecular diagrams and graphical user-interface, *Shelxle*[6] for refinement and graphical user-interface *SHELXS-2015*[7] for structure solution, *SHELXL-2015*[7] for refinement, *Platon*[8] for symmetry check. Experimental data and CCDC-Codes Experimental data (Available online: http://www.ccdc.cam.ac.uk/conts/retrieving.html) can be found in Table 1. Crystal data, data collection parameters, and structure refinement details are given in Tables 2 to 3. Asymmetric Unit visualized in Figure 1.

Table 1 Experimental parameter and CCDC-Code.

Sample	Machine	Source	Temp.	Detector Distance	Time/ Frame	#Frames	Frame width	CCDC
	Bruker		[K]	[mm]	[s]		[°]	
Compound **2**	D8	Mo	150	40	60	1511	0.500	**2081745**

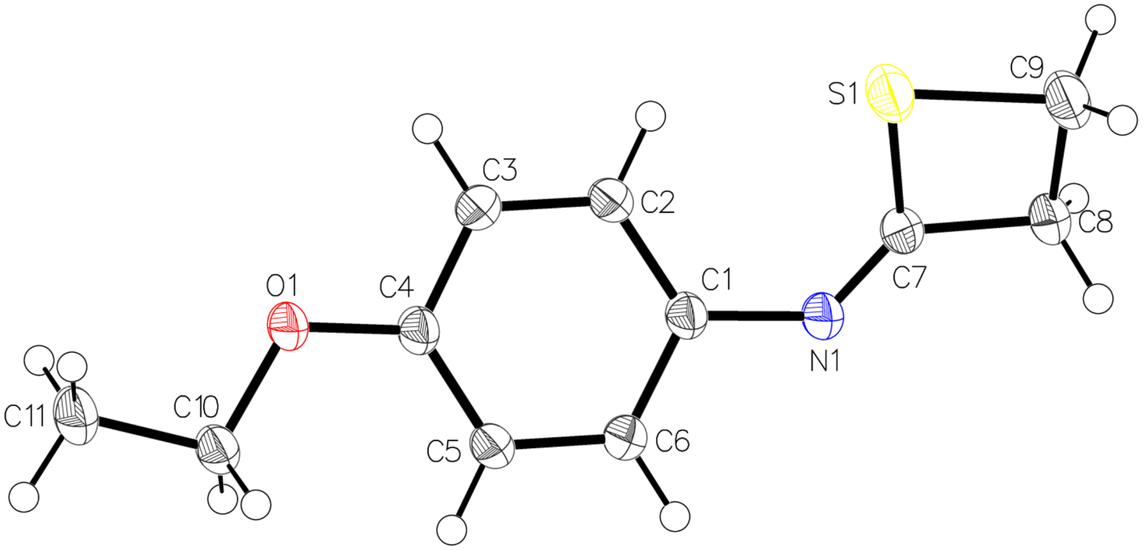

Figure 1 Crystal structure, drawn with 50% displacement ellipsoid. The bond precision for C-C single bonds is 0.0021Å.

Table 2 Sample and crystal data.

Radiation [Å]	MoKα (λ = 0.71073)	Z	2	Measurement method	\f and \w scans
Crystal habit	clear colourless block	a [Å]	6.7458(9)		
Crystal size [mm³]	0.6 × 0.04 × 0.03	b [Å]	7.2758(10)	Abs. correction type	multiscan
Empirical formula	C11H13NOS	c [Å]	82.878(6)	Abs. correction Tmin	0.5985
Formula weight [g/mol]	207.28	α [°]	82.878(6)	Abs. correction Tmax	0.7460
Temperature [K]	150.0	β [°]	80.158(5)	Density (calculated) [g/cm3]	1.344
Crystal system	Triclinic	γ [°]	78.337(6)	Absorption coefficient [mm^{-1}]	0.281
Space group	P-1	Volume [Å³]	512.30(12)	F (000) [e^-]	220.0

Table 3 Data collection and structure refinement.

2Θ range for data collection [°]	5.742 to 60.16	Index ranges		Goodness-of-fit on F^2	1.035
Reflections collected	12143	h	-9 ≤ h ≤ 9	Diff. peak and hole [$e^-Å^{-3}$]	0.29/-0.26
Data / restraints / parameters	2868/0/128	k	-10 ≤ k ≤ 9		
Refinement method	Direct Methods	l	-15 ≤ l ≤ 15	Function minimized	Σ w $(F_o^2 - F_c^2)^2$
		all data	R1 = 0.0680, wR2 = 0.1038	Weighting scheme	where
		I>2σ(I)	R1 = 0.0437, wR2 = 0.0929	$w=1/[\sigma^2(Fo^2) + (0.0364P)^2 + 0.2642P]$	$P=(F_o^2+2F_c^2)/3$

References

[1] P. J. Roth, P. Theato, in *Non-Conventional Functional Block Copolymers*, Vol. 1066, American Chemical Society, 2011, pp. 23-37.
[2] Z. Fu, W. Yuan, N. Chen, Z. Yang, J. Xu, *Green Chem.* **2018**, *20*, 4484-4491.
[3] Bruker SAINT V8.38B Copyright © 2005-2019 Bruker AXS.
[4] G. M. Sheldrick, **1996**, SHELXS. University of Göttingen, Germany.
[5] O. V. Dolomanov, L. J. Bourhis, R. J. Gildea, J. A. K. Howard, H. Puschmann, *J. Appl. Crystallogr.* **2009**, *42*, 339-341.
[6] C. B. Hübschle, G. M. Sheldricka, B. Dittricha, *J. Appl. Cryst.* **2011**, *44*, 1281-1284.
[7] G. M. Sheldrick, *Acta Cryst.* **2015**, *C71*, 3-8.
[8] A. Spek, *Acta Cryst. D* **2009**, *65*, 148-155.
